# Rapid Optimization Enabled by Single-Molecule Tracking: Discovery of a Potent RUVBL1/2 Inhibitor to Evaluate the Targeting of MYC-Driven Cancers

**DOI:** 10.1021/acs.jmedchem.5c03692

**Published:** 2026-04-22

**Authors:** Li Zheng, Eugene Park, Jason Lenihan, William S. R. Forrest, Xin Zhou, Charmaine Fong, Yangzhong Tang, Marcus P. Kelly, Amine Driouchi, Ali Tabatabaei, Helen Wong, Jesse D. Vargas, Samuel T. Albright, Zachary Howard, Maité B. Silva, Liam A. Elliott, Michael Farley, José Ortega, Stephen Jones, Xiao Chang, Taylor Heuer, Quan Zheng, Huntly M. Morrison, Daniel Bracho, Qian Du, Jennifer Le, Abhijit Tarafder, Grzegorz Nawrocki, Patric Schyman, Lakshmi Akella, Mai K. Nguyen, Daisy Ding, Arnold Tao, Fernando Rodríguez Pérez, Kayla VanBuren, Rohit Malik, Melissa Dumble, Daniel J. Anderson, Leah Cleary, David W. Piotrowski, Hilary P. Beck

**Affiliations:** 644092 Eikon Therapeutics Inc., 230 Harriet Tubman Way, Millbrae 94030, California, United States

## Abstract

RuvB-like 1 (RUVBL1) and RuvB-like 2 (RUVBL2) are AAA ATPases that form hetero-oligomeric complexes involved in diverse cellular functions. Increasing evidence implicates the RUVBL1/2 complex as an essential cofactor of MYC, with RUVBL1/2 inhibition reducing c-MYC levels in vitro. Herein, we report a potent RUVBL1/2 inhibitor discovered through a Single-Molecule Tracking (SMT)-driven SAR campaign. Compared with a biochemical ADP-Glo assay, which exhibited limited dynamic range and poor reproducibility under our experimental conditions, the live-cell high-throughput RUVBL SMT assay provided robust and reproducible potency measurements and correlated strongly with cell viability and MYC degradation. Multiparameter optimization yielded compound 18, which demonstrated improved efficacy in a MYC-dependent Burkitt lymphoma xenograft model at a significantly lower dose than the RUVBL1/2 inhibitor CB-6644. This work establishes SMT as a powerful tool to facilitate the drug discovery SAR campaigns and evaluates the therapeutic potential of RUVBL1/2 inhibition in MYC-dependent cancers.

## Graphical abstract

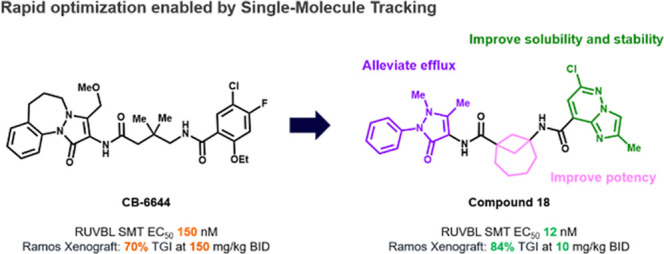

## 1 Introduction

RUVBL1 (also known as RuvB-like 1, Rvb1, Pontin, or TIP49) alongside the paralog RUVBL2 (RuvB-like 2, Rvb2, Reptin, or TIP48) are evolutionarily conserved members of the AAA (ATPases Associated with diverse cellular Activities) superfamily, where in contrast to other AAA family members such as VCP/p97, the ATPase activities of RUVBL1 and RUVBL2 (RUVBL1/2) are comparatively the least pronounced.^1–3^ Respectively exhibiting minimal ATPase activity alone, coassembly of heterohexameric RUVBL1/2 and subsequent dodecameric structures results in enhanced enzymatic function and enables specificity for a wide range of cellular complexes.^2–4^ While other AAA family proteins act as highly processive motors rapidly hydrolyzing ATP to drive a continuous hydrolysis motor, ATP binding of RUVBL1/2 promotes conformational changes enabling molecular switch-like states which subsequently regulate complex formation with a myriad of cofactors.^5,6^ Dynamic engagement by RUVBL1/2 with multiple macromolecular complexes is enabled by the conformational flexibility exhibited from oligomeric structures, which allows for the adoption of both open and closed ring conformations as revealed by Cryo-EM and X-ray crystallography studies.^7,8^ Cofactors binding to the unique Domain II (DII) of RUVBL1/2, regulate ATP hydrolysis and the functional conformation of resultant complexes. The structural role of DII domains within the RUVBL proteins has demonstrated functional autoinhibition of ATPase activity via steric inhibition of the nucleotide binding pocket, wherein DII loss-of-function results in higher ATPase activity.^9^ Despite relatively low ATPase activity, drug discovery efforts have nonetheless utilized the ADP-Glo assay in the hit-identification of selective inhibitors. Several classes of RUVBL1/2 inhibitors with promising preclinical activity have been reported (Figure 1). Among these, aminopyrazolone-based inhibitors were previously disclosed in patent applications from Daiichi Sankyo.^10^ Another aminopyrazolone-based molecule, CB-6644, was subsequently identified as a selective allosteric inhibitor of the RUVBL1/2 complex.^11,12^ Cryo-EM elucidates the allosteric mechanism of action for CB-6644, wherein the inhibitor’s binding at the RUVBL1 and RUVBL2 interface traps an ATP-bound conformation, abrogating progression of ATP hydrolysis.^13^ The previously disclosed RUVBL1 selective chemical matter has demonstrated broad in vitro efficacy across multiple cancer cell-line models and delayed disease progression in MYC-driven acute myeloid leukemia and lymphoma xenograft models, providing strong proof-of-concept for a therapeutic strategy targeting RUVBL1/2.^11,12,14,15^ Subsequent studies have further demonstrated the interaction between RUVBL1/2 and MYC, with pharmacologic inhibition with CB-6644 leading to decreased protein levels of MYC in vitro and delayed tumor progression in murine tumor models highlighting RUVBL1/2 as an important cofactor of MYC.^16^

**Figure 1:**
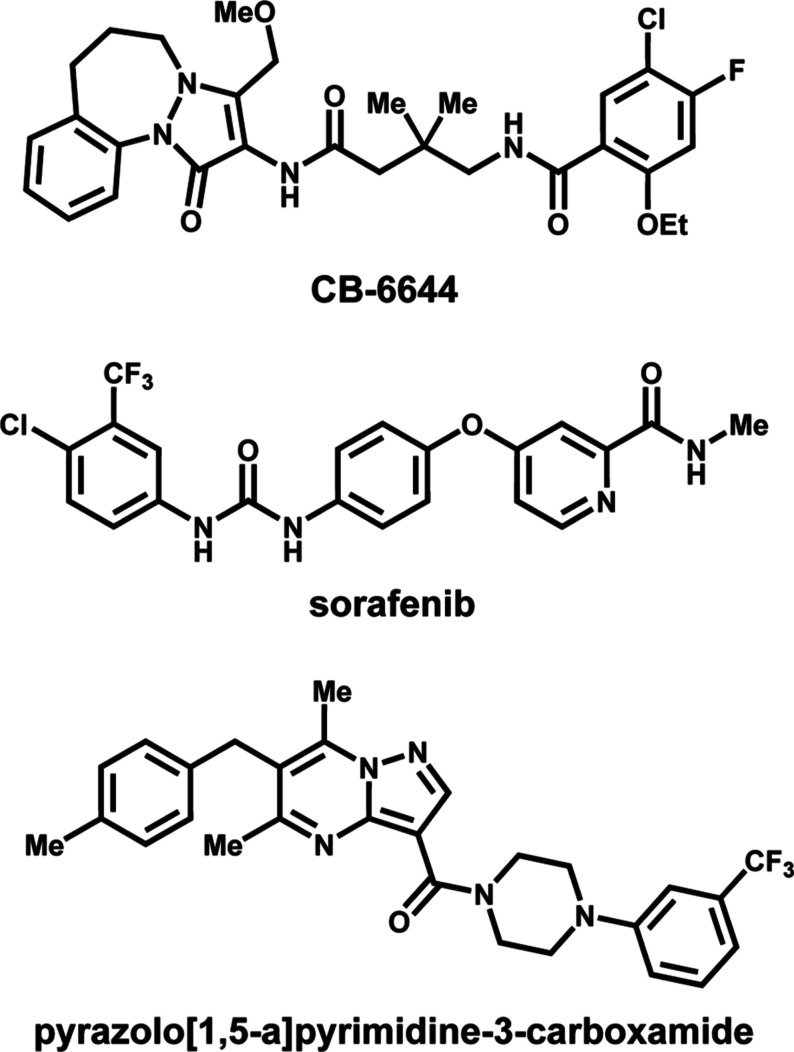
Chemical structures of known inhibitors of RUVBL1/2.

Identified as the first proto-oncogene in the early 1980s, MYC is now recognized as a central driver of cancer progression and therapy resistance across tumor types.^17–21^ Despite a driving role in the progression of a myriad of cancer types, direct pharmacological inhibition of MYC has proven challenging due to its intrinsically disordered transactivation domain and lack of enzymatic activity. Consequently, therapeutic strategies have shifted toward identifying MYC-dependent vulnerabilities utilizing genetic loss-of-function screens.^22–25^ RUVBL1/2 have been independently identified as significant hits from in vitro and in vivo based genetic screening of MYC-driven cancer models, supporting their interaction in the biology of solid tumors.^16,26^ Subsequent studies have further demonstrated the interaction between RUVBL1/2 and MYC, with induced genetic loss-of-function and pharmacologic inhibition of RUVBL1/2, utilizing CB-6644, leading to a decrease in the protein levels of MYC in vitro and delayed tumor progression in murine pancreatic tumor models.^16^ Collectively, multiple studies demonstrating RUVBL as a cofactor of MYC, support the rationale for inhibiting RUVBL in MYC-dependent cancers. Additional RUVBL1/2 inhibitors, including sorafenib and pyrazolo­[1,5-*a*]­pyrimidine-3-carboxamide analogs have also been shown to target the ATPase activity.^27,28^ However, the discovery of more potent inhibitors has been limited by standard ADP- based biochemical assays, which often suffer from a narrow dynamic window and poor reproducibility (Figure S1). These issues stem in part from the difficulty of consistently reconstituting RUVBL1/2 into defined oligomeric states, as different assemblies display heterogeneous activity and sensitivity to inhibition, underscoring the need for alternative assay methodologies. Herein we seek to primarily leverage the protein dynamics of RUVBL1/2 to facilitate the development of novel inhibitors, rather than utilizing ADP-based biochemical assays, to explore the therapeutic potential of this target.

RUVBL1/2 complexes are essential components of higher-order chromatin remodeling assemblies, including the INO80 and SWR1 complexes, which regulate gene expression and maintain genome stability through nucleosome sliding and histone exchange. These processes are increasingly implicated in oncogenic transformation and cancer progression.^29–31^ Additionally, RUVBL1/2 are also core components of the TIP60 acetyltransferase complex^32–35^ which is demonstrated to be capable in forming complexes with the proto-oncogene MYC.^36,37^ A tripartite relationship between MYC and TIP60 complex members, RUVBL1/2 and TRRAP, revealed through chromatin immunoprecipitation studies, enables recruitment of RUVBL1/2 to chromatin by MYC. These macromolecular complexes comprising of RUVBL1/2, including the R2TP/PAQosome, represent functionally dynamic oligomeric states that are orders of magnitude larger than RUVBL1 and 2 monomers (50 and 51 kDa, respectively) with the size of the human TIP60 complex being the largest at ∼1.8 mDa (Figure 2).^34,35^ Equilibrium between different states (hexamers and dodecamers) and participation in numerous transient protein–protein interactions underscore how a compound affecting the target’s mobility would be critical for rationally designed inhibitors of RUVBL1/2 function. We hypothesize that by directly measuring the dynamic behavior of RUVBL1/2 in living cells, we can rapidly identify compounds that modulate their contribution to macromolecular function and gain immediate mechanistic insights inaccessible through conventional biochemical or cell-based end point assays. This approach allows for the screening of novel chemical matter, distinguishing direct binders from indirect pathway modulators based on effect kinetics, and understanding the structure–activity relationships (SAR) to guide lead optimization. Herein we harness RUVBL protein dynamics in our previously described automated high-throughput single-molecule tracking (htSMT) platform for rapid development in the discovery of exceptionally potent RUVBL inhibitors exhibiting improved efficacy in MYC-driven cancer models.^38,39^

**Figure 2:**
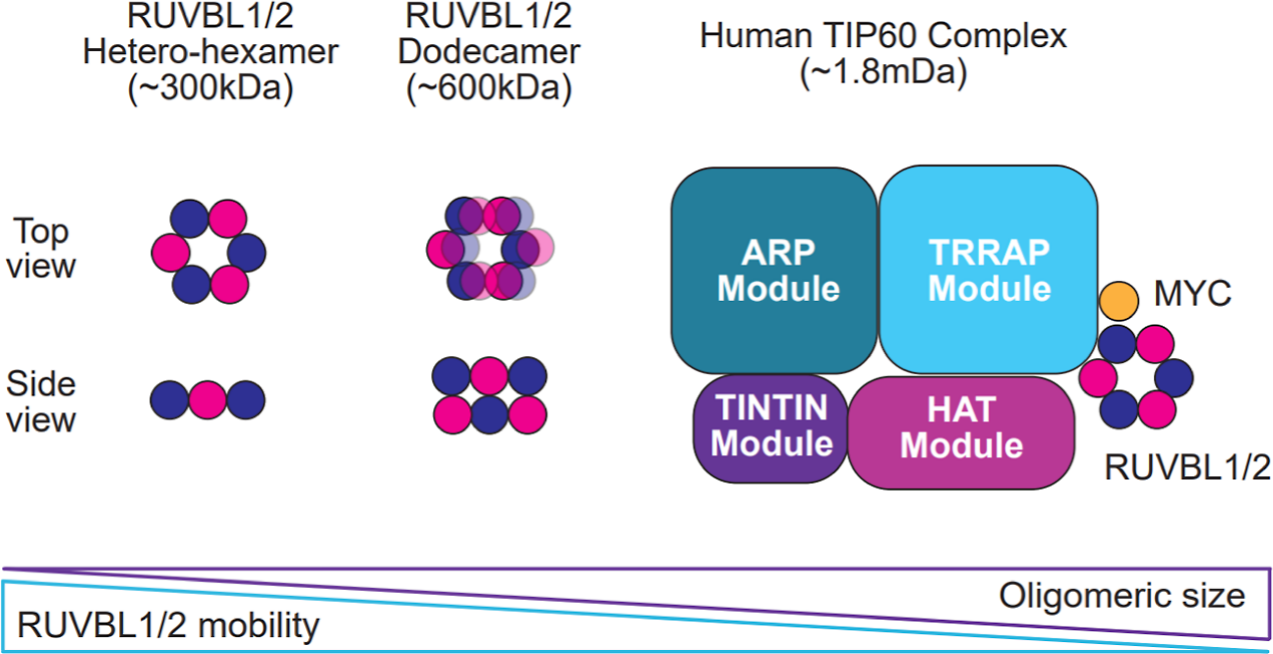
A schematic depicting representative RUVBL1 and RUVBL2 oligomeric states of increasing molecular weight and complexity.^34^

## 2 Results and Discussion

As we set out to develop potent and efficacious RUVBL inhibitors to evaluate their therapeutic potential in the treatment of MYC-driven cancer, we utilized the biochemical ADP-Glo assay to measure RUVBL ATPase activity as a readout of enzymatic inhibition. However, within our experimental conditions this assay exhibited a narrow dynamic range and poor reproducibility (Figure S1), likely due to the challenges of consistently reconstituting the desired multimeric RUVBL1/2 complex from individually purified proteins. While alternative assaying strategies, such as the use of viability assays offer useful cellular readouts, they are suboptimal for driving SAR on their own given that establishing RUVBL1/2-deficient cells for counter-screening is challenging. Understanding that RUVBL1/2 ATPase activity is intrinsically coupled to its assembly into higher-order oligomeric complexes, we sought to determine whether measuring RUVBL1/2 dynamics using SMT could provide a reliable measure of target engagement toward effectively driving molecular evolution.

Our automated htSMT platform enables measurement of protein motion in millions of cells per day across thousands of experimental conditions.^38^ By incorporating a lightsheet-based illumination modality, we recently improved both the throughput and robustness of the htSMT assay.^39^ Following the acquisition of SMT movies, data is processed using a custom analysis pipeline. Fluorescent emitters generated through sparse labeling of individual proteins with Janelia Fluor 549 (JF_549_) are detected, localized with subpixel precision, and linked in time to produce trajectories which are then assigned to subcellular compartments. These trajectories can provide quantitative insights into the assembly and dynamics of higher-order protein complexes. To enable our single molecule tracking assay, we used a landing pad system to generate U2OS cell lines ectopically expressing a RUVBL1-HaloTag fusion protein (RUVBL1^halo^). Prior to using this cell line in our SAR campaign, we confirmed the successful C-terminal tagging of the RUVBL1 protein by Western Blot (Figure S7), which demonstrated the expected shift in molecular weight relative to the untagged protein, in contrast to *N*-terminal tagging. HaloTag and V5 staining further confirmed the expression of the fusion protein. To assess how CB-6644 changes RUVBL1/2 motion, we employed htSMT to track the dynamics of individual RUVBL1/2 proteins inside living cells. Upon treatment with CB-6644, we observed a dose-dependent increase in the nuclear diffusion of RUVBL1 proteins. We also observed a corresponding change in the motion of RUVBL2 proteins using an analogous SMT assay in a RUVBL2^halo^ cell line, demonstrating that tracking the motion of either protein in the complex can be used to measure compound activity (Figure 3a). Treatment with CB-6644 induced no change in the motion of VCP HaloTag fusion proteins, another AAA ATPase, demonstrating the specificity of CB-6644 to perturb RUVBL1/2 dynamics (Figure S9a). To better understand the relationship between RUVBL1/2 inhibition and its motion, we generated a state-array histogram showing the distribution of diffusion states for the RUVBL1^Halo^ protein, ranging from an immobile population to a freely diffusing one (Figure 3b).^38^ Upon treatment with CB-6644, we observed a transition from the slower state to the faster state and hypothesized that this represents a decrease in the fraction of RUVBL proteins which are associated with various macromolecular complexes. Unlike the ADP-Glo assay, the SMT assay provides robust and highly reproducible measurement of compound potency (Figure S2a). Furthermore, we found that data from our SMT assay exhibited a high correlation with the cell viability and in vitro MYC degradation assays (Figures 3 and S3b). It is noted that the *Z*′ values for the RUVBL SMT assay across runs are not over 0.5 (Figure S2b), likely because this cellular assay shows a ∼60% dynamic window between the active and negative controls. However, reproducible EC_50_ measurements between runs and tight correlations with the cell viability and MYC degradation assays have been observed, increasing confidence in driving the SAR campaign using RUVBL1/2 protein dynamics assessed with the htSMT technology.

**Figure 3:**
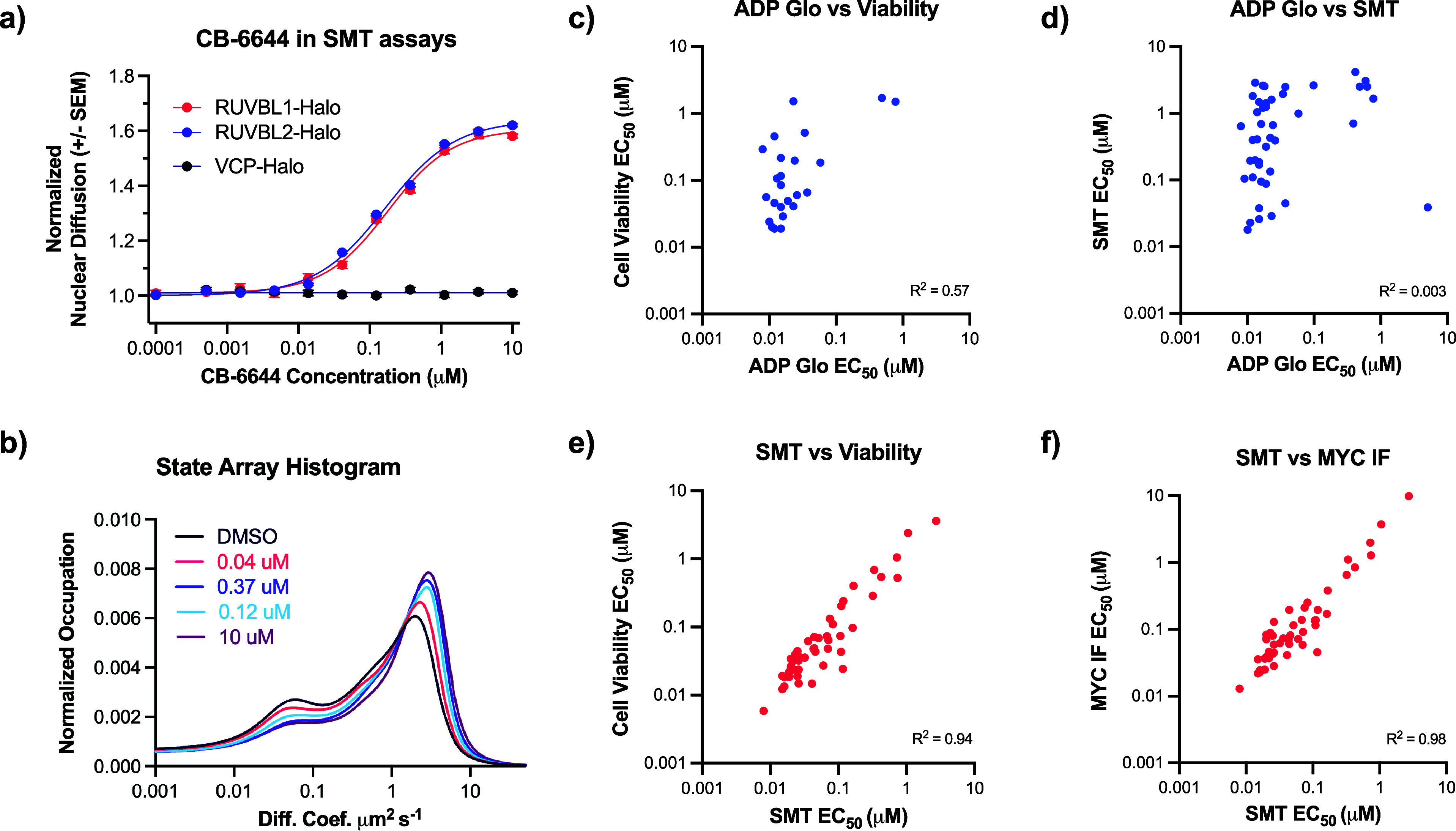
Cellular RUVBL1/2 SMT assays have higher correlations to viability and MYC degradation assays than the ADP-Glo assay. (a) Upon treatment with CB-6644, a dose-dependent increase in the nuclear diffusion of RUVBL1 or RUVBL2 proteins are observed respectively in Halo-tagged cells, with the diffusion ratio representing the ratio of mean diffusion coefficients derived from the full diffusion coefficient distributions at each concentration; no diffusion change observed for VCP-Halo cells. (b) A state-array histogram showing the distribution of diffusion states for the RUVBL1-Halo protein. (c) The ADP-Glo assay (*x*-axis) shows poor correlation with the cell viability (*R* ^2^ = 0.57) and (d) RUVBL SMT assays (*R* ^2^ = 0.003). (e) In contrast, the RUVBL SMT assay (*x*-axis) shows improved correlation with the cell viability (*R* ^2^ = 0.94) and (f) correlates strongly with in vitro MYC degradation assay results (*R* ^2^ = 0.98).

Global proteomic profiling via Tandem Mass Tags (TMT) mass-spectrometry of HCT116 cells (Figure 4) respectively treated with compound 1 (Figure 5) or vehicle for 8, 24, or 48 h revealed that reduced MYC expression was a primary response of RUVBL inhibition. Comparatively, protein abundance for other short-lived proteins (*t*_1/2__≤_ 0.5 h) such as PRELID3B, NFE2L1, and CCND1 ^40^ was unchanged or upregulated versus DMSO controls, suggesting that the markedly reduced MYC protein abundance observed was specific and unlikely an off-target effect of generally diminished protein homeostasis of short-lived proteins. The observed reduction in MYC protein expression was consistent with lower detection of MYC observed with CB-6644 treatment in HCT116 and in the reported immunoblots of other RUVBL1/2 inhibited cancer cell-line models (Figure S3). These findings support the use of nuclear MYC level as a PD biomarker for RUVBL inhibition.

**Figure 4:**
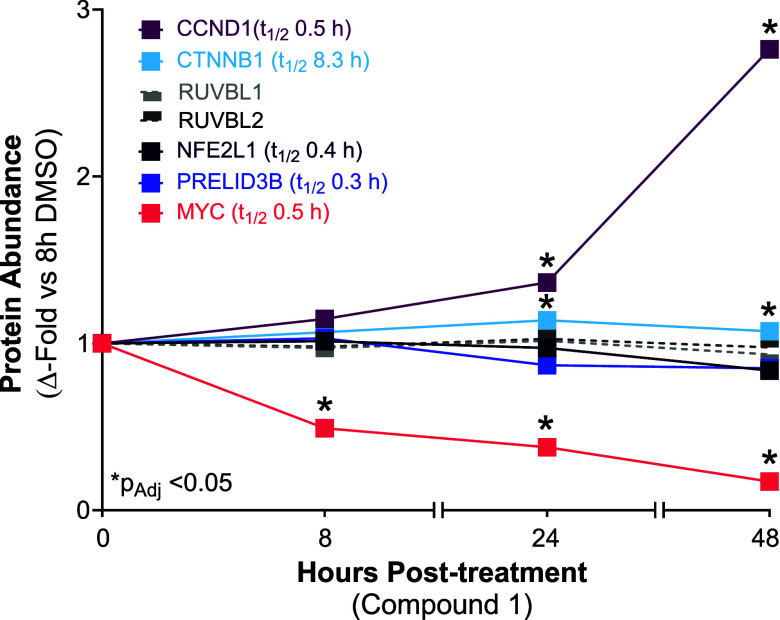
Downregulation of MYC protein is observed with the inhibition of AAA ATPases RUVBL1 and RUVBL2. MYC is significantly reduced in respectively treated HCT116 samples as measured by TMT mass-spectrometry, compared to other short-lived proteins (*t*_1/2_ < 0.5 h) such as PRELID3B, NFE2L1, and CCND1^(40)^ at 8, 24, and 48 h post-treatment with compound 1 (1 μM). Additionally, protein levels of CTNNB1, RUVBL1 and RUVBL2 are relatively unchanged for the entire durations of compound 1 treatment.

**Figure 5:**
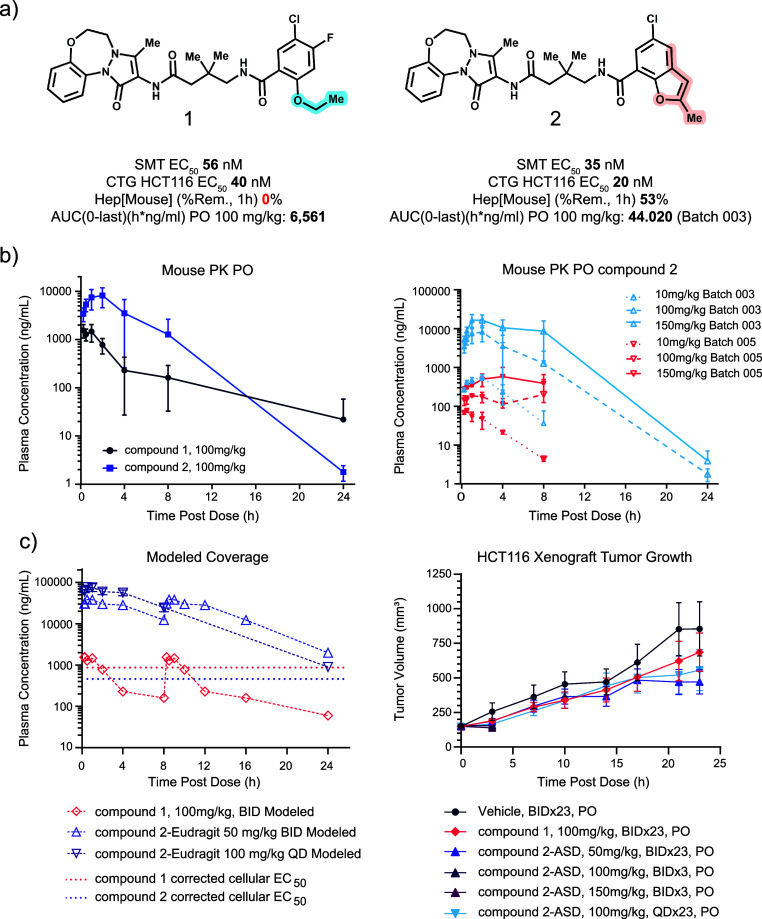
Compound 1 and 2 in vitro and in vivo profile. (a) Structures and profiles of compound 1 and 2; (b) mouse PK results of compound 1, 2, and the ASD forms of 2 (*n* = 3 per group); (c) compound 1 and 2 modeled coverage over EC50 corrected by PPB (left) and the antitumor activities in the HCT 116 mouse xenograft (*n* = 8 per group, right).

Using CB-6644 as the starting point, a new RUVBL inhibitor, compound 1, was designed by removing the methoxymethyl group to reduce the molecular weight and introducing an oxygen atom in the tricycle to reduce the lipophilicity. The in vivo studies of compound 1 were conducted using the HCT116 mouse xenograft model, where in vivo target engagement had been observed using nuclear MYC level as PD biomarker, together with the modest TGI observed in the efficacy study (Figure S4). The modest TGI could be due to the low metabolic stability (0% remaining after 1 h in mouse hepatocytes) and suboptimal PK exposure of compound 1. The ethoxy group on compound 1, a metabolic hotspot, could be responsible for its poor metabolic stability. Cyclization to afford a benzofuran could increase stability. To our delight, benzofuran analog compound 2 not only showed improvement in the hepatocyte stability in vitro and had improved mouse PK exposure, it was also more potent in the SMT and viability assays than compound 1 (Figure 5a). However, the large-scale batch 005 exhibited much lower exposure compared to its original batch (batch 003) in the mouse PK experiment (Figure 5b). X-ray powder diffraction (XRPD) revealed that the two batches are different crystalline forms. Multiple attempts to convert the batch 005 material to a crystalline form matching the original batch 003 were unsuccessful. Instead, two amorphous solid dispersions (ASD) were developed to boost solubility and PK exposure. Both ASD samples showed much higher solubility in simulated intestinal fluid, and higher exposure in mouse PK experiments than both 005 and 003 batches (Figure S5). PK modeling suggested that compound 1 could only achieve 4 h coverage over its in vitro EC_50_ at 100 mg/kg BID dose, whereas compound 2 ASD sample could achieve 24 h coverage at 50 mg/kg BID dose or 100 mg/kg QD dose. Notably, compound 2 ASD sample demonstrated much higher coverage profile, providing >16 h coverage over 10 times of the in vitro EC_50_. Compound 1 and the EUDRAGIT L100 ASD sample of compound 2 were tested in the HCT116 mouse xenograft model. Body weight loss was observed after 3 days for mice dosed at 100 mg/kg and 150 mg/kg BID of compound 2, indicating poor tolerability at high doses. Compared to the 25% TGI observed for compound 1 at 100 mg/kg BID dose, 54% and 42% TGI were observed for compound 2 at 50 mg/kg BID and 100 mg/kg QD groups respectively (Figure 5c). It was encouraging to see that the increase of coverage led to improved antitumor activity, but the observed narrow therapeutic window (less than 2-fold from nontolerated doses) was noted. The team aimed to develop more structurally distinct RUVBL inhibitors for in vivo evaluation to understand whether the narrow therapeutic window is compound specific.

An extensive SAR campaign around compound 2 was conducted utilizing the RUVBL SMT assay. To improve the compound’s physicochemical properties, the team explored introducing polar functionalities across the molecular structure. A pyridyl replacement of phenyl group on the left-hand vector resulted in a less potent compound 3 with significantly increased Caco-2 efflux ratio (Table 1). Experiments with *P*-glycoprotein (P-gp) inhibitors confirmed that the efflux observed in this series is mediated by P-gp. Adding a nitrogen atom to the right-hand benzofuran gave compound 4, which exhibited good potency but high efflux. Compound 5, where a methyl group was replaced with a hydroxy group, also suffered from high efflux. A similar trend of increasing efflux with the increase of the number of hydrogen-bond donors and acceptors has been observed for other analogs. Notably, these more polar analogs did not show an increase in metabolic stability, and they exhibited lower exposure in the mouse PK experiments. This shifted our focus from introducing polarity to investigating how to navigate the efflux issue. Compound 6, a regioisomer of compound 2, showed reduced potency and unexpectedly exhibited a significantly decreased efflux ratio in the Caco-2 assay. The results suggested that the left-hand tricycle is the main contributor of the P-gp mediated efflux. Modification of the left-hand tricycle to remove one hydrogen-bond acceptor yielded compound 7, which exhibited lower efflux but compromised potency. Surprisingly, the des-methyl analog compound 8 had an efflux ratio of only 1.5. While efflux mediated by P-gp is generally linked to the number of hydrogen-bond donors and acceptors, our results highlight two unique cases, where conformational change or simple methyl removal substantially reduced efflux. Despite the promising permeability profile of compounds 7 and 8, the potency loss and metabolic instability hindered these compounds from moving forward.

**Table 1:**
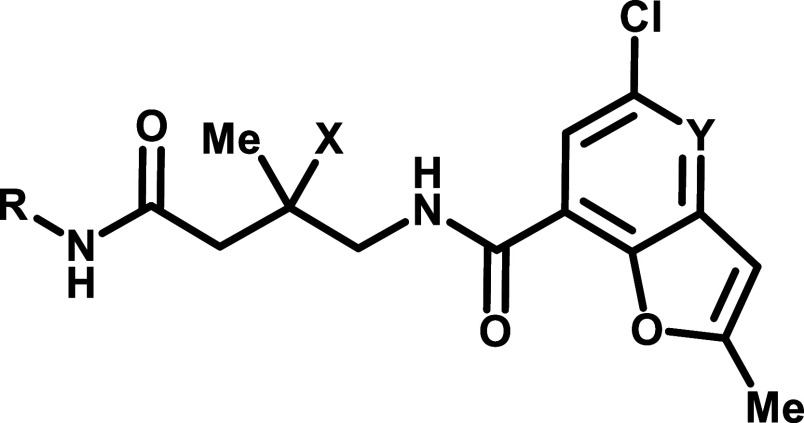

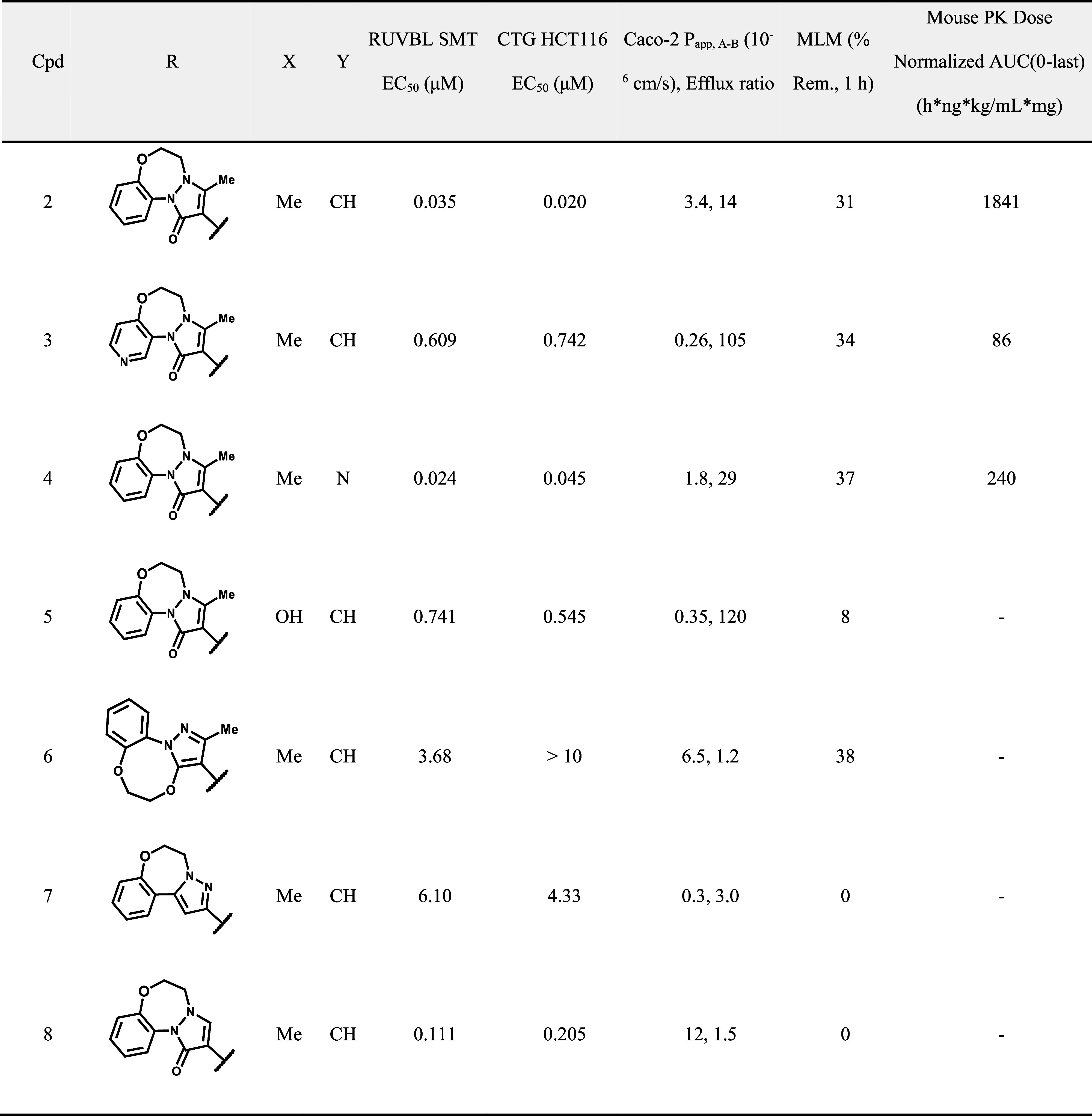
SAR Exploration around Compound 2

Since early attempts on the systematic introduction of polar functionalities did not significantly improve the metabolic stability (compounds 3–5), the metabolite identification (Met ID) of compound 2 in mouse hepatocytes was conducted. The Met ID revealed two predominant sites of metabolism, oxidation on the substituted benzofuran, and the hydrolysis of the right-hand amide group (Scheme 1). To probe whether the furan functionality of the benzofuran ring was responsible for the oxidative metabolism, benzoxazole compound 9 was prepared. However, no improvement in stability was observed. In addition, compound 9 exhibited a loss in potency and increased efflux consistent with previous polar analogs (Table 2). Interestingly, des-methyl compound 10 showed remarkable stability increase in mouse microsomes and hepatocytes, suggesting that the methyl group was a metabolic hotspot. Despite improvement in the metabolic stability, compound 10 suffered from potency loss, indicating the necessity of the methyl group for activity. Other replacements of the methyl group to block the metabolic site, such as the difluoromethyl compound 11, did not yield acceptable potency and metabolic stability in the same molecules. Replacement of the right-hand amide group with bioisosteres to alleviate the hydrolysis liability, afforded molecules including oxadiazole 12. Unfortunately, the replacements of the amide group failed to maintain the activity. Reversing the amide conformation to give compound 13 maintained some activity but still suffered from over 20-fold potency loss. These findings highlighted the importance of the amide configuration and suggested that the amide NH might serve as a key contributor to the observed activity.

**Scheme 1:**
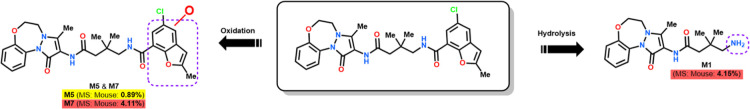
Metabolic Profiling of Compound 2

**Table 2:**
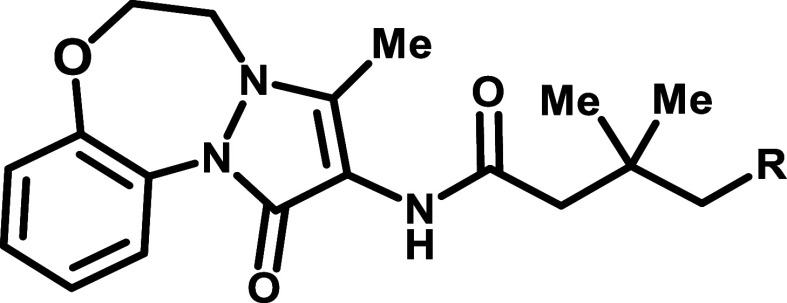

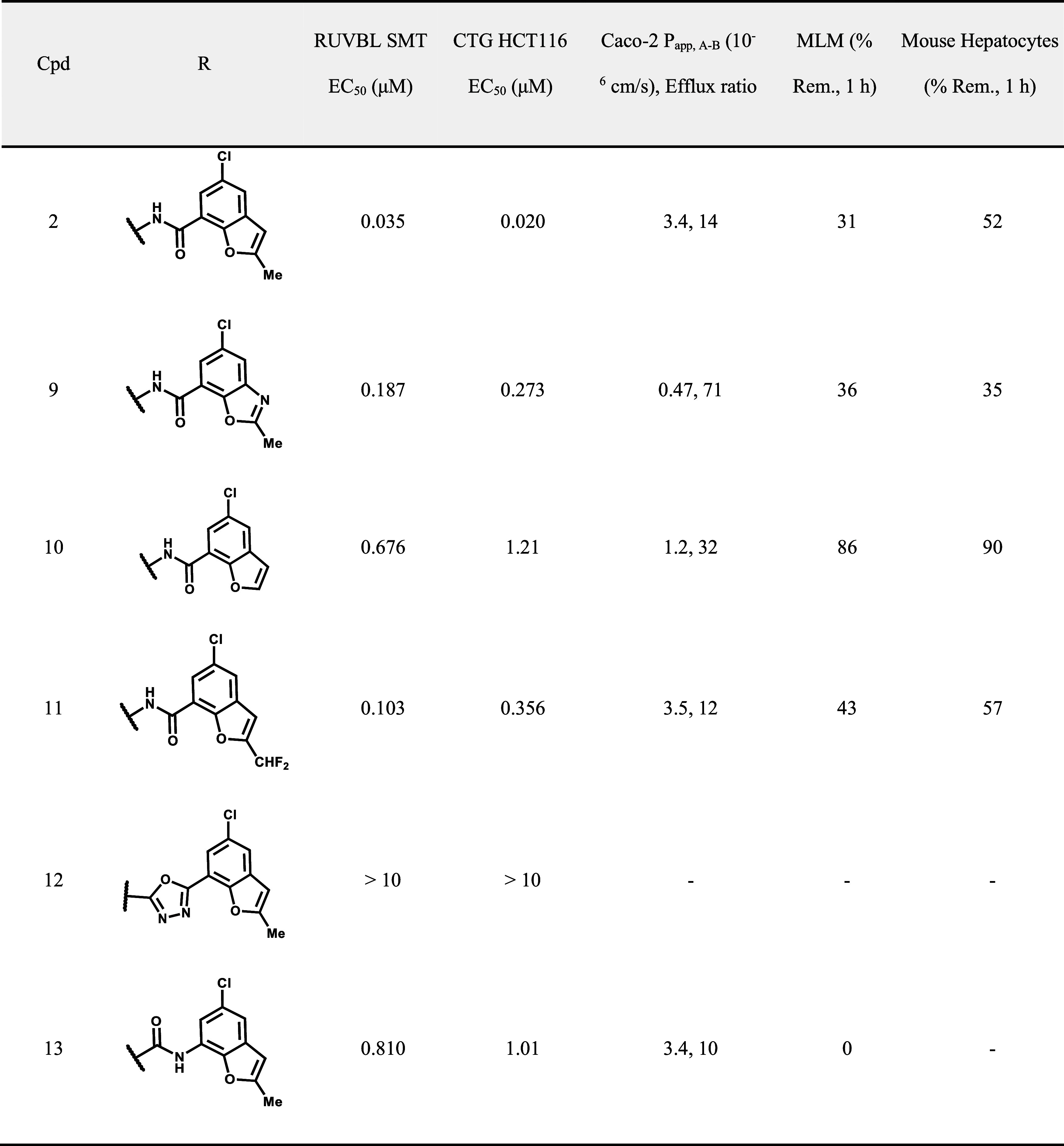
SAR Exploration around the Right-Hand Vector

In the absence of a cocrystal or Cryo-EM structure at the time the SAR studies were conducted, it was difficult to estimate how the inhibitor might bind to the protein and whether the amide nitrogen is forming a key interaction with the protein. Some insight regarding the 3D conformation of compound 1, however could be gathered by comparing the EPSA^41,42^ and topological polar surface area (tPSA) values. EPSA, which is not an acronym, can be deemed as representing experimental or exposed polar surface area. The parameter, measured via supercritical fluid chromatography, provides insight into the exposed polarity and impact of intramolecular bonding on molecule conformation. By contrast, tPSA, which is a fragment based 2D surrogate for 3D polar surface area, does not explicitly account for solvation or conformational changes. A composite parameter, e.g. (tPSA—EPSA), can be informative in determining whether a compound can form a stable structure established through intramolecular bonding e.g. intramolecular hydrogen-bonding (IMHB). For compound 1 the (tPSA—EPSA) difference is substantial, suggesting the compound’s ability to form a stable structure through IMHB (Figure 6a). Based on this insight, we could hypothesize two plausible conformations for compound 1. The NH from the right-hand amide forms a 6-membered IMHB with the ether oxygen, which is also seen in the single crystal X-ray diffraction of compound 1. However, the cyclization on the right-hand vector to mimic this IMHB showed no activity in the SMT and cell growth assays (Figure S6). Computational energy minimization of compound 1 suggested a 7-membered IMHB between the right-hand NH and the left-hand amide carbonyl. Inspired by this, a library of different cyclic linkers replacing the dimethyl butanoate group in compound 2 was screened, and interesting results were obtained with the bridged bicyclic analogs. While the [2.1.1] bicyclic compound 14 showed no activities, its [3.1.1] homologue showed moderate activities in both cellular assays (compound 15). The [4.1.1] bicyclic compound 16 exhibited 22 nM EC_50_ in the SMT assay and 23 nM EC_50_ in the HCT116 cell growth assay (Table 3). The results suggested that the angle of the bicyclic linker plays an important role in the protein–ligand interaction by rigidifying the compounds in a preferred conformation. During our SAR campaign, a Cryo-EM structure of RUVBL1/2 complex and CB-6644 was published.^13^ Molecular modeling of compound 16 and CB-6644 with RUVBL1/2 complex indicated that the [4.1.1] bicyclic linker effectively mimics the active conformation of CB-6644 and anchors the left-hand and right-hand vectors into corresponding hydrophobic pockets. Other than potency benefits, the bicyclic linkers also improved the metabolic stability, probably due to the steric hindrance around the amide group, resulting in reduced hydrolysis. Furthermore, [4.1.1] bicyclic compound 16 also showed a decreased efflux ratio.

**Figure 6:**
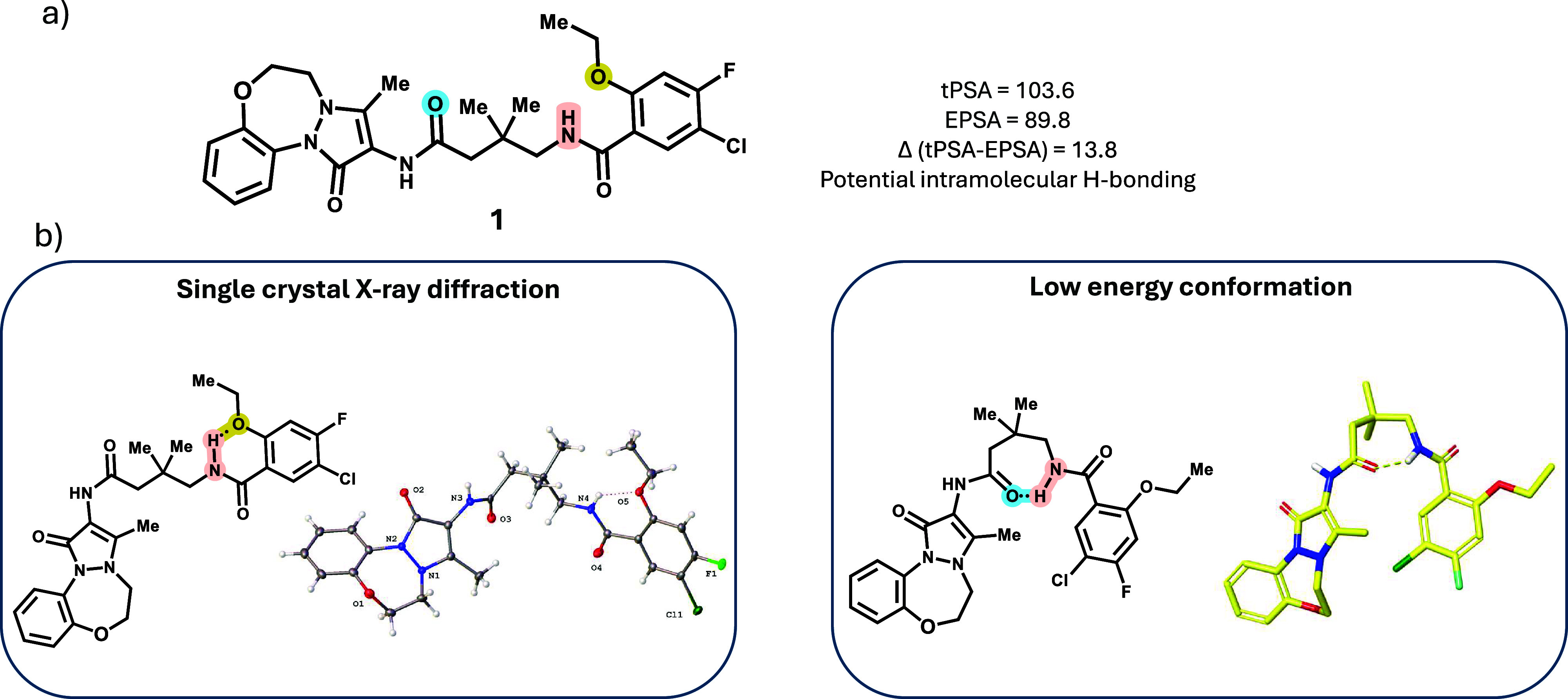
(a) Difference between tPSA and EPSA of compound 1 suggests potential intramolecular H-bonding (IMHB); (b) possible IMHB modes suggested by single crystal X-ray diffraction (left) and low energy modeling (right).

**Table 3:**
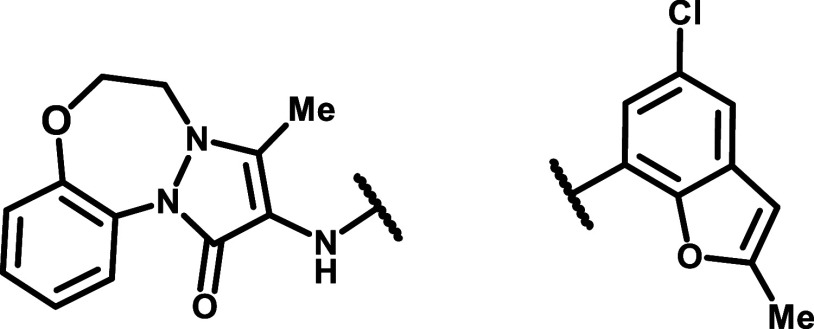

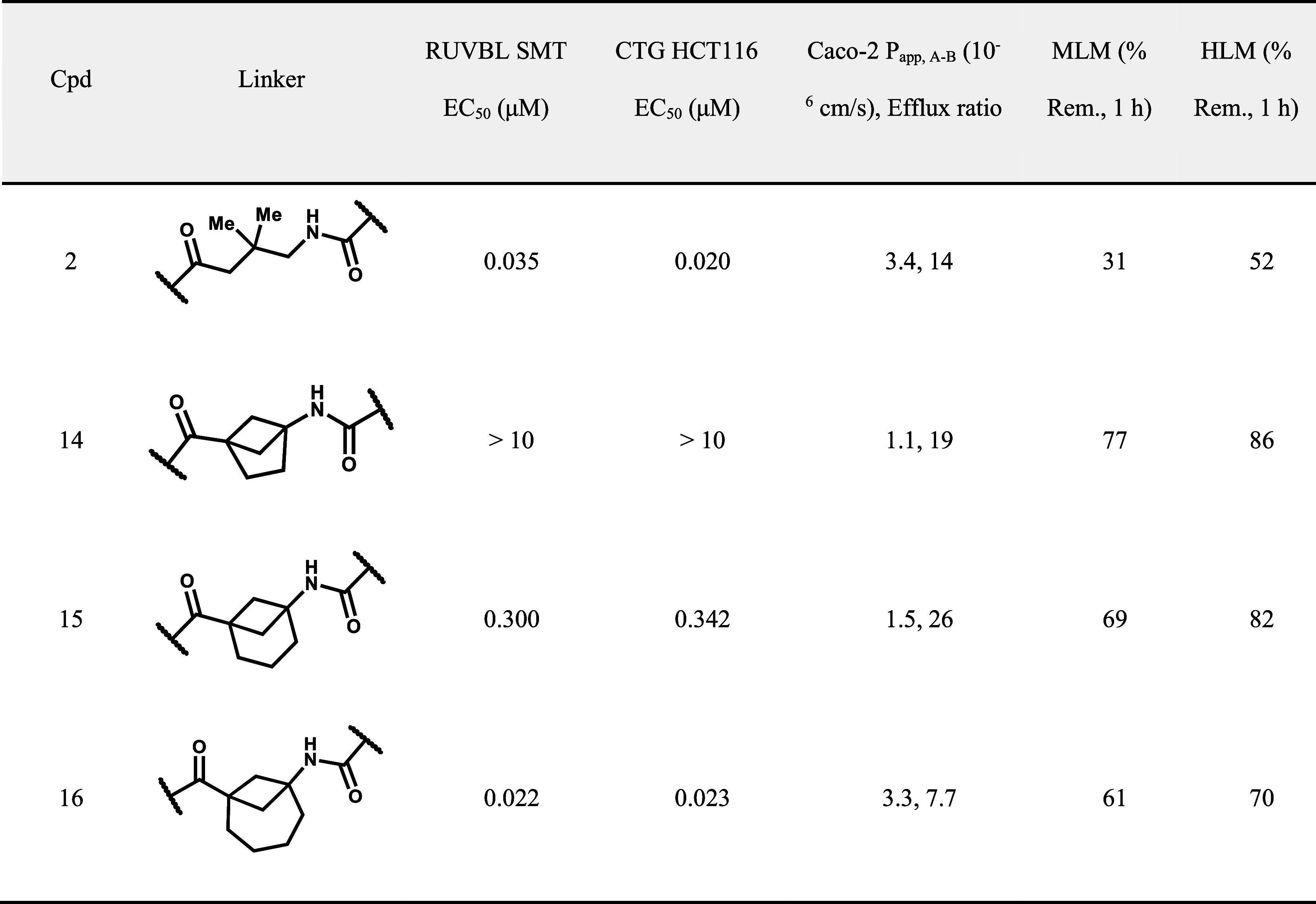
SAR Exploration around Cyclic Linker

Installation of the [4.1.1] bicyclic linker also enabled other opportunities for compound development (Table 4). Breaking the 7-membered ring on the tricycle maintained potency and further decreased efflux ratio to 2.4 (compound 17). However, compounds 16 and 17 showed suboptimal kinetic solubility, probably due to the increased hydrophobicity introduced by the [4.1.1] bicyclic group. Therefore, we revisited our strategy to increase solubility by installing polar functionalities. Early attempts with a noncyclic linker resulted in efflux issues and poor PK exposure (Table 1). Previous SAR had demonstrated that the position of the nitrogen on the benzofuran ring matters for potency. Compound 4 with the nitrogen at the 4-position of the 5,6-fused ring system was >7-fold more potent than the isomer compound 9, where the nitrogen is located at the 3-position. By looking at the binding mode of how our molecule docks in the RUVBL1/2 protein, we found that the right-hand vector of the molecule sits into a hydrophobic pocket of the RUVBL1/2 complex, where no proximal polar residues are available for hydrogen bonding interactions (Figure 7). This encouraged us to introduce nitrogen atoms at positions that are “buried” within the bicyclic ring system, or located adjacent to the chlorine substituent, to avoid potency loss by having unfavorable protein interactions in the hydrophobic pocket. Finally, the introduction of a more polar imidazopyridazine scaffold in place of the benzofuran ring resulted in compound 18, demonstrating gains in potency, solubility, and metabolic stability. Furthermore, compound 18 exhibited no significant efflux liability compared with early analogs that have introduced polar functionalities. This observation highlights the advantages of switching to the [4.1.1] bicyclic linker. This successful introduction of additional polarity reduced the cLogP without compromising potency, leading to an overall improvement in lipophilic efficiency (LipE) and resulted in significantly higher free fractions in the plasma protein binding assays. Compared to the reference inhibitor CB-6644, compound 18 demonstrated broad improvements across multiple properties, including cellular potency, solubility, permeability, and metabolic stability. Additionally, counter-screening in CB-6644-resistant HCT116-RUVBL1^R117H^ mutant indicated comparable fold-changes in reduced potency in the mutant vs parental wild-type (Figure S9).

**Table 4:**
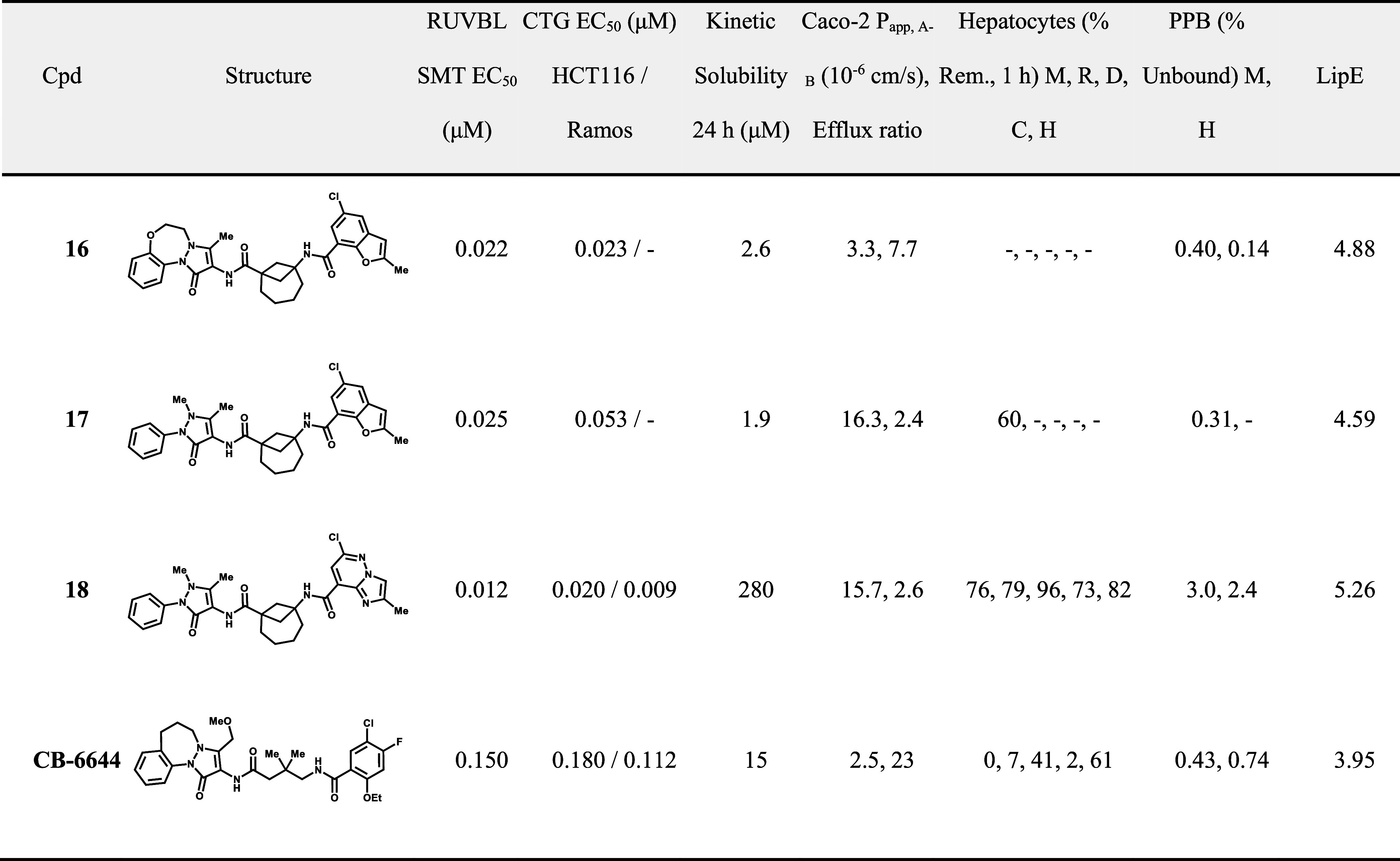
Final Development of [4.1.1] Bicyclic Analogs and the Comparison with CB-6644^a^

a LipE is calculated from RUVBL SMT EC_50_ and ChromLogD.

**Figure 7:**
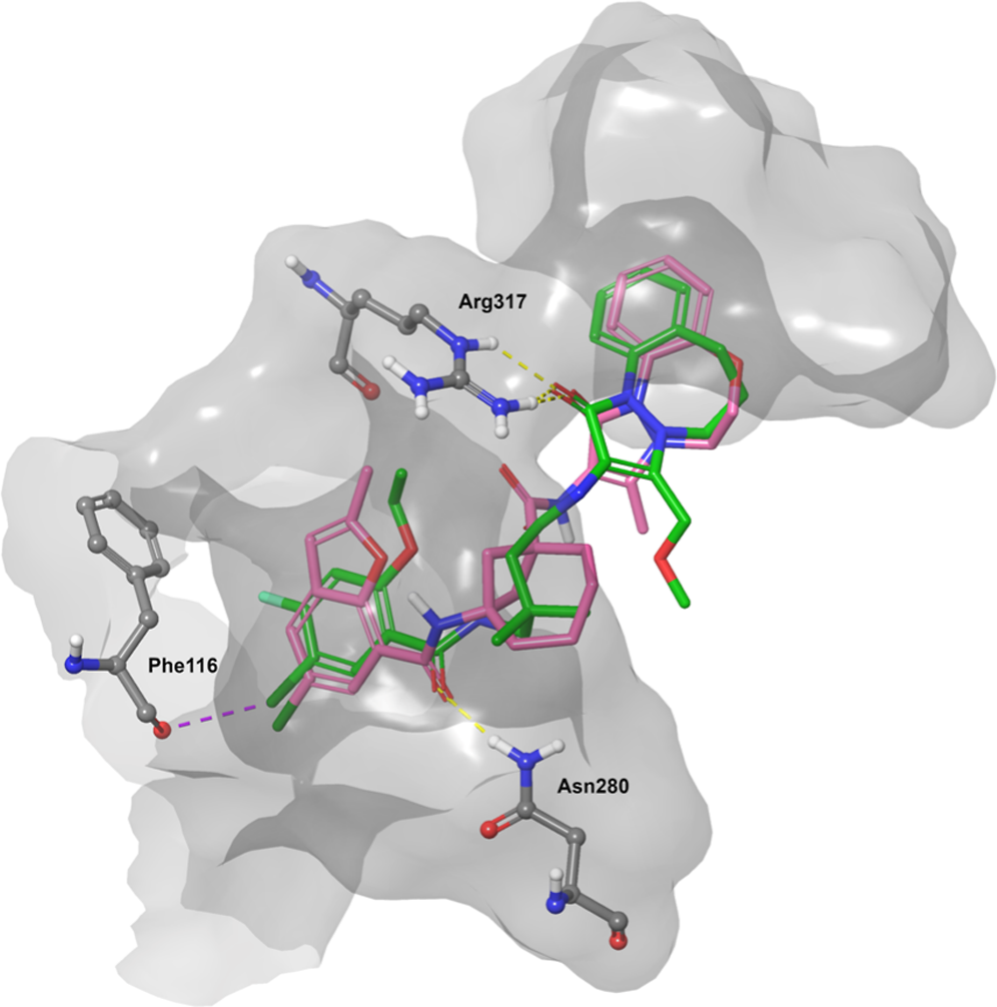
Compound 16 (pink) was docked into the Cyro-EM structure of PDB entry 9EMA, proposing a similar binding mode as CB-6644 (green). Key hydrogen bonds to Arg317 and Asn280 are shown.

The improved potency and physicochemical properties of compound 18 translated into favorable mouse PK parameters and robust target coverage projections in PK modeling. Whereas a 150 mg/kg BID dose of CB-6644 maintained target coverage above the cellular EC_50_ for approximately 6 h, compound 18 demonstrated superior exposure at 10 mg/kg BID dose with around 16 h coverage of EC_50_ (Figure 8a). Higher doses also allow 24 h coverage of EC_50_. Compound 18 was subsequently evaluated for in vivo efficacy in the Ramos xenograft model of MYC-dependent Burkitt Lymphoma.^43–45^ Consistent with published results, the reference inhibitor CB-6644 showed 70% tumor growth inhibition (TGI) at a 150 mg/kg BID dose.^11^ As anticipated from the improved mouse PK, compound 18 achieved 84% TGI at a 10 mg/kg BID dose, with no body weight loss observed (Figure 8b). However, signs of intolerability emerged at higher doses starting at 30 mg/kg BID. This finding indicated a relatively narrow therapeutic window, consistent with the results from compound 2 in the HCT116 xenograft.

**Figure 8:**
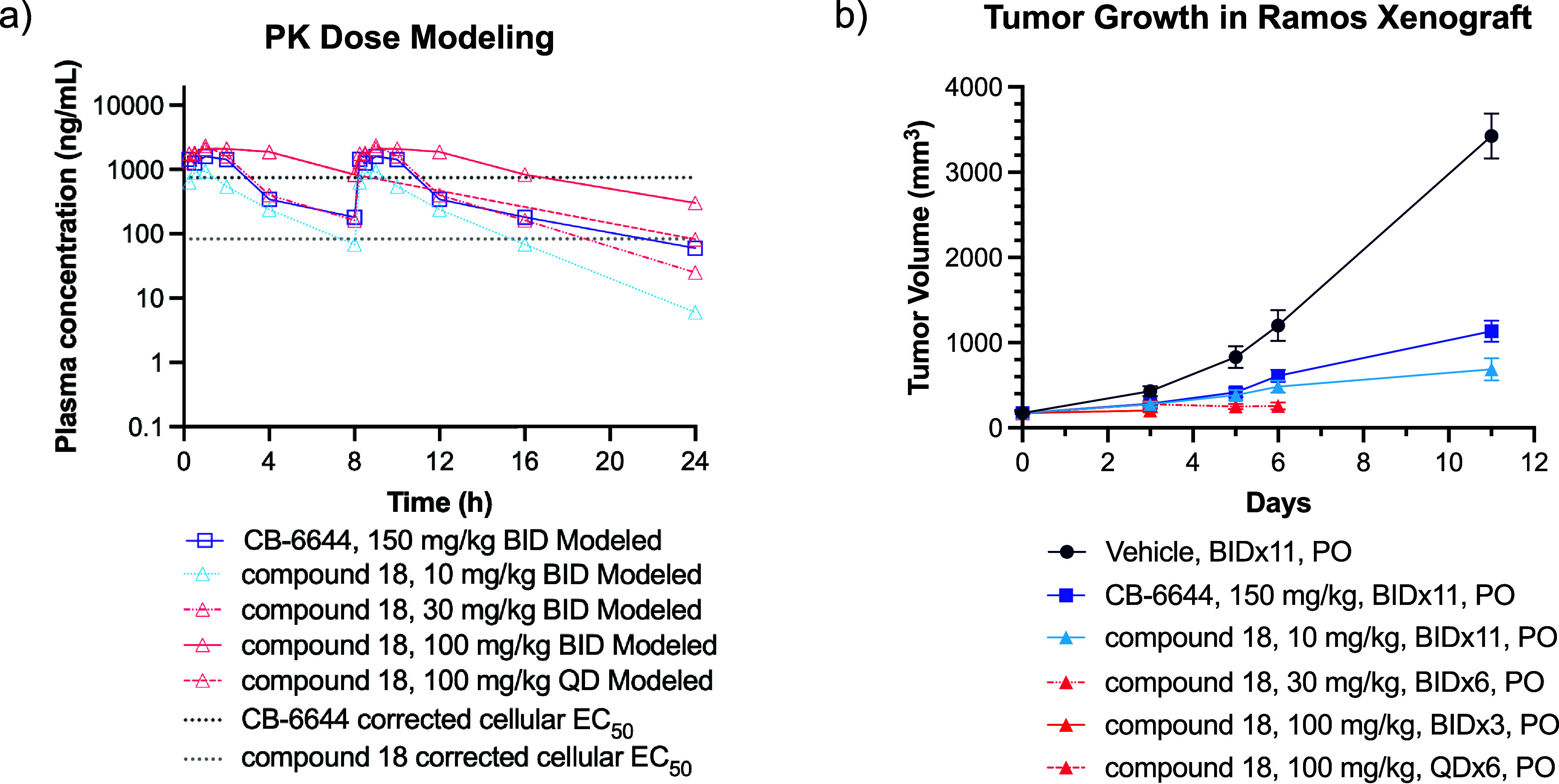
(a) Compound 18 and CB-6644 modeled coverage over viability EC_50_ corrected by PPB. (b) The antitumor activities in the Ramos mouse xenograft. Doses of compound 18 in pink were not tolerated.

To ensure that the improved potency and efficacy were not due to off-target effects, we evaluated RUVBL engagement by compound 18 in multiple in vitro assessments to characterize the on-target specificity and on-pathway effects, relative to the well-characterized effects of the direct-binder, CB-6644. Generation of RUVBL1 point mutants previously reported in CB-6644-resistant HCT116 cells, were recurrently identified in HCT116 cells exposed to increasing concentrations of CB-6644.^11^ Genomic sequencing (Figure S8) confirmed the recurrence of the RUVBL1^R117H^ mutation and were subsequently assessed, alongside parental controls, for antiproliferative effects in comparing compound 18 to CB-6644 (Figure S9b–d). A similar potency shift from WT to R117H mutants was observed between compound 18 and CB-6644, with the EC_50_ increasing >20-fold for both RUVBL inhibitors evaluated. Secondarily, both RUVBL inhibitors demonstrated single-digit micromolar to nanomolar potency, demonstrating that diminished on-target effects could result in observed viability effects in the in vitro setting. To evaluate compound 18’s more proximal on-target effects than viability or the reduced expression of MYC observed with RUVBL inhibition, we assessed whether compound 18’s effects on the DNA damage response by the phosphatidylinositol 3-kinase-related kinases (PIKKs) pathway would similarly be inhibited. We observed that etoposide-induced upregulation of phospho-CHK2 (*p*-CHK2), was comparably inhibited by both CB-6644 and compound 18 when coadministered in HCT116 (Figure S10). Notably though, compound 18 demonstrated greater potency by achieving the comparably reduced *p*-CHK2 levels at 40% of the CB-6644 dose administered for the 1 h treatments (500 nM vs 200 nM, respectively). Impairment of the DNA damage response, as observed with reduced *p*-CHK2 levels, further supports compound 18’s on-target effects noting RUVBL1/2’s role in the PIKK axis response to etoposide.^12,13^ These findings suggest that the observed antiproliferation effects for compound 18 can be attributed to the on-target RUVBL inhibition, and that the enhanced in vitro and in vivo activities compared to CB-6644 are not due to off-target cytotoxicity.

To further assess a MYC-dependent hypothesis of RUVBL inhibition, a mini-panel of MYC^Hi^ and MYC^Lo^ cell-lines was selected for treatment with RUVBL inhibitors based on MYC expression levels quantified by Western blotting (Figure S11a,b). Subsequently, viability assessments demonstrated that all 3 RUVBL inhibitors exhibited similar average EC_50_s across both the MYC^Hi^ and MYC^Lo^ cell-panels, though compound 18 exhibited greater potency than both CB-6644 and compound 1 (Figure S11c). These results demonstrate that RUVBL inhibition in the in vitro setting is potent despite differential levels of MYC expression, which supports the understanding of RUVBL’s diverse cellular functions outside of MYC transcriptional regulation. Despite the limited tolerability observed in the xenograft model and the lack of MYC-dependency in the sensitivity to RUVBL inhibitors, these immuno-compromised models and in vitro experiments exclude any additional effects from inhibiting MYC’s role in tumor evasion.^17–20^ We believe that compound 18’s improved potency and DMPK profile yielded a tool compound more capable of determining the therapeutic potential of RUVBL inhibition in future studies such as its activity in immune-competent animal models.

## 3 Chemistry

Compounds 1 and 2 were synthesized via a common synthetic route beginning with the construction of the tricyclic core as outlined in Scheme 2. Reaction of bromoacetaldehyde diethyl acetal with 2-bromophenol 19 afforded intermediate 20, which upon acetal deprotection with aqueous HCl yielded aldehyde 21. Reductive amination of 21 with 5-methylpyrazolidin-3-one provided the *N*-linked intermediate 22. An intramolecular copper-mediated cyclization of 22 constructed the middle 7-member oxadiazepine ring in the tricyclic core of intermediate 23. α-Bromination of 23 using *N*-bromosuccinimide, followed by base-induced elimination, furnished pyrazolone intermediate 24. Subsequent nitration of 24 with nitric acid yielded the corresponding nitro derivative, which was reduced using iron powder under mildly acidic conditions to afford amine intermediate 25.

**Scheme 2:**
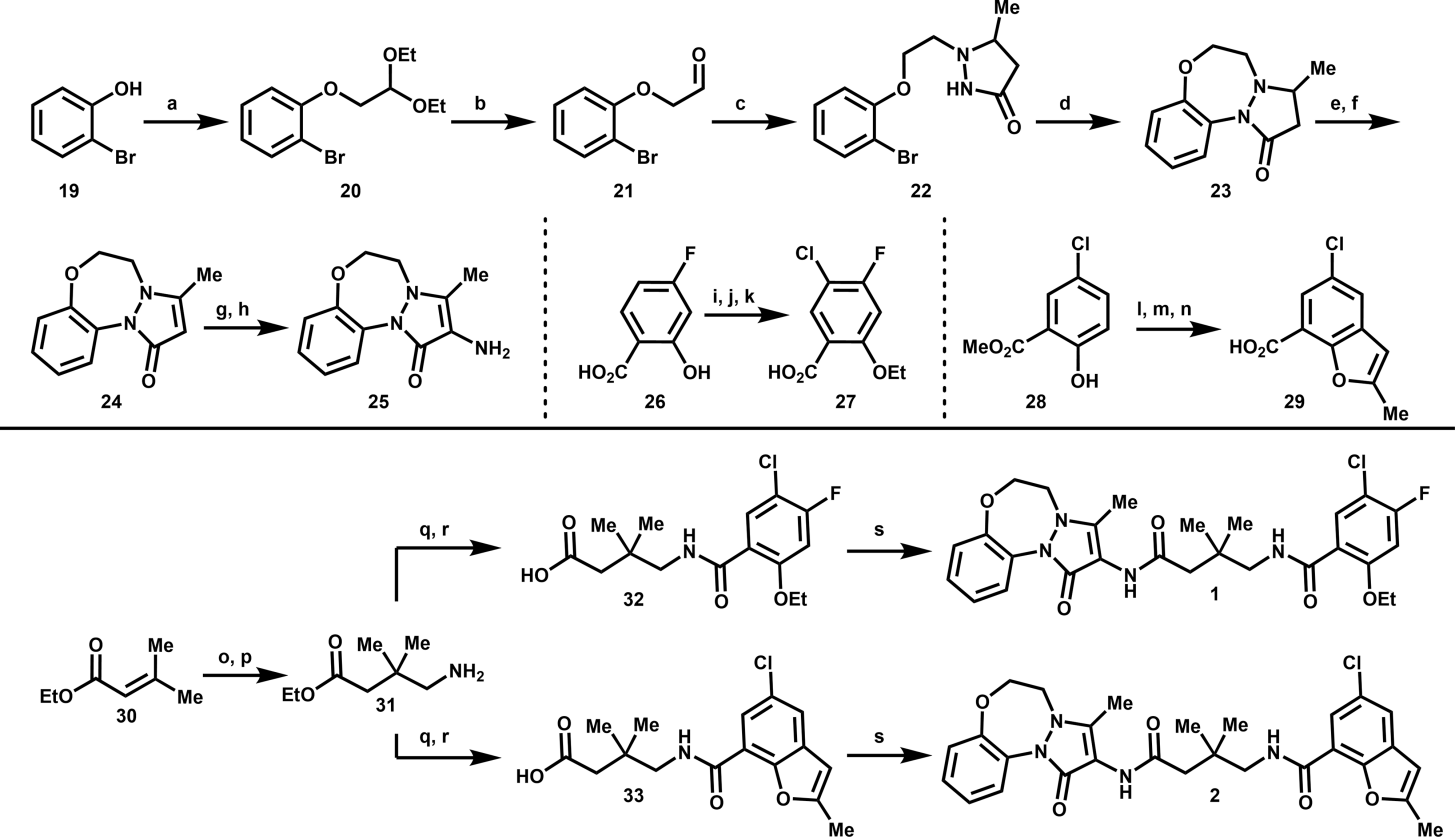
Synthesis of Compounds 1 and 2^a^

a Reagents and conditions: (a) KOH in H_2_O, rt, then 2-bromo-1,1-diethoxyethane in DMSO, rt to 100 °C, 96%; (b) conc HCl, 1,4-dioxane, H_2_O, rt, 82%; (c) 5-methylpyrazolidin-3-one, anhydrous MeOH, rt, then NaBH_4_, 0 °C to rt, 58%; (d) CuI, 1,10-phenanthroline, Cs_2_CO_3_, anhydrous 1,4-dioxane, rt, 49%; (e) LHMDS, THF, −78 °C, then NBS in THF, −78 °C; (f) DBU, 1,4-dioxane, 80 °C, 54% over two steps; (g) TFA, HNO_3_, 0 °C to rt; (h) Fe, NH_4_Cl in EtOH/H_2_O, DCE, rt to 80 °C, 72% over two steps; (i) sulfuryl chloride in CH_2_Cl_2_ dropwise, rt, 82%; (j) iodoethane, K_2_CO_3_, DMF, rt to 70 °C, 57%; (k) LiOH·H_2_O, THF, H_2_O, rt, 89%; (l) propargyl bromide, K_2_CO_3_, MeCN, 80 °C, 65%; (m) CsF, *N*,*N*-diethylanaline, 200 °C, 45%; (n) LiOH·H_2_O, EtOH, H_2_O, rt, 64%; (o) nitromethane, DBU, MeCN, 80 °C, 91%; (p) 10% Pd/C, H_2_, EtOH, 50 °C, 79%; (q) 27 or 29, HATU, TEA, THF, rt, then 31, TEA, rt, 48% and 40% respectively; (r) LiOH·H_2_O, THF, H_2_O, rt, 67% and 49% respectively; (s) HATU, DIPEA, DMSO, rt, then 25, 19% and 16% respectively.

Synthesis of the right-hand vectors of compounds 1 and 2 follow two distinct routes. For compound 1, chlorination of commercially available 4-fluoro-2-hydroxybenzoic acid 26 with sulfuryl chloride was followed by alkylation with iodoethane. Finally, saponification using lithium hydroxide furnished carboxylic acid 27. For compound 2, alkylation of commercially available methyl 5-chloro-2-hydroxybenzoate 28 with propargyl bromide was followed by a 3,3-sigmatropic Claisen rearrangement and subsequent cyclization to form the benzofuran fragment. Saponification of this intermediate afforded carboxylic acid 29.

Final assembly of compounds 1 and 2 began with a base-catalyzed 1,4-Michael addition between ethyl 3-methylbut-2-enoate 30 and nitromethane, followed by Pd/C-mediated reduction to yield amine 31. At this stage, the synthesis split into two parallel pathways. Activation of carboxylic acid 27 or 29 with HATU followed by coupling with amine 31 afforded the corresponding ester intermediates, which were hydrolyzed to provide acids 32 and 33, respectively. A second HATU-mediated coupling with amine 25 yielded compounds 1 and 2.

Synthesis of compound 18, outlined in Scheme 3, starts with HATU activation of commercially available Boc protected [4.1.1] bridged bicyclic amino acid 34 and subsequent reaction with commercially available amine, 4-amino-1,5-dimethyl-2-phenyl-1,2-dihydro-3H-pyrazol-3-one. Succeeding HCl catalyzed Boc deprotection afforded amine intermediate 35 as the HCl salt. A final HATU mediated coupling of commercially available 6-chloro-2-methylimidazo­[1,2-*b*]­pyridazine-8-carboxylic acid and amine 35 afforded compound 18.

**Scheme 3:**
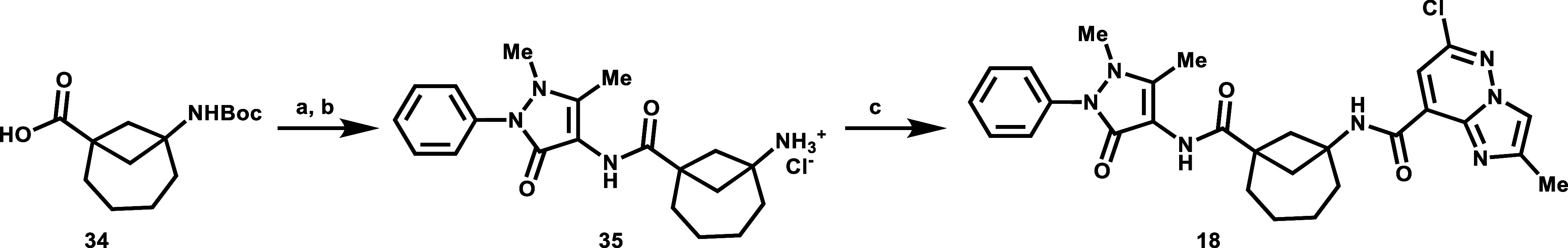
Synthesis of Compound 18^a^

a Reagents and conditions: (a) HATU, DIPEA, CH_2_Cl_2_, rt, then 4-amino-1,5-dimethyl-2-phenyl-1,2-dihydro-3H-pyrazol-3-one, 99%; (b) 4 M HCl in 1,4-dioxane, CH_2_Cl_2_, rt, 99%; (c) 6-chloro-2-methylimidazo­[1,2-*b*]­pyridazine-8-carboxylic acid, HATU, DIPEA, DMSO, rt, then 35, 40%.

## 4 Conclusions

In this study, we set out to evaluate the therapeutic potential of RUVBL inhibition in the treatment of MYC-driven cancers. Previous publications have demonstrated that in vivo efficacy could be achieved with known RUVBL inhibitors. However, in the absence of tool compounds that achieve comprehensive target coverage, the therapeutic window of RUVBL inhibition remained unknown. Given RUVBL’s diverse cellular function and the risks of tolerability issues with RUVBL inhibition, there is a need to develop RUVBL inhibitors with improved potency and DMPK profile. The development of a live-cell, high-throughput Single-Molecule Tracking assay enabled robust and reproducible measurement of compound potency, facilitating a rapid lead optimization campaign. Initial chemotypes suffered from efflux and metabolic instabilities. Cyclization to form a [4.1.1] bicyclic scaffold enhanced activity and physicochemical properties. Further optimization of the left-hand vector and strategic polarity introduction on the right-hand vector yielded compound 18, which exhibited superior potency, DMPK properties, and in vivo efficacy in a Burkitt lymphoma Ramos xenograft model, achieving improved tumor growth inhibition at a 15-fold lower dose relative to the reference inhibitor CB-6644. Together with the increased tumor-growth inhibition in the xenograft studies, a limited therapeutic window was observed, raising potential concerns surrounding the tolerability of RUVBL inhibition. While the RUVBL-specific on-target and on-pathway effects of CB-6644 and compound 18 were confirmed, the lack of an observed MYC-dependent response in the viability assays across MYC-high and MYC-low cell lines supports the role of RUVBL in carrying out diverse cellular functions beyond a role as a MYC cofactor. However, these immuno-compromised models and in vitro experiments exclude any additional effects from inhibiting MYC’s role in tumor evasion.^17–20^ While beyond the scope of these studies, the utilization of compound 18 in immuno-competent tumor models needs to be assessed for any expected enhanced efficacy and tolerability compared to studies utilizing known inhibitors.^16^ Additionally, while a recurrent drug-resistant RUVBL1^R117H^ mutation observed with CB-6644 treatment resulted in reduced potency for compound 18 (Figure S9b–d), the identity of other recurrent or novel drug-induced mutations conferring resistance to compound 18 was not assessed and needs further investigation to ascertain whether resistance-conferring mutations similarly map to those observed for earlier generation RUVBL1/2 inhibitors.^11^

Overall, these findings further demonstrate the utility of SMT as a powerful tool to facilitate drug discovery campaigns, particularly when traditional biochemical assays prove challenging.^38,39,46,47^ The use of a live-cell assay circumvents the need for protein expression of challenging proteins, like those whose assembly into functional oligomeric states cannot be tightly controlled. The robustness and reproducibility of the SMT assay enabled rapid exploration of SAR and advancement of the series toward better drug-like properties, enabling in vivo exploration of therapeutic activity. Taken together, these novel findings further establish SMT as an enabling technology for drug-hunters to develop therapeutics for challenging targets.

## 5 Experimental Section

### 5.1 Chemistry

All compounds assessed for in vitro and/or in vivo biological activity had a purity of 95% or above as estimated from their ^1^H NMR spectra and their HPLC UV traces. All solvents used were commercially available and of analytical grade. Anhydrous solvents were routinely used for reactions. LC–MS data was collected on an Agilent 1260 LC–MS. ^1^H NMR was performed on Bruker Ascend 400. Data for ^1^H NMR are reported relative to residual solvent as calculated by MestReNova and are reported in the following format: ppm (multiplicity, coupling constant, integration).

### 5.2 Synthesis

#### 5.2.1 1-Bromo-2-(2,2-diethoxyethoxy)­benzene (20)

A solution of KOH (3.89 g, 69.5 mmol) in H_2_O (6.70 mL) was added to 2-bromophenol (19) (10.0 g, 57.8 mmol) at rt. To the solution was added 2-bromo-1,1-diethoxy-ethane (17.1 g, 86.8 mmol) in DMSO (100 mL) at rt. The mixture was heated to 100 °C and stirred for 16 h before it was cooled to rt, poured into H_2_O (200 mL) and extracted with MTBE (80 mL × 3). The combined organic layers were washed with 5% NaOH aqueous solution (80 mL), dried over Na_2_SO_4_, filtered and concentrated in vacuo to afford 16.0 g (96% yield) of 1-bromo-2-(2,2-diethoxyethoxy)­benzene (20) as a colorless oil. ^1^H NMR (400 MHz, CDCl_3_): δ 7.50 (dd, *J* = 1.6, 8.0 Hz, 1H), 7.25–7.19 (m, 1H), 6.88 (dd, *J* = 1.2, 8.4 Hz, 1H), 6.81 (dt, *J* = 1.2, 7.6 Hz, 1H), 4.86 (t, *J* = 5.2 Hz, 1H), 4.03 (d, *J* = 5.2 Hz, 2H), 3.80–3.77 (m, 2H), 3.70–3.66 (m, 2H), 1.24 (t, *J* = 7.2 Hz, 6H).

#### 5.2.2 2-(2-Bromophenoxy)­acetaldehyde (21)

A mixture of 1-bromo-2-(2,2-diethoxyethoxy)­benzene (20) (85.3 g, 294.9 mmol) in 1,4-dioxane (150 mL), H_2_O (150 mL) and conc. HCl (150 mL) was stirred at rt for 16 h. The aqueous phase was extracted with CH_2_Cl_2_ (500 mL × 2). The combined organic layers were washed with saturated NaHCO_3_ (400 mL × 2) and brine (800 mL × 2), dried over Na_2_SO_4_, filtered and concentrated in vacuo to afford 52.0 g (82% yield) of 2-(2-bromophenoxy)­acetaldehyde (21) as a light yellow oil. ^1^H NMR (400 MHz, CDCl_3_): δ 9.91 (t, *J* = 0.9 Hz, 1H), 7.60 (dd, *J* = 7.6 Hz, 1.6 Hz, 1H), 7.57–7.51 (m, 1H), 7.25–7.28 (m, 1H), 6.80 (dd, *J* = 8.4 Hz, 0.9 Hz, 1H), 4.63 (d, *J* = 0.9 Hz, 2H). LC/MS (ESI): *m*/*z* = 214.88 [M + H]^+^.

#### 5.2.3 1-(2-(2-Bromophenoxy)­ethyl)-5-methylpyrazolidin-3-one (22)

A mixture of 5-methylpyrazolidin-3-one (24.3 g, 243.0 mmol) and 2-(2-bromophenoxy)­acetaldehyde (21) (52.0 g, 243.0 mmol) in anhydrous MeOH (250 mL) was stirred at rt for 16 h. Then NaBH_4_ (13.8 g, 364.6 mmol) was added to the mixture at 0 °C. The mixture was warmed to rt and stirred for 1 h before the addition of H_2_O (300 mL). The mixture was concentrated in vacuo to remove the MeOH. The aqueous phase was extracted with EtOAc (300 mL × 3). The combined organic layers were washed with brine (500 mL × 2), dried over Na_2_SO_4,_ filtered and concentrated in vacuo. The residue was purified by silica gel column chromatography (eluent: 25–50% EtOAc in petroleum ether) to afford 42.2 g (58% yield) of 1-(2-(2-bromophenoxy)­ethyl)-5-methylpyrazolidin-3-one (22) as a colorless oil. ^1^H NMR (400 MHz, CDCl_3_): δ 7.98 (br s, 1H), 7.58–7.51 (m, 1H), 7.30–7.23 (m, 1H), 6.91–6.83 (m, 2H), 4.24–4.17 (m, 2H), 3.35–3.26 (m, 1H), 3.22–3.15 (m, 1H), 3.14–3.05 (m, 1H), 2.70 (dd, *J* = 16.4 Hz, 7.6 Hz, 1H), 2.17 (dd, *J* = 16.4 Hz, 9.2 Hz, 1H), 1.32–1.27 (m, 3H). LC/MS (ESI): *m*/*z* = 298.97 [M + H]^+^.

#### 5.2.4 3-Methyl-2,3,5,6-tetrahydro-1*H*-benzo­[*b*]­pyrazolo­[1,2-*d*]­[1,4,5]­oxadiazepin-1-one (23)

A mixture of 1-(2-(2-bromophenoxy)­ethyl)-5-methylpyrazolidin-3-one (22) (34.1 g, 114.0 mmol), CuI (10.9 g, 57.0 mmol), 1,10-phenanthroline (5.14 g, 28.5 mmol) and Cs_2_CO_3_ (74.3 g, 228 mmol) in anhydrous 1,4-dioxane (350 mL) was stirred at 120 °C for 16 h. The mixture was filtered through a pad of Celite, the filter cake was washed with CH_2_Cl_2_ (50 mL × 3). The filtrate was concentrated in vacuo to remove all solvent. The residue was partitioned between H_2_O (500 mL) and CH_2_Cl_2_ (500 mL). The aqueous layer was extracted with CH_2_Cl_2_ (200 mL × 2). The combined organic layers were washed with brine (5 mL × 2), dried over Na_2_SO_4_, filtered and concentrated in vacuo. The residue was purified by silica gel column chromatography (eluent: 20–55% EtOAc in petroleum ether) to afford 12.2 g (49% yield) of 3-methyl-2,3,5,6-tetrahydro-1*H*-benzo­[*b*]­pyrazolo­[1,2-*d*]­[1,4,5] oxadiazepin-1-one (23) as a brown solid. ^1^H NMR (400 MHz, CDCl_3_): δ 7.47 (dd, *J* = 8.0 Hz, 1.6 Hz, 1H), 7.24–7.18 (m, 1H), 7.13–7.06 (m, 2H), 4.37 (d, *J* = 12.0 Hz, 1H), 4.06–3.95 (m, 1H), 3.52–3.50 (m, 1H), 3.45–3.36 (m, 1H), 3.19–3.13 (m, 2H), 2.33–2.22 (m, 1H), 1.35 (d, *J* = 6.84 Hz, 3H). LC/MS (ESI): *m*/*z* = 219.1 [M + H]^+^.

#### 5.2.5 3-Methyl-5,6-dihydro-1*H*-benzo­[*b*]­pyrazolo­[1,2-*d*]­[1,4,5]­oxadiazepin-1-one (24)

**Step 1:** To a mixture of 3-methyl-2,3,5,6-tetrahydro-1*H*-benzo­[*b*]­pyrazolo­[1,2-*d*]­[1,4,5]­oxadiazepin-1-one (23) (12.2 g, 55.9 mmol) in anhydrous THF (250 mL) was added 1 M LHMDS in THF (123 mL, 0.123 mmol) dropwise at −78 °C under N_2_. The mixture was stirred at −78 °C for 5 min before the dropwise addition of a mixture of NBS (9.95 g, 55.9 mmol) in anhydrous THF (150 mL) at −78 °C. The mixture was stirred at −78 °C for 5 min. Saturated NH_4_Cl (200 mL) was then added and the resulting solution was warmed to rt and extracted with EtOAc (1 L × 2). The combined organic layers were washed with brine (500 mL × 2), dried over Na_2_SO_4_, filtered and concentrated down in vacuo to afford a brown oil. This material was used directly in the following step without further purification.
**Step 2:** The crude material was suspended in 1,4-dioxane (160 mL) followed by addition of DBU (8.84 mL, 56.9 mmol). The resulting mixture was stirred at 80 °C for 1 h before cooling to rt and quenching with H_2_O (50 mL). The mixture was concentrated in vacuo to remove the 1,4-dioxane. The mixture was diluted with H_2_O (200 mL) and extracted with CH_2_Cl_2_ (300 mL × 3). The combined organic layers were dried over Na_2_SO_4_, filtered and concentrated in vacuo. The residue was purified by silica gel column chromatography (eluent: 0–50% EtOAc in petroleum ether) to afford 6.52 g (54% yield, over two steps) of 3-methyl-5,6-dihydro-1*H*-benzo­[*b*]­pyrazolo­[1,2-*d*]­[1,4,5]­oxadiazepin-1-one (24) as a yellow solid. ^1^H NMR (400 MHz, CDCl_3_): δ 7.83 (dd, *J* = 8.0 Hz, 2.0 Hz, 1H), 7.27–7.23 (m, 1H), 7.23–7.21 (m, 1H), 7.13 (dd, *J* = 8.0 Hz, 2.0 Hz, 1H), 5.46 (s, 1H), 4.32–4.27 (m, 2H), 3.93–3.87 (m, 2H), 2.23 (s, 3H). LC/MS (ESI): *m*/*z* = 217.1 [M + H]^+^.

#### 5.2.6 2-Amino-3-methyl-5,6-dihydro-1*H*-benzo­[*b*]­pyrazolo­[1,2-*d*]­[1,4,5] Oxadiazepin-1-one (25)

**Step 1:** To a mixture of 3-methyl-5,6-dihydro-1*H*-benzo­[*b*]­pyrazolo­[1,2-*d*]­[1,4,5]­oxadiazepin-1-one (24) (6.60 g, 30.5 mmol) in TFA (30 mL) was added HNO_3_ (68%, 2 mL, 30.5 mmol) dropwise at 0 °C. The mixture was stirred at rt for 1 h before it was poured into ice-cold H_2_O (100 mL). The mixture was filtered, and the residue was dried in vacuo to afford a yellow solid. This material was used in the following step without further purification.
**Step 2:** The crude reaction material was suspended in EtOH/H_2_O (2:1) (90 mL) and DCE (3 mL). Following which Fe^0^ powder (8.52 g, 153 mmol) and NH_4_Cl (1.60 g, 30.5 mmol) were added, the resulting mixture was heated to 80 °C and stirred for 1 h. The reaction mixture was cooled to rt and filtered through a pad of Celite. Filter cake was washed with CH_2_Cl_2_ (50 mL). Filtrate was concentrated in vacuo to remove organic solvents, the resulting solution was extracted with CH_2_Cl_2_ (100 mL × 2). The combined organic layers were dried over Na_2_SO_4_, filtered and concentrated in vacuo to afford 5.10 g (72% yield two steps) of 2-amino-3-methyl-5,6-dihydro-1*H*-benzo­[*b*]­pyrazolo­[1,2-*d*]­[1,4,5]­oxadiazepin-1-one (25) as a brown solid. ^1^H NMR (400 MHz, DMSO-*d*_6_): δ 9.58 (br s, 2H), 7.65 (dd, *J* = 8.0 Hz, 1.6 Hz, 1H), 7.39–7.35 (m, 1H), 7.29–7.22 (m, 2H), 4.32 (t, *J* = 5.2 Hz, 2H), 4.13 (t, *J* = 5.2 Hz, 2H), 2.36 (s, 3H). LC/MS (ESI): *m*/*z* = 232.2 [M + H]^+^.

#### 5.2.7 5-Chloro-2-ethoxy-4-fluorobenzoic Acid (27)

**Step 1:** To a mixture of 4-fluoro-2-hydroxybenzoic acid (26) (50.0 g, 320 mmol) in CH_2_Cl_2_ (500 mL) was added a solution of sulfuryl chloride (129 mL, 1601 mmol) in CH_2_Cl_2_ (300 mL) dropwise at rt, the mixture was stirred at rt for 24 h before the reaction was quenched by addition of H_2_O (400 mL). The mixture was extracted with CH_2_Cl_2_ (400 mL × 3). The combined organic layers were dried over Na_2_SO_4_, filtered and concentrated in vacuo to afford 50.1 g (82% yield) of 5-chloro-4-fluoro-2-hydroxy-benzoic acid as an off white solid. LC/MS (ESI): *m*/*z* = 189.0 [M + H]^+^.
**Step 2:** To a mixture of 5-chloro-4-fluoro-2-hydroxybenzoic acid (50.1 g, 263 mmol) and K_2_CO_3_ (109 g, 789 mmol) in DMF (500 mL) was added iodoethane (63.4 mL, 789 mmol) at rt. The mixture was heated to 70 °C and stirred for 12 h. The mixture was then cooled to rt and filtered. The filtrate was diluted with EtOAc (800 mL) and washed with H_2_O (400 mL × 3). The organic layer was dried over Na_2_SO_4_, filtered and concentrated in vacuo. The residue was purified by silica gel column chromatography (eluent: 5–20% EtOAc in petroleum ether) to afford 36.9 g (57% yield) of ethyl 5-chloro-2-ethoxy-4-fluorobenzoate as a white solid.
**Step 3:** A mixture of ethyl 5-chloro-2-ethoxy-4-fluorobenzoate (36.9 g, 150 mmol) and LiOH·H_2_O (18.0 g, 750 mmol) in THF (600 mL) and H_2_O (200 mL) was stirred at rt for 12 h. The mixture was concentrated in vacuo to remove THF, the aqueous layer was then adjusted to pH = 3 with 2 M HCl and extracted with EtOAc (300 mL × 3). The combined organic layers were dried over Na_2_SO_4,_ filtered and concentrated in vacuo to afford 29.2 g (89% yield) of 5-chloro-2-ethoxy-4-fluorobenzoic acid (27) as a white solid. ^1^H NMR (400 MHz, DMSO-*d*_6_): δ12.90 (s, 1H), 7.79 (d, *J* = 8.8 Hz, 1H), 7.28 (d, *J* = 11.6 Hz, 1H) 4.13 (q, *J* = 7.2 Hz, 2H), 1.33 (t, *J* = 7.2 Hz, 3H). LC/MS (ESI): *m*/*z* = 219.2 [M + H]^+^.

#### 5.2.8 5-Chloro-2-methylbenzofuran-7-carboxylic Acid (29)

**Step 1:** To a mixture of methyl 5-chloro-2-hydroxybenzoate (28) (20.0 g, 107 mmol) in MeCN (200 mL) was added 3-bromoprop-1-yne (10.6 mL, 139 mmol) and K_2_CO_3_ (29.6 g, 214 mmol). The mixture was heated to 80 °C and stirred for 16 h. The mixture was then cooled to rt and concentrated in vacuo. The residue was dissolved in H_2_O (100 mL) and extracted with EtOAc (100 mL × 3). The combined organic layers were dried over Na_2_SO_4_, filtered and concentrated in vacuo. The residue was purified by silica gel column chromatography (eluent: 5–10% EtOAc in petroleum ether) to afford 15.6 g (65% yield) of methyl 5-chloro-2-prop-2-ynoxy-benzoate as a bright yellow solid. ^1^H NMR (400 MHz, CDCl_3_): δ 7.80 (s, 1H), 7.43 (d, *J* = 8.8 Hz, 1H), 7.09 (d, *J* = 8.8 Hz, 1H), 4.79 (s, 2H), 3.90 (s, 3H), 2.55 (s, 1H).
**Step 2:** To a mixture of methyl 5-chloro-2 -prop-2-ynoxybenzoate (5.00 g, 22.3 mmol) in *N*,*N*-diethylaniline (50 mL) was added CsF (4.10 g, 26.7 mmol). The mixture was heated to 200 °C and stirred for 36 h. The reaction was cooled to rt and diluted with H_2_O (100 mL) and the mixture was extracted with EtOAc (50 mL × 3). The combined organic layers were washed with 2 M HCl (100 mL × 5). The organic layer was dried over Na_2_SO_4_, filtered and concentrated in vacuo. The residue was purified by silica gel column chromatography (eluent: 20% EtOAc in petroleum ether) to afford 2.25 g (45% yield) of methyl-5-chloro-2-methyl-benzofuran-7-carboxylate as a brown solid. ^1^H NMR (400 MHz, CDCl_3_): δ 7.80 (d, *J* = 6.0 Hz, 1H), 7.60 (s, 1H), 6.39 (s, 1H), 4.00 (s, 3H), 2.53 (s, 3H). LC/MS (ESI): *m*/*z* = 225.1 [M + H]^+^.
**Step 3:** To a mixture of methyl 5-chloro-2-methyl-benzofuran-7-carboxylate (2.25 g, 9.13 mmol) in EtOH (30 mL) and H_2_O (10 mL) was added LiOH·H_2_O (2.30 g, 54.8 mmol). The mixture was stirred at rt for 16 h. The mixture was adjusted to pH = 3 with 2 M HCl and extracted with EtOAc (10 mL × 3). The combined organic layers were dried over Na_2_SO_4_, filtered and concentrated in vacuo to afford 1.23 g (64% yield) of 5-chloro-2-methyl-benzofuran-7-carboxylic acid (29) as a white solid. ^1^H NMR (400 MHz, DMSO-*d*_6_): δ 13.44 (s, 1H) 7.86 (s, 1H), 7.66 (s, 1H), 6.67 (s, 1H), 2.50 (s, 3H). LC/MS (ESI): *m*/*z* = 211.0 [M + H]^+^.

#### 5.2.9 Ethyl 4-amino-3,3-dimethylbutanoate (31)

**Step 1:** To a mixture of ethyl 3-methylbut-2-enoate (30) (100 g, 781 mmol) in MeCN (500 mL) was added nitromethane (209 mL, 3904 mmol) and DBU (1745 mL, 1171 mmol). The resulting solution was heated to 80 °C and stirred for 56 h. The reaction mixture was then cooled to rt and poured into H_2_O (300 mL). The mixture was then extracted with EtOAc (200 mL × 3). The combined organic layers were dried over Na_2_SO_4,_ filtered and concentrated in vacuo. The residue was purified by silica gel column chromatography (eluent: 10–20% EtOAc in petroleum ether) to afford 134 g (91% yield) of ethyl 3,3-dimethyl-4-nitro-butanoate as a colorless oil. ^1^HNMR (400 MHz, CDCl_3_): δ 4.49 (s, 2H), 4.10 (q, *J* = 7.2 Hz, 2H), 2.40 (s, 2H), 1.22 (t, *J* = 7.2 Hz, 3H), 1.12 (s, 6H). LC/MS (ESI): *m*/*z* = 190.2 [M + H]^+^.
**Step 2:** To a mixture of ethyl 3,3-dimethyl-4-nitro-butanoate (30.0 g, 159 mmol) in EtOH (600 mL) was added 10% Pd/C (10 g). The mixture was heated to 50 °C and stirred for 48 h under H_2_ (50 psi). The mixture was then filtered, and the filter cake washed with EtOH (200 mL). The filtrate was concentrated to afford 19.9 g (79% yield) of ethyl 4-amino-3,3-dimethylbutanoate (31) as a colorless oil. ^1^H NMR (400 MHz, CDCl_3_): δ 8.15–8.05 (m, 2H), 4.12–4.01 (q, *J* = 7.0 Hz, 2H), 3.67 (q, *J* = 7.0 Hz, 2H), 2.40 (s, 2H), 1.18 (t, *J* = 7.0 Hz, 3H), 1.10 (s, 6H).

#### 5.2.10 4-(5-Chloro-2-ethoxy-4-fluorobenzamido)-3,3-dimethylbutanoic Acid (32)

**Step 1:** To a mixture of 5-chloro-2-ethoxy-4-fluorobenzoic acid (27) (10.0 g, 45.7 mmol) in THF (100 mL) was added HATU (26.1 g, 68.6 mmol). The solution was stirred at rt for 30 min. Followed by the addition of ethyl 4-amino-3,3-dimethyl-butanoate (31) (8.01 g, 50.3 mmol) and TEA (19.1 mL, 137 mmol). The reaction mixture was stirred at rt for 16 h. The mixture was poured into H_2_O (100 mL) and extracted with EtOAc (80 mL × 3). The combined organic layers were dried over Na_2_SO_4,_ filtered and concentrated in vacuo. The residue was purified by silica gel column chromatography (eluent: 5–10% EtOAc in petroleum ether) to afford 7.88 g (48% yield) of ethyl 4-[(5-chloro-2-ethoxy-4-fluoro-benzoyl)­amino]-3,3-dimethyl-butanoate as a colorless oil. ^1^H NMR (400 MHz, CDCl_3_): δ 8.22 (d, *J* = 8.8 Hz, 1H), 8.04–7.95 (t, *J* = 5.6 Hz, 1H), 6.74 (d, *J* = 10.6 Hz, 1H), 4.12 (m, *J* = 7.2, 18.7 Hz, 4H), 3.38 (d, *J* = 6.4 Hz, 2H), 2.24 (s, 2H), 1.50 (t, *J* = 6.8 Hz, 3H), 1.26–1.18 (t, *J* = 7.2 Hz, 3H), 1.03 (s, 6H). LC/MS (ESI): *m*/*z* = 360.1 [M + H]^+^.
**Step 2:** To a mixture of ethyl 4-[(5-chloro-2-ethoxy-4-fluorobenzoyl)­amino]-3,3-dimethyl-butanoate (7.88 g, 21.9 mmol) in THF (150 mL) and H_2_O (50 mL) was added LiOH·H_2_O (9.21 g, 219 mmol). The mixture was stirred at rt for 48 h. Upon completion, the mixture was poured into H_2_O (100 mL) and adjusted to pH = 2–3 by conc. HCl. The mixture was filtered, the residue was washed with H_2_O (200 mL) and dried in vacuo to afford 4.87 g (67% yield) of 4-(5-chloro-2-ethoxy-4-fluorobenzamido)-3,3-dimethylbutanoic acid (32) as a pink solid. ^1^H NMR (400 MHz, DMSO-*d*_6_): δ 12.85–11.68 (s, 1H), 8.21–8.07 (t, *J* = 8.9 Hz, 1H), 7.83 (d, *J* = 8.9 Hz, 1H), 7.29 (d, *J* = 11.5 Hz, 1H), 4.17 (d, *J* = 6.9 Hz, 2H), 3.26 (d, *J* = 6.3 Hz, 2H), 2.17 (s, 2H), 1.39 (t, *J* = 7.0 Hz, 3H), 0.98 (s, 6H). LC/MS (ESI): *m*/*z* = 332.1 [M + H]^+^.

#### 5.2.11 4-(5-Chloro-2-methylbenzofuran-7-carboxamido)-3,3-dimethylbutanoic Acid (33)

**Step 1:** To a mixture of 5-chloro-2-methyl-benzofuran-7-carboxylic acid (29) (0.90 g, 4.28 mmol) in THF (10 mL) was added HATU (2.44 g, 6.41 mmol). The mixture was stirred at rt for 30 min. Followed by addition of ethyl 4-amino-3,3-dimethyl-butanoate (31) (0.68 g, 4.28 mmol) and TEA (0.94 mL, 12.8 mmol). The mixture was stirred at rt for 16 h. The mixture was concentrated in vacuo and the residue was diluted with H_2_O (10 mL) and extracted with EtOAc (10 mL × 3). The combined organic layers were dried over Na_2_SO_4_, filtered and concentrated in vacuo. The residue was purified by silica gel column chromatography (eluent: 20% EtOAc in petroleum ether) to afford 0.602 g (40% yield) of ethyl 4-[(5-chloro-2-methyl-benzofuran-7-carbonyl) amino]-3,3-dimethylbutanoate as a colorless oil. ^1^H NMR (400 MHz, CDCl_3_): δ 7.98 (s, 1H), 7.84 (t, *J* = 6.4 Hz, 1H), 7.56 (s, 1H), 6.44 (s, 1H), 4.17 (q, *J* = 7.2 Hz, 2H), 3.51 (d, *J* = 6.4 Hz, 2H), 2.56 (s, 3H), 2.31 (s, 2H), 1.28 (t, *J* = 7.2 Hz, 3H), 1.18 (s, 6H). LC/MS (ESI): *m*/*z* = 352.1 [M + H]^+^.
**Step 2:** To a mixture of ethyl 4-[(5-chloro-2-methyl-benzofuran-7-carbonyl)­amino]-3,3-dimethyl-butanoate (600 mg, 1.71 mmol) in THF (6 mL) and H_2_O (2 mL) was added LiOH·H_2_O (359 mg, 8.55 mmol). The mixture was stirred at rt for 8 h. The mixture was adjusted to pH = 3 with 2 M HCl and extracted with EtOAc (10 mL × 3). The combined organic layers were dried over Na_2_SO_4_, filtered and concentrated in vacuo to afford 0.271 g (49% yield) of 4-(5-chloro-2-methylbenzofuran-7-carboxamido)-3,3-dimethylbutanoic acid (33) as a brown gum. LC/MS (ESI): *m*/*z* = 324.2 [M + H]^+^.

#### 5.2.12 5-Chloro-*N*-(2,2-dimethyl-4-((3-methyl-1-oxo-5,6-dihydro-1*H*-benzo­[*b*]­pyrazolo­[1,2-*d*]­[1,4,5]­oxadiazepin-2-yl)­amino)-4-oxobutyl)-2-ethoxy-4-fluorobenzamide (1)

To a mixture of 4-(5-chloro-2-ethoxy-4-fluorobenzamido)-3,3-dimethylbutanoic acid (32) (43 mg, 0.13 mmol) and DIPEA (0.066 mL, 0.38 mmol) in DMSO (2 mL) was added HATU (58 mg, 0.15 mmol) at rt. The mixture was stirred at rt for 15 min after which 2-amino-3-methyl-5,6-dihydro-1*H*-benzo­[*b*]­pyrazolo­[1,2-*d*]­[1,4,5]­oxadiazepin-1-one (25) (30 mg, 0.13 mmol) was added. The mixture was stirred at rt for 16 h. The crude reaction mixture was purified by preparatory HPLC (eluent: 0–100% MeCN in H_2_O) to afford 13.4 mg (19% yield) of 5-chloro-*N*-(2,2-dimethyl-4-((3-methyl-1-oxo-5,6-dihydro-1*H*-benzo­[*b*]­pyrazolo­[1,2-*d*]­[1,4,5]­oxadiazepin-2-yl)­amino)-4-oxobutyl)-2-ethoxy-4-fluorobenzamide (1) as a white solid. ^1^H NMR (400 MHz, CDCl_3_): δ 8.94 (s, 1H), 8.21 (d, *J* = 8.8 Hz, 1H), 8.15 (t, *J* = 6.6 Hz, 1H), 7.77–7.70 (m, 1H), 7.21–7.13 (m, 1H), 7.13–7.02 (m, 2H), 6.70 (d, *J* = 10.5 Hz, 1H), 4.24 (t, *J* = 4.9 Hz, 2H), 4.09 (q, *J* = 7.0 Hz, 2H), 3.85 (t, *J* = 4.9 Hz, 2H), 3.46 (d, *J* = 6.5 Hz, 2H), 2.22 (s, 2H), 2.17 (s, 3H), 1.45 (t, *J* = 6.9 Hz, 3H), 1.02 (s, 6H). LC/MS (ESI): *m*/*z* = 545.1 [M + H]^+^.

#### 5.2.13 5-Chloro-*N*-(2,2-dimethyl-4-((3-methyl-1-oxo-5,6-dihydro-1*H*-benzo­[*b*]­pyrazolo­[1,2-*d*]­[1,4,5]­oxadiazepin-2-yl)­amino)-4-oxobutyl)-2-methylbenzofuran-7-carboxamide (2)

To a mixture of 4-(5-chloro-2-methylbenzofuran-7-carboxamido)-3,3-dimethylbutanoic acid (33) (42 mg, 0.13 mmol) and DIPEA (0.066 mL, 0.38 mmol) in DMSO (2 mL) was added HATU (58 mg, 0.15 mmol) at rt. The mixture was stirred at rt for 15 m after which 2-amino-3-methyl-5,6-dihydro-1*H*-benzo­[*b*]­pyrazolo­[1,2-*d*]­[1,4,5]­oxadiazepin-1-one (25) (30 mg, 0.13 mmol) was added. The mixture was stirred at rt for 16 h. The crude reaction mixture was purified by preparatory HPLC (eluent: 0–100% MeCN in H_2_O) to afford 11.1 mg (16% yield) of 5-chloro-*N*-(2,2-dimethyl-4-((3-methyl-1-oxo-5,6-dihydro-1*H*-benzo­[*b*]­pyrazolo­[1,2-*d*]­[1,4,5]­oxadiazepin-2-yl)­amino)-4-oxobutyl)-2-methylbenzofuran-7-carboxamide (2) as a white solid. ^1^H NMR (400 MHz, DMSO-*d*_6_): δ 9.17 (s, 1H), 8.44 (t, *J* = 6.3 Hz, 1H), 7.77 (d, *J* = 2.1 Hz, 1H), 7.63 (d, *J* = 8.0 Hz, 1H), 7.57 (d, *J* = 2.2 Hz, 1H), 7.30 (t, *J* = 7.7 Hz, 1H), 7.21 (t, *J* = 7.6 Hz, 2H), 6.69 (s, 1H), 4.28 (t, *J* = 5.0 Hz, 2H), 3.98 (t, *J* = 5.1 Hz, 2H), 3.38 (d, *J* = 6.3 Hz, 2H), 2.48 (s, 3H), 2.31 (s, 2H), 2.11 (s, 3H), 1.08 (s, 6H). LC/MS (ESI): *m*/*z* = 537.2 [M + H]^+^.

#### 5.2.14 6-Amino-*N*-(1,5-dimethyl-3-oxo-2-phenyl-2,3-dihydro-1*H*-pyrazol-4-yl)­bicyclo­[4.1.1]­octane-1-carboxamide Hydrochloride (35)

**Step 1:** To a mixture of 6-((*tert*-butoxycarbonyl)­amino)­bicyclo­[4.1.1]­octane-1-carboxylic acid (34) (100 mg, 0.371 mmol) in CH_2_Cl_2_ (2.0 mL) was added DIPEA (0.194 mL, 1.11 mmol) and HATU (156 mg, 0.411 mmol) at rt. The reaction mixture was stirred for 15 min at rt after which 2,4-amino-1,5-dimethyl-2-phenyl-1,2-dihydro-3H-pyrazol-3-one (76.0 mg, 0.374 mmol) was added. The reaction was stirred at rt for 16 h. The mixture was concentrated in vacuo and the residue was purified by silica gel column chromatography (eluent: 0–10% MeOH in CH_2_Cl_2_) to afford 169 mg (99% yield) of *tert*-butyl (6-((1,5-dimethyl-3-oxo-2-phenyl-2,3-dihydro-1*H*-pyrazol-4-yl)­carbamoyl)­bicyclo­[4.1.1]­octan-1-yl)­carbamate as a yellow solid. ^1^H NMR (400 MHz, CDCl_3_): δ 7.71 (s, 1H), 7.48 (t, *J* = 7.7 Hz, 2H), 7.40 (d, *J* = 7.8 Hz, 2H), 7.33 (t, *J* = 7.3 Hz, 1H), 4.81 (s, 1H), 3.11 (s, 3H), 2.41–2.31 (m, 2H), 2.27 (s, 3H), 1.80 (td, *J* = 14.4, 6.0 Hz, 10H), 1.44 (s, 9H). LC/MS (ESI): *m*/*z* = 455.75 [M + H]^+^.
**Step 2:** To a mixture of *tert*-butyl (6-((1,5-dimethyl-3-oxo-2-phenyl-2,3-dihydro-1*H*-pyrazol-4-yl)­carbamoyl)­bicyclo­[4.1.1]­octan-1-yl)­carbamate (169 mg, 0.371 mmol) in CH_2_Cl_2_ (2.0 mL) was added 4 M HCl in 1,4-dioxane (0.371 mL, 1.48 mmol) over 5 min at rt. The mixture was stirred at rt for 16 h. The reaction mixture was concentrated in vacuo to afford 145 mg (99% yield) of 6-amino-*N*-(1,5-dimethyl-3-oxo-2-phenyl-2,3-dihydro-1*H*-pyrazol-4-yl)­bicyclo­[4.1.1]­octane-1-carboxamide hydrochloride (35) as an orange solid. LC/MS (ESI): *m*/*z* = 355.25 [M + H]^+^.

#### 5.2.15 6-Chloro-*N*-(6-((1,5-dimethyl-3-oxo-2-phenyl-2,3-dihydro-1*H*-pyrazol-4-yl)­carbamoyl)­bicyclo­[4.1.1]­octan-1-yl)-2-methylimidazo­[1,2-*b*]­pyridazine-8-carboxamide (18)

To a mixture of 6-chloro-2-methylimidazo­[1,2-*b*]­pyridazine-8-carboxylic acid (28.4 mg, 0.134 mmol) in DMSO (0.45 mL) was added DIPEA (0.0930 mL, 0.536 mmol) and HATU (61.2 mg, 0.161 mmol). The reaction mixture was stirred at rt for 15 min after which 6-amino-*N*-(1,5-dimethyl-3-oxo-2-phenyl-2,3-dihydro-1*H*-pyrazol-4-yl)­bicyclo­[4.1.1]­octane-1-carboxamide hydrochloride (35) (55.0 mg, 0.141 mmol) was added. The reaction was stirred at rt for 16 h. The mixture was then purified by preparatory HPLC (eluent: 20–100% MeCN in H_2_O) to afford 29.4 mg (40% yield) of 6-chloro-*N*-(6-((1,5-dimethyl-3-oxo-2-phenyl-2,3-dihydro-1*H*-pyrazol-4-yl)­carbamoyl)­bicyclo­[4.1.1]­octan-1-yl)-2-methylimidazo­[1,2-*b*]­pyridazine-8-carboxamide (18) as a white solid. ^1^H NMR (400 MHz, DMSO-*d*_6_): δ 9.90 (s, 1H), 8.69 (s, 1H), 8.27 (s, 1H), 7.61 (s, 1H), 7.49 (t, *J* = 7.8 Hz, 2H), 7.31 (dd, *J* = 15.7, 7.8 Hz, 3H), 3.02 (d, *J* = 2.6 Hz, 3H), 2.53 (s, 3H), 2.46 (s, 4H), 2.07 (s, 3H), 1.93 (s, 2H), 1.80 (s, 6H). LC/MS (ESI): *m*/*z* = 548.3 [M + H]^+^.

### 5.3 Biology

#### 5.3.1 RUVBL Protein Purification

The human full length RUVBL1 and RUVBL2 containing an *N*-terminal 10 × His tag followed by a tobacco etch virus (TEV) protease site in pET28a vector were expressed separately in *E. coli* BL21 (DE3) cells (New England Biolabs, C2527I). Cells were grown in TB media at 37 °C until OD600 reached 0.6 and then induced with 0.25 mM IPTG to grow overnight at 16 °C and harvested the next day. The harvested cells were resuspended in lysis buffer (20 mM Tris–HCl, pH 8.0, 300 mM NaCl, 5 mM MgCl_2_, 30 mM imidazole, supplemented with complete EDTA-free protease inhibitor cocktail (Sigma, 11873580001, 1 tablet/50 mL lysis buffer, 1 μL lysozyme (71110-6000KU) and 1 mM PMSF). The cell suspension was sonicated at 20% amplitude, 1 s on, 1 s off with 35 min total process time. The mixture was then centrifuged for 1 h, 16,000g at 4 °C. The lysate was then filtered with Steriflip (SCGP00525) and loaded onto Ni-NTA column (HiTrap SP HP 5 mL) by using AKTA pure (Cytiva) and eluted with a linear gradient of imidazole (30–400 mM) in lysis buffer. Fractions from Ni-NTA elutions were examined by SDS Page gel, and the chosen fractions were pooled and concentrated into SEC buffer (20 mM HEPEs, 300 mM NaCl, 5 mM MgCl_2_, 10% Glycerol, 1 mM TCEP) to further purify by size-exclusion chromatography using a Superose 6 Increase (16/600). The separated peaks were examined and stored in SEC buffer at −80 °C.

#### 5.3.2 ADP-Glo Assay

The ATPase activity of RUVBL1/2 complexes was measured using ADP-Glo assay kit (Promega, V9102). RUVBL1 and RUVBL2 were each prepared at 8 μM in assay buffer (50 mM Tris HCl, pH 7.5, 20 mM MgCl_2_, 1 mM DTT, 0.1% glycerol, and 0.01% Triton X-100). Equal volumes were mixed to yield a 4x protein complex and incubate on ice for 30 min, then diluted to a 2x protein complex prior to use. For ATP titration, a 2-fold ATP dilution series (0 to 320 μM final) was added (2 μL per well) into a 384-well ProxiPlate. Assay was initiated by adding 2 μL of the 2x protein complex to each well (total volume 4 μL, final protein concentration is 1 μM). For inhibitor testing, 40 nL of DMSO or compound in DMSO was dispensed into wells using an Echo acoustic dispenser. Then 2 μL of 2x protein complex and 2 μL of 320 μM ATP were added (final DMSO = 1%). Plates were incubated at 37 °C for 1 h. After reaction, plates were equilibrated to rt; 4 μL of ADP-Glo reagent was added and incubated at rt for 40 min, followed by 8 μL of kinase detection reagent for another 40 min before measuring luminescence on Envision (PerkinElmer, 2105). To assess assay quality, the *Z*′-factor was calculated using the formula

$$Z^{\prime }=1-\backslash (3\times \left(\sigma_{AC}+\sigma_{NC}\right)/\left|\mu_{AC}-\mu_{NC}\right|$$

where μ_AC_ and μ_NC_ are the means of the active and negative control wells, respectively, and σ_AC_ and σ_NC_ are their corresponding standard deviation.

### 5.4 Tissue Culture and Cell Lines

HCT116 cells (human colorectal carcinoma cell line) and Ramos cells (human Burkitt lymphoma cell line) were obtained from the American Type Culture Collection (ATCC, CCL-247 and CRL-1596) and were cultured in Gibco RPMI-1640 medium (ThermoFisher, 72400120) supplemented with 10% fetal bovine serum (FBS; ThermoFisher, 16000044) and 1% penicillin/streptomycin (ThermoFisher, 15140122) in a humidified tissue culture incubator (37 °C, 5% CO_2_). U2OS cells, also obtained from ATCC (HTB-96), were grown in Gibco DMEM + GlutaMAX (ThermoFisher, 1056601) supplemented with 10% FBS and 1% penicillin/streptomycin and maintained in a humidified 37 °C at 5% CO_2_. Cells were expanded using 10-layer flasks to create a large working stock and frozen at 10 million cells per vial in 1 mL of freezing media containing 90% FBS and 10% DMSO. For the MYC immunofluorescence assay, HCT116 cells were cultured in Gibco McCoy’s 5A Medium (ThermoFisher, 16600082) containing 10% fetal bovine serum (FBS), 1X GlutaMAX supplement (ThermoFisher, 35050061) 1X MEM nonessential amino acids solution (ThermoFisher, 11140076) and 1% penicillin/streptomycin (ThermoFisher, 15140122). All adherent cells were trypsinized with TrypLE (ThermoFisher, 12604013) prior to passaging or cell counting.

### 5.5 In Vitro Cell Viability Assay in HCT116 Cells

To run a cell viability test, HCT116 cells were counted on a Bio-Rad TC20 cell counter, then seeded into 384-well white flat-bottom polystyrene plates (Corning, 3570) at 500 cells/25 μL/well using Multidrop Combi (ThermoFisher). After cells were incubated in the tissue culture incubator for 24 h, a 2-fold dilution series of the test compound (using DMSO as diluent) in 25 μL of growth media were then added to the cells (with highest final concentration of 10 μM) with well duplicate per concentration. After 72 h of compound incubation, cell viability was measured using the CellTiterGlo assay (Promega, cat# G7572) according to manufacturer’s instructions. Luminescence levels were detected on an EnVision Plate Reader (PerkinElmer). Raw data were normalized and fit to 4-parameter sigmoidal dose–response curve. In addition to HCT116 WT cells, CB-6644 drug-resistant HCT116 were assayed utilizing the same cell viability assay protocol. Drug-resistant HCT116, were generated from WT HCT116 cells grown in increasing concentrations of CB-6644, with isolation of single-colonies expanded in the continued presence of 300 nM CB-6644. Subsequently, genomic DNA was isolated from cell pellets of respective drug-resistant clones utilizing the DNEasy kit (Qiagen, Germany) and PCR amplified utilizing PCR primers (Life Technologies, Integrated DNA Technologies; Table S1) targeting exonic regions of RUVBL1 and RUVBL2 at an annealing temperature of 57 °C for 35 cycles. PCR product was assessed for purity by gel electrophoresis and sent to Elim Biopharm (Hayward, CA) for genomic sequencing. Sequence analysis was performed utilizing the Benchling platform (San Francisco, CA) in comparison to the human RUVBL1 (Ensembl ENST00000322623) and RUVBL2 reference sequences (Ensembl ENST00000595090) (Figure S8).

### 5.6 In Vitro Cell Viability Assay in Ramos Cells

Ramos cell viability assessments, counted suspension cells on a Vi-Cell Blu automated cell counter (Beckman Coulter), followed by cell-seeding into 384-well white flat-bottom polystyrene plates (Corning, 3570) at 1000 cells/50 μL/well using Multidrop Combi (ThermoFisher). After cells were incubated in the tissue culture incubator for 24 h, a 3-fold dilution series of the test compound (using DMSO as diluent) with backfilling for DMSO normalization were then added to the wells by Tecan D300e Digital Dispenser (with highest final concentration of 3 μM) with well triplicates per concentration. After 72 h of compound incubation, cell viability was measured using the CellTiterGlo assay (Promega, G7572) according to manufacturer’s instructions. Luminescence levels were detected on an EnVision Plate Reader (PerkinElmer). Raw data were normalization and fit to 4-parameter sigmoidal dose–response curve (Graphpad Prism 10 Software).

### 5.7 MYC Western Blotting

HCT116 cells were harvested using 1x TrypLE Express Enzyme (ThermoFisher, 12604-021), after washing and aspiration with PBS. Viable cells were washed, plated, and incubated overnight at 37 °C. Treated cells were incubated for their respective periods and harvested sequentially for nuclear and cytoplasmic extraction and whole cell extractions. Nuclear and cytoplasmic extractions were performed following manufacturer’s extraction protocol (Pierce, 78835A). Whole-cell lysates were harvested using RIPA Lysis Buffer (Pierce, 89900) with Protease/Phosphatase Inhibitor (Cell Signaling Technology, 5872S). Cells were placed on 4 °C rocker for 5 min. Whole-cell lysates were respectively sonicated for a total time of 12 s (cycles of 2 s on, 2 s off) using a Fisher Scientific Sonic Dismembrator (model 500). Cell lysates were incubated on ice for 15 min and then centrifuged at 16,100g for 14 min. Supernatant was transferred to prechilled tubes for BCA quantification. Respective lysate suspensions were loaded onto a 12–230 kDa ProteinSimple separation module (BioTechne, SM-W001) per manufacturer’s protocol. Primary rabbit antibodies against MYC (Cell Signaling Technology, 9402), TBP (Cell Signaling Technology, 44059), RUVBL1 (Sigma-Aldrich, HPA019947), RUVBL2 (Santa Cruz Biotechnology, sc-374135), V5-tag (Invitrogen, R96025), β-Actin (Cell Signaling Technology, 8457), Halo (Promega, G9211) and Vinculin (Abcam, ab91459) were run on the Jess automated Western blotting system (BioTechne, 004-560) and detected using the antirabbit HRP secondary reagents (BioTechne, 042-206) or antimouse HRP secondary reagents (BioTechne, 042-205). Quantification of protein expression was performed using the Compass for SimpleWestern software (BioTechne).

#### 5.7.1 MYC Immunofluorescence Assay

HCT116 cells were seeded in 384-well black clear-bottom optical plastic plates (Greiner Bio-One, 781097) at 1250 cells per well in 50uL of media. The following day, cells were treated with compound using a Beckman Coulter Echo 655 acoustic liquid handler to perform a 10-point dose response with 3.16-fold dilution series and 10 μM top concentration. Cells were incubated for 24 h then fixed with 4% paraformaldehyde in DPBS for 10 min at RT. Plates were then washed twice with DPBS using a BlueCat Bio BlueWasher prior to blocking and permeabilization in immunofluorescence assay buffer consisting of DPBS with 0.1% Triton X-100 and 10% goat serum for 30 min at RT. Plates were decanted and then incubated overnight at 4 °C with rabbit anti-MYC clone D3N8F (Cell Signaling Technology) diluted 1:1000 in immunofluorescence assay buffer. The following day, plates were decanted and stained for 30 min at RT with goat antirabbit IgG Alexa Fluor Plus 647 (ThermoFisher, A32733) diluted 1:1000 in immunofluorescence assay buffer with Hoechst (AnaSpec, AS83218) to counterstain nuclei. Plates were then decanted and washed twice with DPBS using the BlueWasher. Plates were sealed then imaged using an ImageXpress Micro slit confocal microscope (Molecular Devices) using a 40x water immersion objective to image six fields of view per well. Images were analyzed using MetaXpress Custom Module Editor, in which background-subtracted nuclear MYC intensity was measured by using a Hoechst mask to identify nuclei, then subtracting the background (extra-nuclear) average MYC intensity from the average nuclear MYC intensity. Compound data were normalized to DMSO and positive control compound CB-6644. The effective compound concentration leading to a 50% loss of nuclear MYC intensity (EC_50_) relative to DMSO and CB-6644 was determined by fitting a four-parameter nonlinear regression using GraphPad Prism. At least three technical replicates were analyzed per compound tested.

### 5.8 In Vitro Single-Molecule Tracking Assay

#### 5.8.1 HaloTag Cell Line Engineering

To generate RUVBL1-HaloTag fusion cell lines, a vector comprising the RUVBL1 coding sequence and a Blasticidin resistance gene, linked by a self-cleaving peptide, is electroporated into U2OS cells containing a landing pad construct integrated at the human Rosa26 (hRosa26) locus. The landing pad comprises a HaloTag sequence under the control of a weak L30 promoter and a puromycin resistance gene, also linked by a self-cleaving peptide. Electroporation is performed in the presence of Cas9 protein and a single guide RNA (sgRNA) targeting the *N*-terminal region of the HaloTag sequence within the landing pad. Following electroporation, cells are cultured in the presence of Blasticidin (ThermoFisher, A1113903) at a concentration of 5 μg/mL to select for stably modified cells.

#### 5.8.2 OLS Single-Molecule Tracking Sample Preparation

U2OS RUVBL-halo cells were seeded at 6000 cells per well onto 384-well CellVis glass bottom plates using a Multidrop Combi dispenser (ThermoFisher) and incubated at 37 °C and 5% CO_2_ for 24 h before compound treatment and image acquisition. To label Halo-Tagged proteins RUVBL1^halo^ cells were incubated with 10 pM of JF_549_-HTL (Cat. No. GA1110, Promega) and 100 nM Hoechst 33342 for 1 h in complete medium. Cells were then washed three times in DPBS and twice in imaging media using an EL406 plate washer. Imaging media is fluorobrite DMEM (ThermoFisher, A1896701) supplemented with 1% GlutaMAX (ThermoFisher, 35050079), 1% pen-strep (ThermoFisher, 15140122) and 10% Fetal Bovine Serum (ThermoFisher, 16000044). Compounds were serially diluted in an Echo Qualified 384-Well Low Dead Volume Source Microplate (Beckman Coulter, 0018544) to generate dose-titration source material prior to being transferred at a final 1:1000 dilution using the Echo 655 acoustic dispenser. Compounds are incubated for 4 h at 37 °C prior to image acquisition.

### 5.9 Image Acquisition

Unless otherwise stated, all image acquisition for htSMT was performed on a customized Nikon Eclipse Ti2-E inverted fluorescence microscope with a motorized stage. The microscope system was outfitted with a stage top environmental chamber with temperature and CO_2_ control (OKO laboratories), Nikon objective water dispenser, an Oblique Line Scanning (OLS) illumination module^39^ with laser launch containing 405, 560, and 642 nm lasers, three-band emission filter set (ET 445/58 m, FF01-585/40-25, FF01-676/37-25, Chroma), motorized filter wheel (Lambda 10 B; Sutter Instruments), and a high-speed sCMOS camera equipped with light-sheet mode capability (ORCA-Fusion BT, Hamamatsu). Images were acquired with a 60 × 1.27 NA water immersion objective (CFI SR Plan Apo IR 60XC WI, Nikon, Japan). The environmental chamber was maintained at 37 °C, 95% humidity, and 5% CO_2_. For each field of view, 150 SMT frames were collected at a frame rate of 100 Hz with a 407 microsecond stroboscopic laser pulse, and 1 frame in the Hoechst channel was subsequently collected for downstream registration of trajectories to nuclei. Each frame captured an FOV (field of view) that was 1728 × 2304 pixels (187.14 × 249.52 μm) in size. Automated microscope control and image acquisition was performed using customized scripts in MicroManager.

### 5.10 Image Analysis

SMT data were processed with a custom pipeline with methods, algorithms, and description of software tools utilized, and described in detail by Driouchi et al.,^39^ in addition to information on software and data availability.

### 5.11 Tandem Mass Tags (TMT) Mass-Spectrometry

HCT116 cells were seeded in 15 cm^2^ dishes and respectively treated with 1 μM of compound 1 or DMSO in triplicate. Triplicates for time points 8, 24, and 48 h post-treatment were harvested with TrypLE Express (ThermoFisher, 12604-021) and were respectively assessed for viability using trypan blue in an automated hemacytometer (Vi-Cell Blu; Beckman Coulter). Individual samples were washed twice with precooled PBS using centrifugation (600g) at 4 °C and flash frozen after final aspiration using liquid nitrogen and stored at −80 °C. Samples were processed at Innomics (San Jose, CA) by addition of STrap-compatible lysis buffer, sonicated, and subsequently cleaned up. BCA assays were then performed to determine the concentration of the resulting samples. The resulting samples were processed using the STrap MS sample prep device according to the manufacturer’s protocol (ProtiFi). 100 μg of each sample was processed by reduction with dithiothreitol (DTT) and alkylated with iodoacetamide (IAM). The IAM reaction was then quenched with DTT, and the resulting samples digested in the STrap device with Trypsin/Lys-C overnight. Digested samples were eluted from the STrap according to the manufacturer’s protocol and then dried by vacuum centrifugation (SpeedVac). Dried samples were reconstituted with 50 mM HEPES pH 8.5. All samples were added with their assigned TMT channels. The TMT-added samples were incubated at rt for 1 h for labeling. The same amount of each TMT-labeled sample was aliquoted; and the aliquots were pooled and mixed with 1% formic acid. The resulting mixture was loaded onto the nano LC–MS/MS system for TMT label checking. After confirmation of TMT labeling, all samples were dried by SpeedVac and reconstituted with 2% formic acid. The reconstituted samples were combined. The resulting mixture was further desalted using Evolute Express ABN according to the manufacturer’s protocol (Biotage). The eluted sample was dried by SpeedVac, reconstituted with high-pH reversed-phase buffer A, and subsequently HPLC fractionated. Twelve fractions were obtained from the eluted sample and dried by SpeedVac. All 12 fractions were used for LC–MS/MS analysis, with samples reconstituted with mobile phase A and loaded onto a nano LC–MS/MS system. The same amount of each reconstituted sample was injected for analysis with the TMT method. TMT quantification was performed with Proteome Discoverer 2.5 (ThermoFisher).

### 5.12 Experimental Polar Surface Area (EPSA) Assay

A supercritical fluid chromatography (SFC) method was used to measure the EPSA values of compounds. In this SFC method, the column used was Chirex 3014 (*S*)-Valine and (*R*)-1-(alpha-naphthyl)­ethylamine column from Phenomenex (5 μm, 4.6 × 50 mm, 120 Å). Mobile phase A was CO_2_, and mobile phase B was 20 mM ammonium formate in MeOH. The gradient applied was increasing mobile phase B from 5% to 65% from 0 to 4 min. The column temperature was 40 °C, the flow rate was 4 mL/min, and the automated back pressure regulator (ABPR) was set to 2000 psi. Compounds with concentration of 300 μM in DMSO were injected at 2 μL. Calculated EPSA values are based on a calibration curve using retention times of compounds with known literature values (Table 5) from Goetz, G. H.; Philippe, L.; Shapiro, M. J. EPSA: A Novel Supercritical Fluid Chromatography Technique Enabling the Design of Permeable Cyclic Peptides. *ACS Med Chem Lett* 2014, *5* (10), 1167-1172. 10.1021/ml500239m. The retention times were evaluated at 254 nm.

**Table 5:** EPSA Values of Compounds from the Literature

compound	EPSA value from literature
antipyrine	61
chlorpromazine	68
desipramine	87
pindolol	103
diclofenac	135
*m*-nitrobenzoic acid	157
bumetanide	185
furosemide	230

### 5.13 In Vivo Mouse PK Assay

Study was conducted to characterize the single dose pharmacokinetics of compounds in the male CD1 mice after oral administration (10 mg/kg). A group of fed male CD1 mice (n = 3, Approximately 20–30 g) were administered a single oral dose of compounds as a solution in 5% *N*-methyl pyrrolidone (NMP)/20% PEG400/5% Solutol/70% water, or as a suspension in 0.5% methylcellulose 400cps/0.5% Tween 80 or 10 mM Sodium Citrate buffer pH 4.5. Amorphous solid dispersions (ASD) of compound 2 were prepared by spray-drying in a solution of methanol and polymer on a Buchi B-290 mini-spray dryer for both Hydroxypropylmethyl cellulose acetate succinate (HPMC-AS-HG) and Eudragit L-100 polymers. ASD were confirmed to be amorphous by characterization by X-ray powder diffraction (XRPD), differential scanning calorimetry (DSC), thermogravimetric analysis (TGA), and polarized light microscopy. Drug loading (DL) and solubility was determined using HPLC analysis. Suspensions of 20–30% drug loading compound 2 Amorphous solid dispersions (ASD) were prepared in 10 mM Sodium Citrate buffer pH 4.5 in water for oral dosing in the studies. Compound 2 ASD suspensions were prepared by magnetic stirring to prepare a homogeneous suspension. Blood of each sample was collected at 0.25, 0.5, 1, 2, 4, 8, 24 h post dose and transferred into plastic micro centrifuge tubes containing anticoagulant of K2-EDTA and mixed well with anticoagulant. Plasma samples were obtained from blood samples. Plasma samples were processed in protein precipitation approach prior to LC–MS/MS system for quantitative analysis. Pharmacokinetics parameters were calculated by employing a noncompartmental analysis (PhoenixWinNonlin).

### 5.14 In Vivo Mouse Study

#### 5.14.1 Formulation Preparation

Compound 1 was prepared as a milled suspension in 0.5% methyl cellulose (MC), 0.5% polysorbate 80 (w/v/v) in water for oral dosing in the studies. Compound 1 suspensions were prepared with wet-milling to reduce particle size, using either trituration in a mortar and pestle followed by quantitative transfer to a volumetric flask or bead-milling with gentle rotation and glass beads. This resulted in suspensions with particles approximately 1–50 μm in length, with the majority less than 20 μm by visual assessment on polarized light microscopy. CB-6644 and compound 18 were prepared as a suspension in 0.5% methyl cellulose, 0.5% polysorbate 80 (w/v/v) in water for oral dosing in the studies. CB-6644 and compound 18 suspensions were prepared using 1 min of homogenization with a motorized tissue homogenizer to break apart large aggregates followed by magnetic stirring to prepare a homogeneous suspension. ASD of compound 2 were prepared the way stated above in the PK assays.

### 5.15 Tumor Model and Efficacy Study Dosing

In vivo efficacy of test compounds was evaluated using HCT116 or Ramos tumor xenograft models. HCT116 tumors were established in 4–5 week-old female Crl/NU­(NCr)-Foxn1nu athymic nude mice, and Ramos tumors in 4–5 week-old female NOD.CB17-Prkdcscid/NCrCrl NOD-SCID mice (Charles River Laboratories). Mice were housed five per cage with ad libitum access to food and water. HCT116 and Ramos cell lines (ATCC) were cultured in McCoy’s 5A Medium and RPMI-1640 Medium, respectively, each supplemented with 10% FBS. Xenografts were established via subcutaneous injection of 2 × 10^6^ HCT116 cells or 10 × 10^6^ Ramos cells in a 1:1 mixture of Geltrex and HBSS into the right flank. Body weights and tumor volumes were measured biweekly and recorded in StudyLog. Tumor volume (TV) was calculated using the formula:

TV = 0.5 × length × width^2^. Tumor Growth Inhibition (TGI) was calculated as TGI (%) = [1—((T_final—T_initial)/(C_final—C_initial))] × 100, where T and C represent tumor volumes in treated and control groups, respectively. After tumors grew to an average volume of 150–200 mm^3^ (7–10 days postimplantation), mice were randomized into treatment groups (*n* = 8 mice/group) and received either test compounds or vehicle control daily via oral gavage at 10 mL/kg body weight. Compounds CB-6644, compound 1, and compound 18 were suspended in 0.5% Methylcellulose, 0.5% Tween 80 (w/v/v) in water. Compound 2 was formulated in 10 mM Sodium Citrate buffer pH 4.5. Compounds were administered at 10–150 mg/kg per dose as indicated.

### 5.16 Animal Ethics

All procedures with mice were performed at the Mispro Vivarium facility at 450 East 29th St New York, NY 10016 and were conducted according to the Eikon Therapeutics protocol (number 2022-EIK-01), which was approved by the Mispro Institutional Animal Care and Use Committee (IACUC). Mice were housed in Allentown caging with automatic watering systems. Food (PicoLab Rodent Diet 20, number 5053, labdiets.com) and water (RO treated) were provided ad libitum.

## Supplementary Material







## Notes

Molecular formula strings of test compounds along with assay potencies (CSV)
Molecular docking file of CB-6644 and compound 16 (PDB)
ADP Glo assay development and dose response curves; SMT assay reproducibility correlation plot; MYC level data upon compound treatment; in vivo characterization of compound 1; XRPD and solubility data of different batches of compound 2; activity data for additional compounds; cell line validations; additional assay results of compound 18 and CB-6644; RUVBL1/2 sequencing primers; experimental details for additional ADME assays; representative NMR spectra and HPLC chromatographs for key analogs; synthetic procedures for the preparation of additional compounds (PDF)

## Abbreviations

AC: active control
ARP: actin-related protein
ASD: amorphous solid dispersion
AUC_(0‑last)_: area under the curve (from time zero to last quantifiable time-point)
BID: twice a day
BL: Burkitt Lymphoma
Boc: *tert*-butoxycarbonyl
bp: base-pairs
BSD: blasticidin S deaminase
Caco-2: human colorectal adenocarcinoma (cell-line)
CH_2_Cl_2_: dichloromethane
chromLogD: chromatographic lipophilicity index (at pH 7.4)
c-MYC: cellular-myelocytomatosis
Cryo-EM: cryo-electron microscopy
Cs_2_CO_3_: cesium carbonate
CsF: cesium fluoride
CuI: copper­(I) iodide
DBU: 1,8-diazabicyclo­[5.4.0]­undec-7-ene
DCE: 1,2-dichloroethane
DIPEA: *N*,*N*-diisopropylethylamine
DMF: *N*,*N*-dimethylformamide
DMSO: dimethyl sulfoxide
DSC: differential scanning calorimetry
DTT: dithiothreitol
EtOH: ethanol
Fe^0^: Iron
FOV: field of view
H_2_: dihydrogen (hydrogen gas)
HaloTag: haloalkane dehalogenase protein tag
HAT: histone acetyltransferase
HATU: 1-[bis­(dimethylamino)­methylene]-1*H*-1,2,3-triazolo­[4,5-*b*]­pyridinium 3-oxide hexafluorophosphate
HCl: hydrochloric acid
HCT116: human colorectal carcinoma (cell-line)
HEPES: 4-(2-hydroxyethyl)-1-piperazineethanesulfonic acid
HLM: human liver microsomes
HNO_3_: nitric acid
IAM: iodoacetamide
JF_549_: Janelia Fluor 549
kDa: kilodalton
K_2_CO_3_: potassium carbonate
KOH: potassium hydroxide
LHMDS: lithium bis­(trimethylsilyl)­amide
LipE: lipophilic efficiency
LiOH·H_2_O: lithium hydroxide monohydrate
LogP: logarithm of partition coefficient
mDa: megadalton
MeCN: acetonitrile
MeOH: methanol
Met ID: metabolite identification
MLM: mouse liver microsomes
mut: mutant
MYC: myelocytomatosis
NaBH_4_: sodium borohydride
NBS: *N*-bromosuccinimide
NC: nonactive-control
NH_4_Cl: ammonium chloride
p-CHK2: phospho-checkpoint kinase 2
PCR: polymerase chain reaction
Pd/C: palladium on carbon
PD: pharmacodynamics
PIKK: phosphatidylinositol 3-kinase-related kinase
*P*-gp: *P*-glycoprotein
PPB: plasma protein binding
QD: once daily (“quaque die”)
rt: room temperature
SFC: supercritical fluid chromatography
TBP: TATA-binding protein
TEA: triethylamine
TFA: trifluoroacetic acid
TGA: thermogravimetric analysis
TGI: tumor growth inhibition
THF: tetrahydrofuran
TINTIN: trimer independent of NuA4 involved in transcription interactions with nucleosomes
TMT: tandem mass tags mass-spectrometry
tPSA: topological polar surface area
TRRAP: transformation/transcription domain-associated protein
U2OS: human bone osteosarcoma (cell-line)
V5: V5 epitope tag
WT: wild-type
XRPD: X-ray powder diffraction
