# Supplementary material for: Rapid Optimization Enabled by Single-Molecule Tracking: Discovery of a Potent RUVBL1/2 Inhibitor to Evaluate the Targeting of MYC-Driven Cancers
Source: J Med Chem. 2026 Apr 22;69(10):12048–68. doi: 10.1021/acs.jmedchem.5c03692 (PMC13224169; doi:10.1021/acs.jmedchem.5c03692)
Supplement: Supplementary file 3 [file jm5c03692_si_003.pdf]

Supporting Information

Rapid Optimization Enabled by Single-Molecule Tracking: Discovery of a Potent RUVBL1/2 Inhibitor to Evaluate the Targeting of MYC-Driven Cancers

Li Zheng [a], Eugene Park [a], Jason Lenihan [a], William S. R. Forrest [a], Xin Zhou [a], Charmaine Fong [a], Yangzhong Tang [a], Marcus P. Kelly [a], Amine Driouchi [a], Ali Tabatabaei [a], Helen Wong [a], Jesse D. Vargas [a], Samuel T. Albright [a], Zachary Howard [a], Maité B. Silva [a], Liam A. Elliott [a], Michael Farley [a], José Ortega [a], Stephen Jones [a], Xiao Chang [a], Taylor Heuer [a], Quan Zheng [a], Huntly M. Morrison [a], Daniel Bracho [a], Qian Du [a], Jennifer Le [a], Abhijit Tarafder [a], Grzegorz Nawrocki [a], Patric Schyman [a], Lakshmi Akella [a], Mai K. Nguyen [a], Daisy Ding [a], Arnold Tao [a], Fernando Rodríguez Pérez [a], Kayla VanBuren [a], Rohit Malik [a], Melissa Dumble [a], Daniel J. Anderson [a], Leah Cleary [a], David W. Piotrowski [a], Hilary P. Beck [a]*

[a] Eikon Therapeutics Inc., 230 Harriet Tubman Way, Millbrae, CA 94030, United States

[*] Corresponding Author: Li Zheng – Eikon Therapeutics Inc., 230 Harriet Tubman Way, Millbrae, CA 94030, United States; orcid.org/0000-0002-4228-7797; Email: zhengl@eikontx.com

Table of Contents

Supplemental Figures S2
Materials for RUVBL Sequencing S8
Experimental Procedures for ADME assays S11
Experimental procedures for additional compounds S13
NMR spectra and HPLC traces for key compounds S62

Supplemental Figures

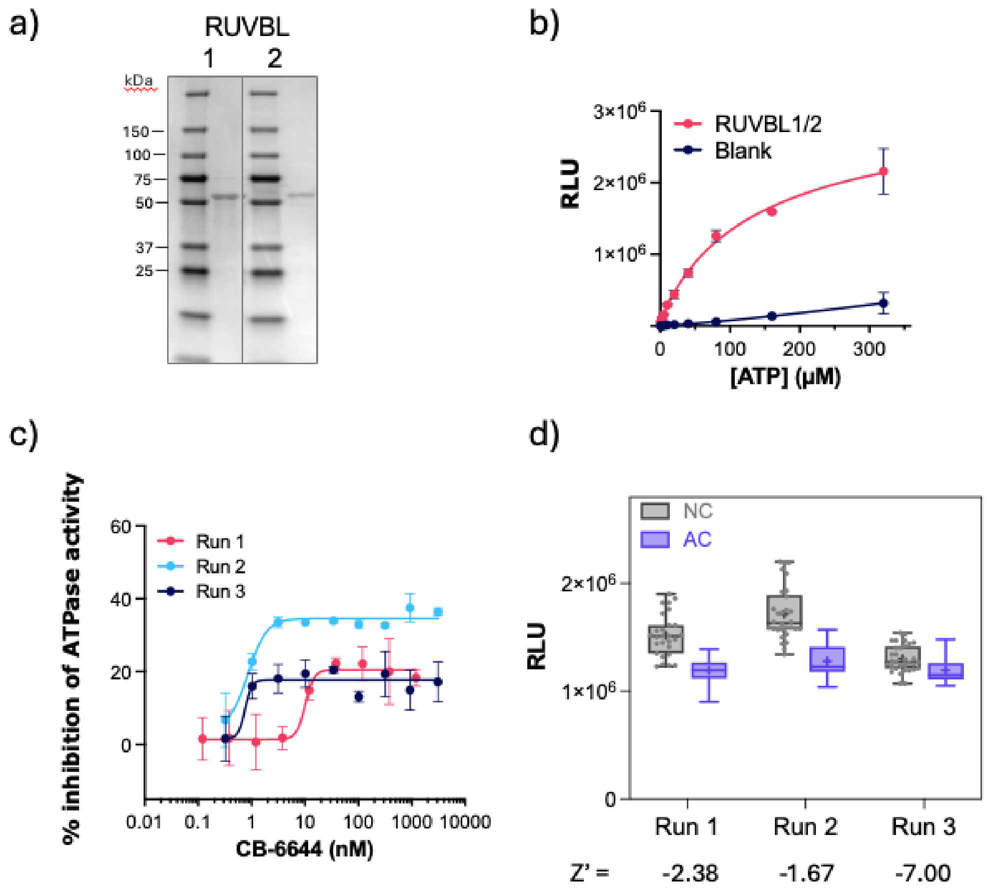

Figure S1 Limited utility of biochemical ADP-Glo™ assay for RUVBL1/2 ATPase screening. a) SDS-PAGE of purified RUVBL1 and RUVBL2. b) ATP-dependent ATPase activity of the reconstituted RUVBL1/2 complex. Luminescence (RLU) is plotted against ATP concentration for assay wells containing RUVBL1/2 (blue) or blank control (black). Data represent mean ± standard deviation (SD) of n = 3 replicates per concentration. Km is fitted as146 µM. c) Dose-response curves of CB-6644 inhibitory activity. Precent inhibition of ATPase activity is plotted versus CB concentration for three independent assays. Data represent mean ± standard deviation (SD) of n = 3 replicates per concentration. No strong inhibition is observed, with maximal inhibition ~30% at the highest tested concentration. d) Distribution of control signals (NC, negative control = DMSO, gray; AC, active control = 1 µM CB-6644, blue) across three independent runs. Box-and-dot plots (boxes: tukey quartiles; whiskers: min-max; mean shown as a "+") show AC and NC raw RLU values. Calculated Z' values are indicated for each run.

a) b)

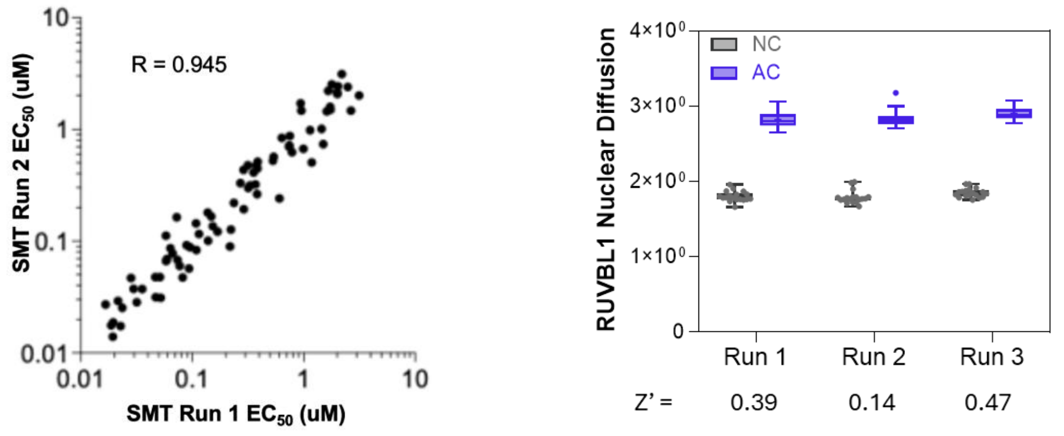

Figure S2 RUVBL SMT assay shows good reproducibility across runs. a) Comparison of two independent htSMT assessments of compound activity in RUVBL1 htSMT indicating a tight correlation of EC_{50} values between both runs (R^2=0.945). b) A graph of RUVBL1 nuclear diffusion and respective Z' values for active and non-active controls from three independent htSMT assessments (n=18 FOVs per AC or NC, per run).

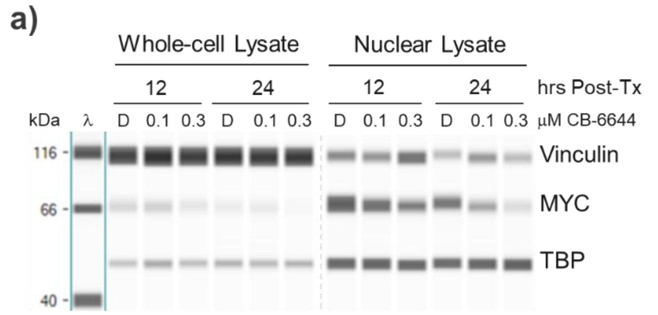

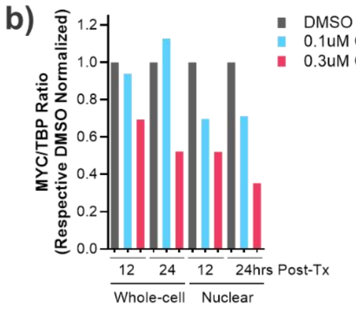

c)

8hr Rank	Peptide Abundance Ratio Compound 1 / DMSO (43 significant POIs)		
POI	8hr	24hr	48hr
MYC	0.492	0.378	0.172
DPCD	0.625	0.489	0.432
POLR2C	0.675	0.499	0.388
POLR2B	0.707	0.557	0.465
NHP2	0.762	0.61	0.573
POLR2L	0.767	0.703	0.57
MLST8	0.779	0.689	0.567
PRPF8	0.78	0.658	0.523
VPS72	0.781	0.844	0.708
POLR2A	0.782	0.709	0.499

24hr Rank	Peptide Abundance Ratio Compound 1 / DMSO (1360 significant POIs)		
POI	8hr	24hr	48hr
MYC	0.492	0.378	0.172
SLC38A2	0.653	0.416	0.277
POLR2I	0.701	0.467	0.378
DPCD	0.625	0.489	0.432
POLR2C	0.675	0.499	0.388
RRM2	0.875	0.509	0.345
CDC20	0.96	0.533	0.369
RPUSD1	1.014	0.549	0.615
POLR2B	0.707	0.557	0.465
POLR2G	0.786	0.596	0.513

48hr Rank	Peptide Abundance Ratio Compound 1 / DMSO (4243 significant POIs)		
POI	8hr	24hr	48hr
ABI2	1.052	0.782	0.131
CSNK1D	0.895	0.615	0.142
PTMA	0.84	0.593	0.145
MYC	0.492	0.378	0.172
DTL	1.295	0.806	0.218
SLC38A2	0.653	0.416	0.277
CCNA2	1.072	0.666	0.29
PJA2	0.863	0.676	0.307
EXO1	1.126	0.756	0.307
SERF2	1.014	0.704	0.311

Only ranked POIs w/pAdj<0.05 listed

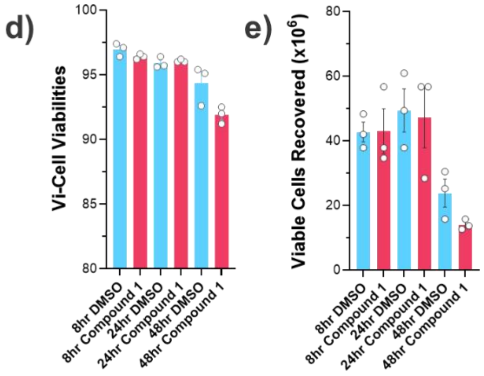

Figure S3 Reduced MYC expression is observed in HCT116 with RUVBL inhibition by CB-6644 and compound 1. a) Automated immunoblot (Jess System, Bio-Techne) of HCT116 whole-cell and nuclear lysates, probed for MYC, cytoplasmic loading control Vinculin, and nuclear loading control TBP. b) Graph of nuclear

MYC to nuclear TBP ratio for whole-cell and nuclear lysates, demonstrating reduced nuclear MYC detection with treatment by both 0.1 and 0.3mM CB-6644 at all treatment durations. c) A table of top ten significantly (pAdj < 0.05) downregulated proteins of interest (POI) for the 8, 24 and 48h treatment durations. d and e) Cell viability and viable cells recovered for respective triplicates of compound 1 treated cells harvested for TMT mass-spectrometry, assessed by trypan blue exclusion (Vi-Cell Blue, Beckman Coulter).

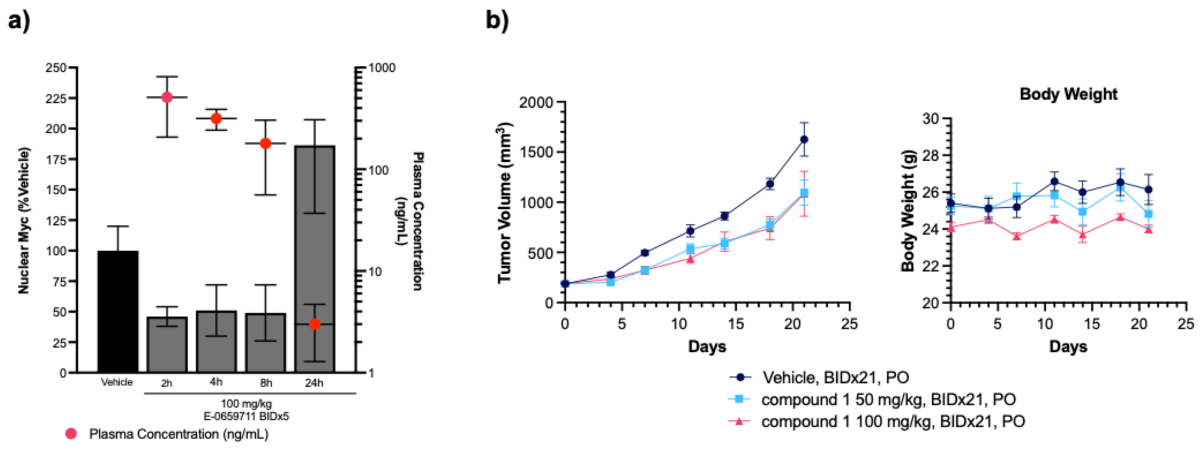

Figure S4. *In vivo* pharmacodynamic assessment of compound 1 in addition to tumor growth and body weight tracking. a) Upon compound 1 treatment, target engagement *in vivo* is observed using nuclear MYC level as PD biomarker in the HCT116 mouse xenograft; b) modest anti-tumor activities are observed with no notable body weight loss in the HCT116 mouse xenograft for compound 1.

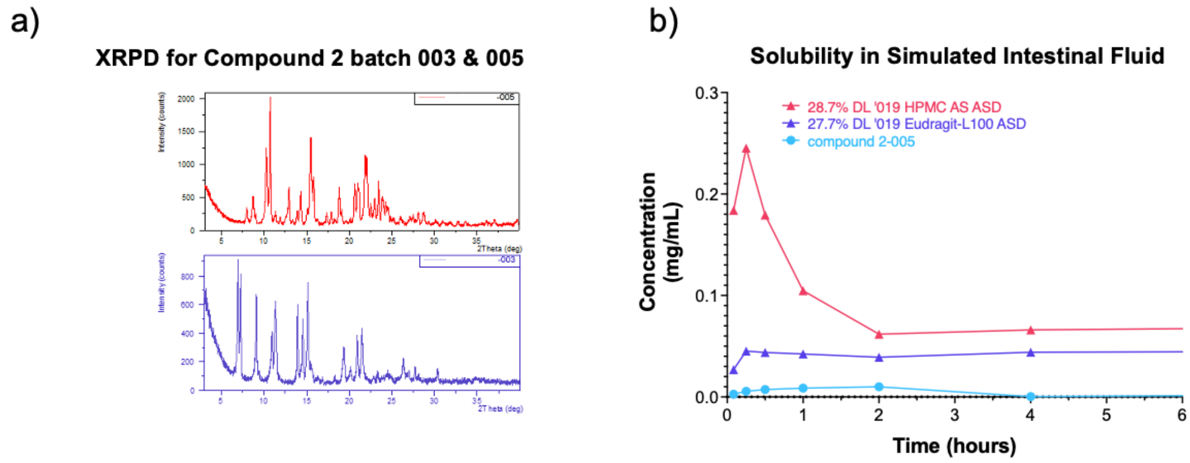

Figure S5. a) XRPD for compound 2 batch 003 and batch 005 indicates that they are different polymorphs; b) both ASD samples increase solubility in the Fasted-state Simulated Intestinal Fluid.

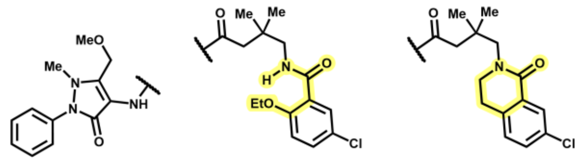

	non-cyclic	cyclic
RUVBL SMT EC_{50} (µM)	0.051	> 5.0
CTG HCT116 EC_{50} (µM)	0.062	> 29.9

Figure S6. SAR around right-hand vector cyclization to mimic IMHB suggested by single crystal XRD.

a)

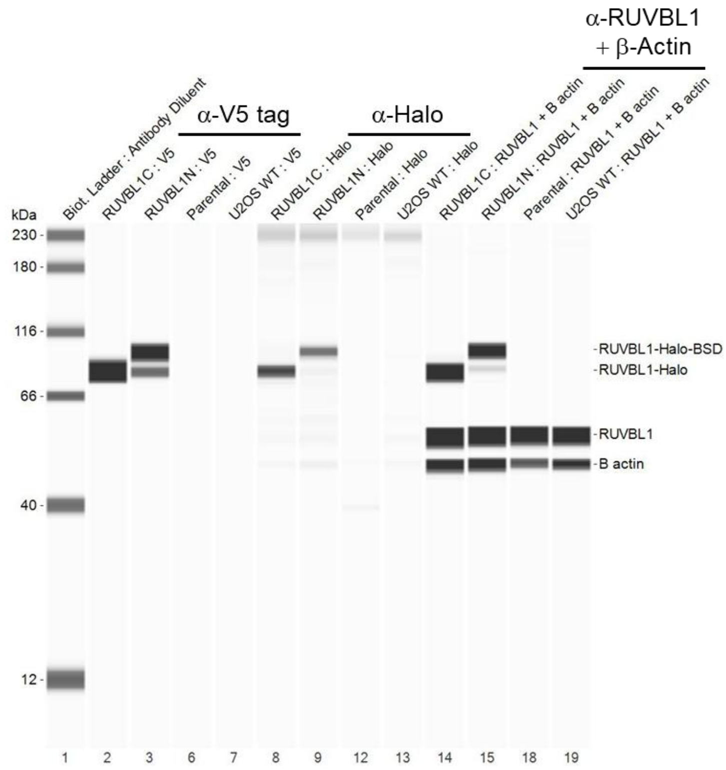

Figure S7. Western blot of Halo-N-RUVBL1, RUVBL1-C-Halo and parental cell-line controls. a) Cell-line validations were assessed with cell lysates from antibiotic-selected U2OS Halo-N-RUVBL1 and RUVBL1-C-Halo pools as well as parental landing pad and U2OS^{WT}. Primary antibody staining respectively probed for V5 tag, Halo tag, or multiplexed detection of RUVBL1 and β-Actin. Incomplete linker self-cleavage was observed for Halo-N-RUVBL1 with the presence of two Halo-tagged RUVBL1 variants containing or missing the

Blasticidin S Deaminase (BSD) antibiotic resistance marker. RUVBL1-C-Halo protein displays complete cleavage of the selection marker concordantly with the three primary antibodies used.

a)

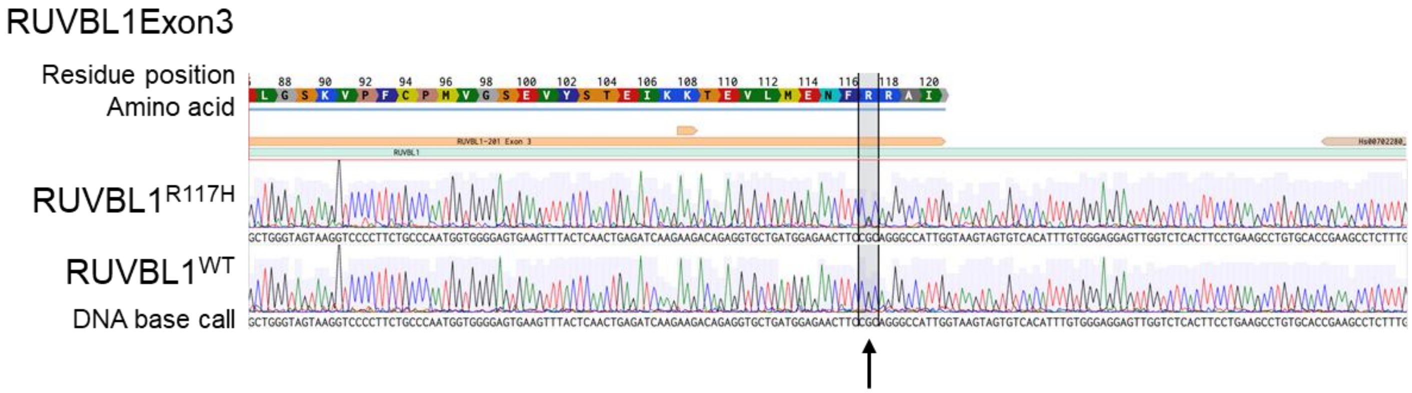

Figure S8. Genomic sequencing chromatograms of CB-6644 induced drug-resistant HCT116. a) Chromatograms of genomic DNA isolated from CB-6644 resistant HCT116 and DMSO-treated HCT116^{WT} and subsequently sequenced from PCR product amplifying the exon 3 region of RUVBL1. Arrow highlighting R117 position of RUVBL1, where a homozygous guanine (G) detected in HCT116WT, while a heterozygous adenosine (A) alongside the guanine is detected, resulting in a RUVBL$^{WT / R117H}$ mutant conferring resistance displayed by *in vitro* cell growth in the presence of CB-6644.

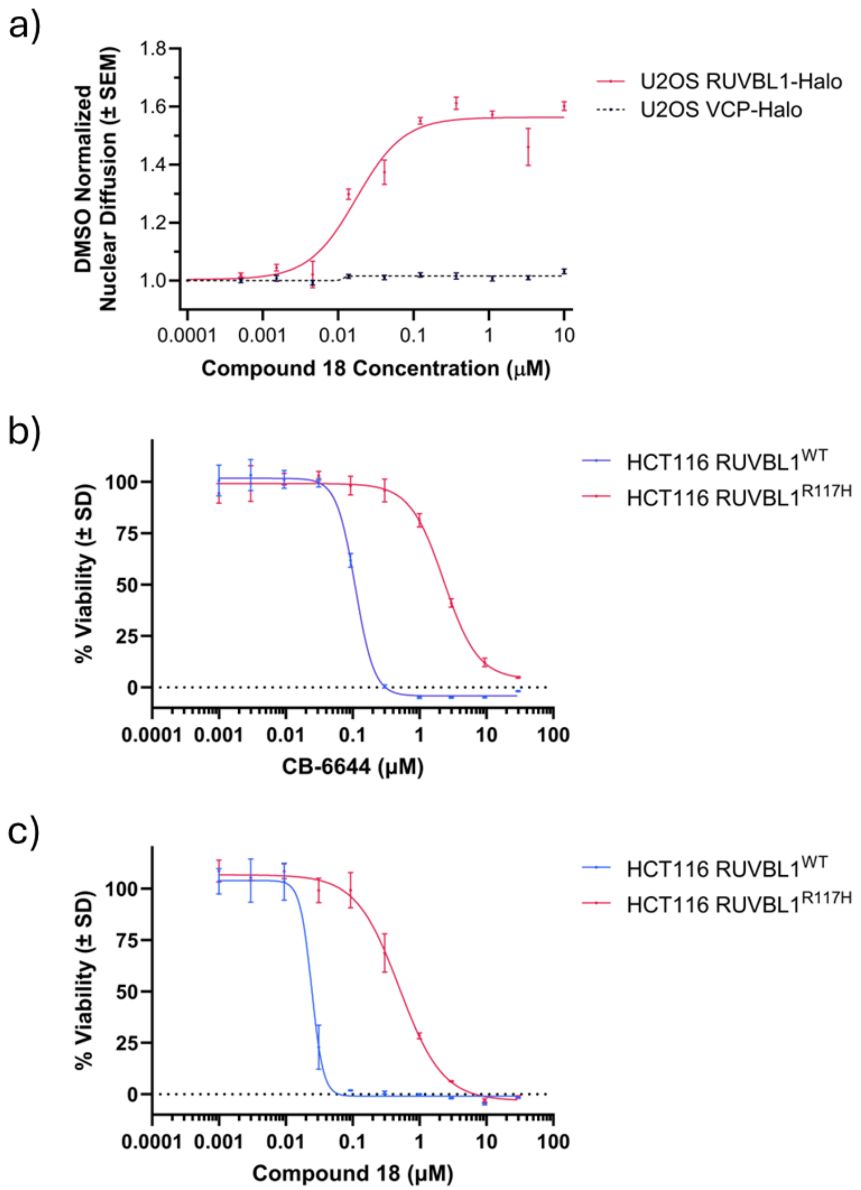

d)

	IC_{50} RUVBL1^{WT} (μM)	IC_{50} RUVBL1^{R117H} (μM)	Fold Change
CB-6644	0.110	2.32	**21.1**
Compound 18	0.0242	0.499	**20.6**

Figure S9. VCP ATPase counter-screening of compound 18 and potency comparison of CB-6644 alongside Compound 18 across both RUVBL1^{WT} and drug-induced RUVBL1^{R117H} mutant. a) SMT off-target assessment of Compound 18 in Halo-tagged RUVBL1 (n=48 per treatment datapoint) and VCP cell-lines (n=16 per treatment datapoint). b) CB-6644 IC_{50} curves generated in HCT-116 RUVBL1^{WT} and in CB-6644 induced RUVBL1^{R117H} cell-lines (n=3 per datapoint). c) Compound 18 IC_{50} curves generated in HCT-116 RUVBL1^{WT} and in CB-6644 induced RUVBL1^{R117H} cell-lines (n=3 per datapoint). d) Table summarizing fold-change in IC_{50} potency between RUVBL1^{R117H} and RUVBL1^{WT} for CB-6644 and compound 18.

a)

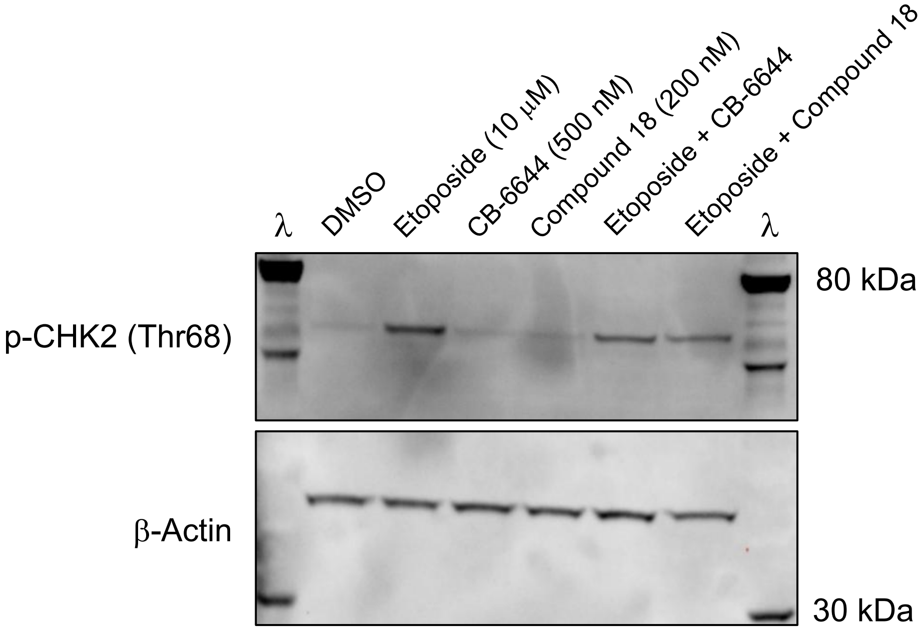

b)

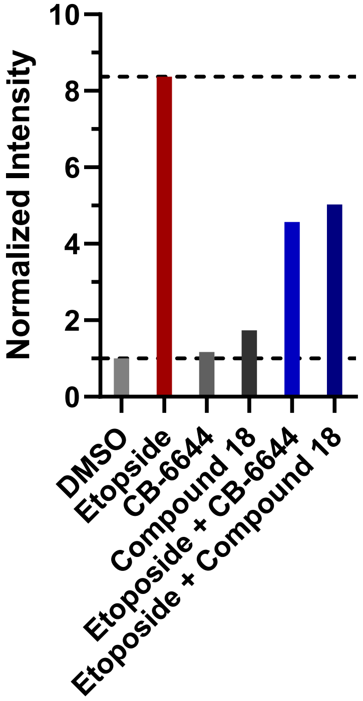

Figure S10. Compound 18 (at 200 nM) reduces etoposide induced p-CHK2 similarly to CB-6644 (at 500 nM). a) Western blot of p-CHK2 (Thr68; CST Cat2197) 1-hr post-treatment of respective compounds and combinations with etoposide (Life Technologies, iBright 1500). b) Quantification of normalized intensity of western blot bands (Life Technologies, iBright Analysis Software) normalizing respective bands to β-Actin controls (CST, Cat3700), and subsequent normalization to DMSO normalized p-CHK2 expression.

a)

b)

c)

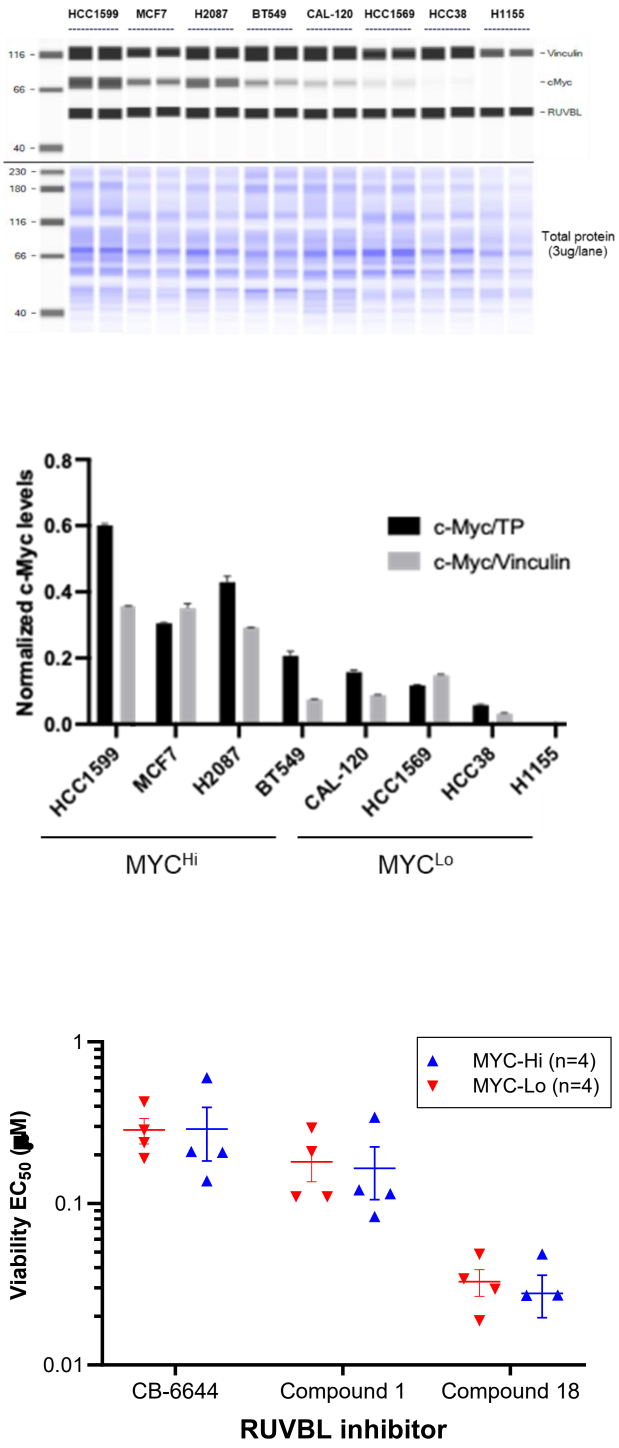

Figure S11. In vitro potency evaluation of RUVBL inhibitors in cell-lines with differential MYC expression. a) Western blot of 4 MYC^{Hi} and MYC^{Lo} cell-lines in addition to total protein quantification. b) Respective quantification of MYC protein levels (a) normalized to either vinculin or total protein levels (TP). c) Viability EC_{50} determination of RUVBL inhibitors CB-6644, compound 1 and 18 in cell-lines with differential MYC expression.

Materials for RUVBL Sequencing

RUVBL Sequencing Primers

RUVBL1

Life Tech Assay ID	Forward sequence	Reverse sequence	Expected Amplicon Size (bp)
N/A (Integrated DNA Tech)	TGAAGGGGTCTCATTGTCTCTAG	TTACCCTCGGCACCTAGAACAGT	433
Hs00239083_CE	GAAGTGGGTGACCGGAGCTCTA	CTGGCCCATTTTCACTTCCCTGAG	495
Hs00239081_CE	AGGGAAGATAAGAGATGCCATTTATGA	TTGCTAACGCACTTCAATGAGAAA	519
Hs00239080_CE	CCATCATGACACCCTGGAATGTA	AACATGCAGAGTTTCCTAGGAGTAA	521
Hs00419961_CE	TTCACACTCCTGCAGGTCCCA	TGGTTTCTCACCCTGTCCTCCC	525
Hs00239078_CE	GGCCTTTATCAGGAATTGCATACA	CAGCAAGACTCTGACAATTCATTCC	512
Hs00239077_CE	GGGAACCTCTAAAAATTCCTCTGTGT	AGAGCTGGGCGACACAAATCC	496
Hs00239076_CE	GAGGCTGCCAATTTTGTGACCTTTT	GGGTTTGGAGAGATCCTGAGGAA	497
Hs00239075_CE 9	AGTTGTTTTTATGCCACTGGAGGTT	AGGGAGCTGAGCTTCTGTTGAC	498
Hs00239074_CE 10	GAGGACATGGAGGCAAGAGTGA	AGGAAGGGACCTGCCTTTATTG	494
Hs00239073_CE	CCAGTGCTGCCTACTGATGTG	ACTTAGAGAGAGAGAGAGAGAGAATCA	505

RUVBL2

Life Tech Assay ID	Forward sequence	Reverse sequence	Expected Amplicon Size (bp)
Hs00187505_CE	CTTTGAAGAACCAGCTAGCCTAGA	AGCTTGGAACTCAAGTGCCATTG	507
Hs00187506_CE	ATCTCATGGGAGGGACAGAGGAG	GTACTGGGTATACGAGGCCAAACC	443
Hs00187507_CE	TCATTAGCCCTTGTGGTTAGATCTT	CATGTGGTTGAAGTTTATGGGTATGT	500
Hs00187508_CE	GCTTTGCCACCTTAAAAAAAAGTTAGA	CTTCCCTTCCCGGATCATCTCC	547
Hs00187509_CE	GGGAGAAAAGCTAGTTTGCCACAGA	GCAGACCTGATATCTACTCTCTCCAA	512
Hs00470023_CE	CATCGGCGTTCGCATCAAGT	AGGACATGCCTTTCGGCCAC	542
Hs00187510_CE	CCTTTGTCCCAGAGCTCATGGAA	TGTAAGATGGACAGGACATGCCTT	465
Hs00187513_CE	CTCCCCCAGACAGAGGGCTTTA	GGGAGCAAAGCAAGGAGGGATG	495
Hs00187514_CE	CCCTGGCATCATCCTTCTCTGT	GGTGTCAACACAGGCTCCCATT	493
Hs00187515_CE	AGATCAATGCCAAGGTGGCTGAGT	GGGCTAAAGGAGCAGCAAGACA	498
Hs00417920_CE	CAGCTGTGGAAGGCTGCGTC	CGGGTACCTCGCCTCCCTTT	491
Hs00187516_CE	ATGAAGAGGGAACATGCCCCTGA	GGAATATGTGGCGCCTGCATCT	494
Hs00187517_CE	CTACAGGAGAGAGGGCTGAGCG	CCCCTGAAGTACTGTGTCACTCA	508
Hs00187518_CE	GCTGCCTAGAGCATGGAGGTG	GGTGTCTTTCTGTGGGCTCAGG	493

Table S1. Sequencing primers used in targeted PCR amplification of exonic regions of RUVBL1 and RUVBL2, with base-pair (bp) length of expected amplicon.

Experimental Procedures for ADME assays

In vitro Caco-2 Permeability assay

Caco-2 cells obtained from the American Type Culture Collection were seeded (3.4 x 104 cells/insert) in a 96-well Transwell plate and cultivated for approximately 18 days. Transport buffer solution (HBSS, 10 mM HEPES, pH 7.4 with or without 1% BSA) containing 5 µM of test compounds, and either the control compounds digoxin (5 µM), atenolol (5 µM) or minoxidil (5 µM), was added to donor wells of the apical or basolateral plate. The transport buffer was added to appropriate receiving wells. Following incubation at 37°C for 2 hours, samples from both apical and basolateral sides were transferred into new 96-well plates and been precipitated by acetonitrile containing internal standard (100 nM alprazolam, 200 nM labetalol, 200 nM caffeine and 2 µM ketoprofen) prior to analysis by LC-MS/MS to determine the peak area of test compounds. Based on the apparent permeability (Papp) of substrates transport in the apical to basolateral direction and basolateral to apical direction of the cell monolayers, the efflux ratio was calculated. Lucifer yellow was used as a marker to confirm the integrity of the cell monolayers after 2 hours incubation.

Liver Microsome Stability assay

Incubations with 0.5 mg/mL male CD-1 mouse (Xenotech) and pooled gender human liver microsomes (Corning) suspended in 100 mM phosphate buffer at pH 7.4 were performed in duplicate with NADPH (1 mM) and UDPGA (2mM) or without co-factors. Test compounds were carried out at a final test concentration of 1 µM over a total incubation period of 60 minutes. Verapamil and 7-OH coumarin were used as positive controls. Samples were taken at 0 and 60 minutes and the reaction terminated by addition of acetonitrile containing internal standard (alprazolam (100 nM), labetalol (200 nM), caffeine (200 nM) and ketoprofen (2 µM)). The samples were analyzed by LCMS/MS to determine peak area of test compounds, which was used to calculate the percentage remaining.

Hepatocyte Stability assay

Incubations with 0.5×10^6 cells/mL male CD-1 mouse (TPCS) and pooled gender human hepatocytes (BioIVT) suspended in Williams' Medium E with 1× GlutaMAX were performed in duplicate. Test compounds were carried out at a final test concentration of 1 µM over a total incubation period of 60 minutes. Verapamil was used as positive control. Samples were taken at 0 and 60 minutes and the reaction terminated by addition of acetonitrile containing internal standard (alprazolam (100 nM), labetalol (200 nM), caffeine (200 nM) and ketoprofen (2 µM)). The samples were analyzed by LCMS/MS to determine peak area of test compounds, which was used to calculate the percentage remaining.

Plasma Protein Binding

Plasma protein binding was determined using equilibrium dialysis system. Load cells with spiked plasma sample and dialyzed against equal volume of dialysis buffer (PBS) at 37°C with 5% CO2 at approximately 150 rpm for 6 hours. Samples from both sides were processed with protein precipitation approach for LCMS/MS analysis. Unbound fraction was calculated by using peak area in buffer divided by peak area in plasma.

Kinetic Solubility Assay

The kinetic solubility of compounds in pH 7.4 phosphate-buffered saline (PBS) was evaluated at 37 °C and at two time points: 2 and 24 h. All samples were prepared in triplicate by addition of compounds as 10 mM DMSO stock to PBS buffer to arrive at diluted samples with a final residual DMSO concentration of 3% (v/v). The samples were incubated for 2 or 24 h at 1100 RPM and at 37 °C. After incubation, the samples were filtered, and the filtrates were collected. The filtrates were further diluted, using a 1:1 ratio of filtrate to DMSO to prepare the samples for analysis. The samples were analyzed via liquid-chromatography/mass spectrometry (LC/MS) to evaluate the molar quantity of solubilized compounds at the two time points (2 and 24 h), using the peak area to calculate the kinetic solubility value (Equation 1). The peak areas were evaluated at 254 nm. The kinetic solubility value was calculated by using the peak areas of the 2-h or 24-h sample (replicates 1) and the peak area of the standard sample (replicate 1). The same process was applied for the other 2-h or 24-h samples and standard samples (replicates 2 and 3, respectively), The kinetic solubility values were averaged (n=3) to estimate the final kinetic solubility value for each compound. The dilution factor, standard concentrations, and injection volumes can be varied, but the residual DMSO concentration was maintained at 3% (v/v).

Equation 1:

$$Kinetic\ Solubility = \frac{Peak\ Area_{Compound} \times Injection\ Volume_{Standard} \times Dilution\ Factor_{Compound} \times [Standard]}{Peak\ Area_{Standard} \times Injection\ Volume_{Compound}}$$

Experimental procedures for additional compounds

Reactions were stirred and heated (when applicable) using IKA RCT basic heating and stirring plates and IKA heating blocks monitored by thermocouple. Reactions were monitored by LC-MS or thin layer chromatography under short and long wave UV-irradiation. LC-MS data was collected on an Agilent 1260 LC-MS. Normal phase column chromatography was conducted on Teledyne ISCO Combiflash NextGen 300 or 300+ chromatography machines. 24 g RediSep® Basic alumina columns were purchased from Teledyne ISCO and were rated to have a particle size of 20-64 μm, mesh size of 230-400, pore size of 60 Å, surface area of 200 ± 50 m^2/g, pH of 9.7 ± 0.3, and loading capacity of 0.5 – 4%. Reverse phase HPLC was conducted on Teledyne ISCO AccqPrep HP 150 HPLC machines with a Kinetex® 5 μm XB-C18 100 Å, dimensions: 50 x 30 mm. All compounds assessed for *in vitro* and/or *in vivo* biological activity had a purity of 95% or above as estimated from their ^{1}H NMR spectra and their HPLC UV traces. All solvents used were commercially available and of analytical grade. Anhydrous solvents were routinely used for reactions. LC-MS data was collected on an Agilent 1260 LC-MS. ^{1}H NMR was performed on Bruker Ascend 400. Data for ^{1}H NMR are reported relative to residual solvent as calculated by MestreNova and are reported in the following format: ppm (multiplicity, coupling constant, integration). Solubility and permeability data were collected by Pharmaron, Inc (Beijing, China).

**Abbreviation**	**Full Name**
AcOH	acetic acid
CDI	1,1'-carbonyldiimidazole
$CDCl_3$	deuterochloroform (chloroform-d)
CH_2Cl_2	dichloromethane
CMPB	1-(cyanomethyl)piperazine
concd	concentrated
Cs_2CO_3	cesium carbonate

Abbreviation	Full Name
CsF	cesium fluoride
$Cu(OAc)_2$	copper(II) acetate
DAST	diethylaminosulfur trifluoride
DBU	1,8-diazabicyclo[5.4.0]undec-7-ene
DCE	1,2-dichloroethane
DIPEA	*N*,*N*-diisopropylethylamine
DMF	*N*,*N*-dimethylformamide
DMSO	dimethyl sulfoxide
EtOAc	ethyl acetate
EtOH	ethanol
H_2O	water
HATU	1-[Bis(dimethylamino)methylene]-1H-1,2,3-triazolo[4,5-b]pyridinium 3-oxid hexafluorophosphate
HCl	hydrochloric acid
K_2CO_3	potassium carbonate
KI	potassium iodide
KOAc	potassium acetate
KOH	potassium hydroxide
LC/MS	Liquid Chromatography-Mass Spectrometry
LHMDS	lithium bis(trimethylsilyl)amide
$LiOH \cdot H_2O$	lithium hydroxide monohydrate
MeCN	acetonitrile

**Abbreviation**	**Full Name**
MeOH	methanol
MTBE	methyl *tert*-butyl ether
Na_2SO_4	sodium sulfate
$NaBH_4$	sodium borohydride
NaH	sodium hydride
NaOH	sodium hydroxide
NBS	*N*-bromosuccinimide
NH_4Cl	ammonium chloride
NH_4HCO_3	ammonium formate
p-CHK2	phospho-Checkpoint Kinase 2
Pd/C	palladium on carbon
$Pd(OAc)_2$	palladium(II) acetate
PIKK	phosphatidylinositol 3-kinase-related kinase
rt	room temperature
TEA	triethylamine
THF	tetrahydrofuran

General procedure for preparation of 2-amino-3-methyl-5,6-dihydro-1H-benzo[b]pyrazolo[1,2-d][1,4,5] oxadiazepin-1-one (25).

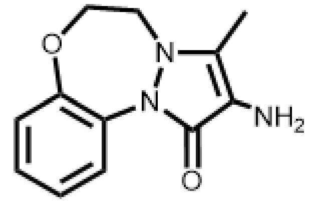

Step 1: Preparation of 1-bromo-2-(2,2-diethoxyethoxy)benzene (20).

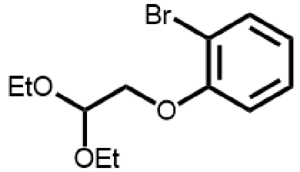

A solution of KOH (3.89 g, 69.5 mmol) in H_2O (6.70 mL) was added to 2-bromophenol **(19)** (10.0 g, 57.8 mmol) at rt. To the solution was added 2-bromo-1,1-diethoxy-ethane (17.1 g, 86.8 mmol) in DMSO (100 mL) at rt. The mixture was heated to 100 °C and stirred for 16 h before it was cooled to rt, poured into H_2O (200 mL) and extracted with MTBE (80 mL × 3). The combined organic layers were washed with 5% NaOH aqueous solution (80 mL), dried over Na_2SO_4, filtered and concentrated in vacuo to afford 16.0 g (96% yield) of 1-bromo-2-(2,2-diethoxyethoxy)benzene **(20)** as a colorless oil. ^1H NMR (400MHz, $CDCl_3$) δ 7.50 (dd, J=1.6, 8.0 Hz, 1H), 7.25-7.19 (m, 1H), 6.88 (dd, J=1.2, 8.4 Hz, 1H), 6.81 (dt, J=1.2, 7.6 Hz, 1H), 4.86 (t, J=5.2 Hz, 1H), 4.03 (d, J=5.2 Hz, 2H), 3.80-3.77 (m, 2H), 3.70-3.66 (m, 2H), 1.24 (t, J=7.2 Hz, 6H).

Step 2: Preparation of 2-(2-bromophenoxy)acetaldehyde (21).

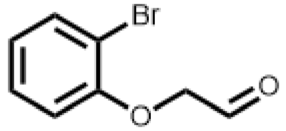

A mixture of 1-bromo-2-(2,2-diethoxyethoxy)benzene (**20**) (85.3 g, 294.9 mmol) in 1,4-dioxane (150 mL), H_2O (150 mL) and con. HCl (150 mL) was stirred at rt for 16 h. The aqueous phase was extracted with CH_2Cl_2 (500

mL × 2). The combined organic layers were washed with saturated $NaHCO_3$ (400 mL × 2) and brine (800 mL × 2), dried over Na_2SO_4, filtered and concentrated in vacuo to afford 52.0 g (82% yield) of 2-(2-bromophenoxy)acetaldehyde **(21)** as a light yellow oil. ^1H NMR (400 MHz, $CDCl_3$) δ 9.91 (t, J=0.9 Hz, 1H), 7.60 (dd, J=7.6 Hz, 1.6 Hz, 1H), 7.51-7.57 (m, 1H), 7.25-7.28 (m, 1H), 6.80 (dd, J=8.4 Hz, 0.9 Hz, 1H), 4.63 (d, J=0.9 Hz, 2H). LCMS (ESI): m/z = 214.88 $[M+H]^+$.

Step 3: Preparation of 1-(2-(2-bromophenoxy)ethyl)-5-methylpyrazolidin-3-one (22).

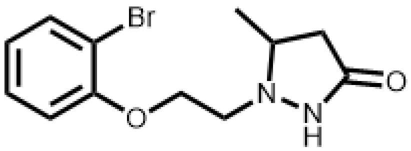

A mixture of 5-methylpyrazolidin-3-one (24.3 g, 243.0 mmol) and 2-(2-bromophenoxy)acetaldehyde (**21**) (52.0 g, 243.0 mmol) in anhydrous MeOH (250 mL) was stirred at rt for 16 h. Then $NaBH_4$ (13.8 g, 364.6 mmol) was added to the mixture at 0 °C. The mixture was warmed to rt and stirred for 1 h before the addition of H_2O (300 mL). The mixture was concentrated in vacuo to remove the MeOH. The aqueous phase was extracted with EtOAc (300 mL × 3). The combined organic layers were washed with brine (500 mL × 2), dried over Na_2SO_4, filtered and concentrated in vacuo. The residue was purified by silica gel column chromatography (eluent: 25-50% EtOAc in petroleum ether) to afford 42.2 g (58% yield) of 1-(2-(2-bromophenoxy)ethyl)-5-methylpyrazolidin-3-one (**22**) as a colorless oil. ^1H NMR (400 MHz, $CDCl_3$) δ 7.98 (br s, 1H), 7.51-7.58 (m, 1H), 7.23-7.30 (m, 1H), 6.83-6.91 (m, 2H), 4.17-4.24 (m, 2H), 3.26-3.35 (m, 1H), 3.15-3.22 (m, 1H), 3.05-3.14 (m, 1H), 2.70 (dd, J=16.4 Hz, 7.6 Hz, 1H), 2.17 (dd, J=16.4 Hz, 9.2 Hz, 1H), 1.27-1.32 (m, 3H). LCMS (ESI): m/z = 298.97 $[M+H]^+$.

Step 4: Preparation of 3-methyl-2,3,5,6-tetrahydro-1H-benzo[b]pyrazolo[1,2-d][1,4,5]oxadiazepin-1-one (23).

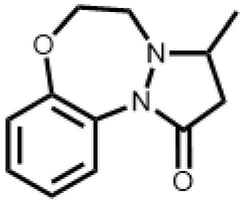

A mixture of 1-(2-(2-bromophenoxy)ethyl)-5-methylpyrazolidin-3-one (**22**) (34.1 g, 114.0 mmol), CuI (10.9 g, 57.0 mmol), 1,10-phenanthroline (5.14 g, 28.5 mmol) and Cs_2CO_3 (74.3 g, 228 mmol) in anhydrous 1,4-dioxane (350 mL) was stirred at 120 °C for 16 h. The mixture was filtered through a pad of celite, the filter cake was washed with CH_2Cl_2 (50 mL × 3). The filtrate was concentrated in vacuo to remove all solvent. The residue was partitioned between H_2O (500 mL) and CH_2Cl_2 (500 mL). The aqueous layer was extracted with CH_2Cl_2 (200 mL × 2). The combined organic layers were washed with brine (5 mL × 2), dried over Na_2SO_4, filtered and concentrated in vacuo. The residue was purified by silica gel column chromatography (eluent: 20-55% EtOAc in petroleum ether) to afford 12.2 g (49% yield) of 3-methyl-2,3,5,6-tetrahydro-1H-benzo[b]pyrazolo[1,2-d][1,4,5] oxadiazepin-1-one (**23**) as a brown solid. ^{1}H NMR (400 MHz, $CDCl_3$) δ 7.47 (dd, J=8.0 Hz, 1.6 Hz, 1H), 7.18-7.24 (m, 1H), 7.06-7.13 (m, 2H), 4.37 (d, J=12.0 Hz, 1H), 3.95-4.06 (m, 1H), 3.52-3.50 (m, 1H), 3.36-3.45 (m, 1H), 3.13-3.19 (m, 2H), 2.22-2.33 (m, 1H), 1.35 (d, J=6.84 Hz, 3H). LCMS (ESI): m/z = 219.1 $[M+H]^+$.

Step 5: Preparation of 2-bromo-3-methyl-2,3,5,6-tetrahydro-1H-benzo[b]pyrazolo[1,2-d][1,4,5]oxadiazepin-1-one (S1).

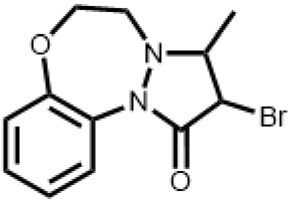

To a mixture of 3-methyl-2,3,5,6-tetrahydro-1H-benzo[b]pyrazolo[1,2-d][1,4,5]oxadiazepin-1-one (23) (12.2 g, 55.9 mmol) in anhydrous THF (250 mL) was added 1M LHMDS in THF (123 mL, 0.123 mmol) dropwise at -78 °C under N2. The mixture was stirred at -78 °C for 5 min before the dropwise addition of a mixture of NBS (9.95 g, 55.9 mmol) in anhydrous THF (150 mL) at -78 °C. The mixture was stirred at -78 °C for 5 m. Saturated NH4Cl (200 mL) was then added and the resulting solution was warmed to rt and extracted with EtOAc (1 L × 2). The combined organic layers were washed with brine (500 mL × 2), dried over Na2SO4, filtered and concentrated down in vacuo to afford 16.9 g (crude material) of 2-bromo-3-methyl-2,3,5,6-tetrahydro-1H-benzo[b]pyrazolo[1,2-d][1,4,5]oxadiazepin-1-one (**S1**) as a brown oil. This material was used directly in the synthesis of **24** without further purification. LCMS (ESI): m/z = 297.1 $[M+H]^+$.

Step 6: Preparation of 3-methyl-5,6-dihydro-1H-benzo[b]pyrazolo[1,2-d][1,4,5]oxadiazepin-1-one (24).

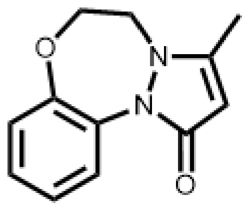

A mixture of crude 2-bromo-3-methyl-2,3,5,6-tetrahydro-1H-benzo[b]pyrazolo[1,2-d][1,4,5]oxadiazepin-1-one **(S1)** (16.9 g crude, 55.90 mmol) and DBU (8.84 mL, 56.94 mmol) in 1,4-dioxane (160 mL) was stirred at 80 °C for 1 h before cooling to rt and quenching with H_2O (50 mL). The mixture was concentrated in vacuo to remove the 1,4-dioxane. The mixture was dilute with H_2O (200 mL) and extracted with CH_2Cl_2 (300 mL × 3). The combined organic layers were dried over Na_2SO_4, filtered and concentrated in vacuo. The residue was purified by silica gel column chromatography (eluent: 0-50% EtOAc in petroleum ether) to afford 6.52 g (54% yield, over two steps) of 3-methyl-5,6-dihydro-1H-benzo[b]pyrazolo[1,2-d][1,4,5]oxadiazepin-1-one (**24**) as a yellow solid. ^{1}H NMR (400 MHz, $CDCl_3$) δ 7.83 (dd, J=8.0 Hz, 2.0 Hz, 1H), 7.23-7.27 (m, 1H), 7.21-7.23 (m, 1H), 7.13 (dd, J=8.0 Hz, 2.0 Hz, 1H), 5.46 (s, 1H), 4.27-4.32 (m, 2H), 3.87-3.93 (m, 2H), 2.23 (s, 3H). LCMS (ESI): m/z = 217.1 $[M+H]^+$.

Step 7: Preparation of 3-methyl-2-nitro-5,6-dihydro-1H-benzo[b]pyrazolo[1,2-d][1,4,5]oxadiazepin-1-one (S2).

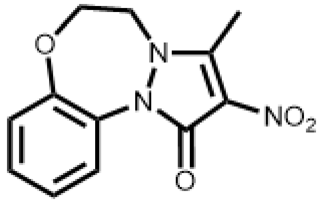

To a mixture of 3-methyl-5,6-dihydro-1H-benzo[b]pyrazolo[1,2-d][1,4,5]oxadiazepin-1-one **(24)** (6.60 g, 30.5 mmol) in TFA (30 mL) was added HNO_3 (68%, 2 mL, 30.5 mmol) dropwise at 0 °C. The mixture was stirred at rt for 1 h before it was poured into ice-cold H_2O (100 mL). The mixture was filtered, and the residue was dried in vacuo to afford 7.81 g (98% yield) of 3-methyl-2-nitro-5,6-dihydro-1H-benzo[b]pyrazolo[1,2-d][1,4,5]oxadiazepin-1-one (**S2**) as a yellow solid This material was used in the following step without any further purification. ^{1}H NMR (400 MHz, $CDCl_3$) δ 7.77 (dd, J=8.0 Hz, 2.0 Hz, 1H), 7.30-7.41 (m, 1H), 7.30-7.41 (m, 1H), 7.22 (dd, J=8.0 Hz, 1.6 Hz, 1H), 4.38-4.45 (m, 2H), 4.26-4.34 (m, 2H), 2.80 (s, 3H). LCMS (ESI): m/z = 262.2 $[M+H]^+$.

Step 8: Preparation of 2-amino-3-methyl-5,6-dihydro-1H-benzo[b]pyrazolo[1,2-d][1,4,5] oxadiazepin-1-one (25).

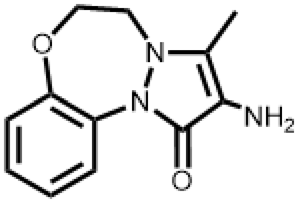

3-methyl-2-nitro-5,6-dihydro-1H-benzo[b]pyrazolo[1,2-d][1,4,5]oxadiazepin-1-one **(S2)** (7.81 g, 29.90 mmol), was suspended in EtOH/H_2O (2:1) (90 mL) and DCE (3 mL). Following which Fe powder (8.52g, 153 mmol) and NH_4Cl (1.60 g, 30.5 mmol) were added, the resulting mixture was heated to 80 °C and stirred for 1 h. The

reaction mixture was cooled to rt and filtered through a pad of celite. Filter cake was washed with CH_2Cl_2 (50 mL). Filtrate was concentrated in vacuo to remove organic solvents, the resulting solution was extracted with CH_2Cl_2 (100 mL × 2). The combined organic layers were dried over Na_2SO_4, filtered and concentrated in vacuo to afford 5.10 g (72% yield two steps) of 2-amino-3-methyl-5,6-dihydro-1H-benzo[b]pyrazolo[1,2-d][1,4,5]oxadiazepin-1-one (**25**) as a brown solid. 1H NMR (400 MHz, DMSO-d_6): δ 9.58 (br s, 2H), 7.65 (dd, J = 8.0 Hz, 1.6 Hz, 1H), 7.39-7.35 (m, 1H), 7.29-7.22 (m, 2H), 4.32 (t, J = 5.2 Hz, 2H), 4.13 (t, J = 5.2 Hz, 2H), 2.36 (s, 3H). LCMS (ESI): m/z = 232.2 $[M+H]^+$.

Preparation of 3-bromo-4-(2,2-diethoxyethoxy)pyridine (S3).

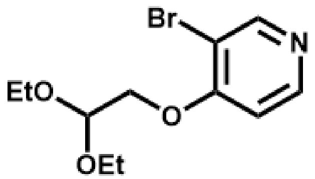

To a mixture of 2,2-diethoxy ethanol (19.2 g, 143 mmol) in DMF (150 mL) was added NaH (60%, 5.71 g, 143 mmol) at 0 °C. The mixture was stirred at 0 °C for 30 m. Then 3-bromo-4-chloro-pyridine (25.0 g, 130 mmol) was added to the mixture. The mixture was warmed to rt and stirred for 16 h under N_2. The reaction was quenched by addition of H_2O (200 mL) the reaction mixture was extracted with EtOAc (200 mL × 3). The combined organic layers were dried over Na_2SO_4, filtered and concentrated in vacuo to afford 36.9 g (98% yield) of 3-bromo-4-(2,2-diethoxyethoxy)pyridine (**S3**) as a colorless solid. ^1H NMR (400MHz, $CDCl_3$) δ 8.58 (s, 1H), 8.38 (d, J=5.6 Hz, 1H), 6.82 (d, J=5.6 Hz, 1H), 4.87 (t, J=5.6 Hz, 1H), 4.10 (d, J=5.2 Hz, 2H), 3.81 (q, J=7.2 Hz, 2H), 3.68 (q, J=7.2 Hz, 2H), 1.25 (t, 6H). LCMS (ESI): m/z = 290.1 $[M+H]^+$.

Preparation of 10-amino-9-methyl-6,7-dihydro-11H-pyrazolo[1,2-d]pyrido[4,3-b][1,4,5]oxadiazepin-11-one (S4).

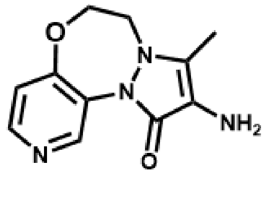

The title compound (**S4**) was prepared using a similar procedure as intermediate (**25**), steps 2-8, replacing 1-bromo-2-(2,2-diethoxyethoxy)benzene (**21**) with 3-bromo-4-(2,2-diethoxyethoxy)pyridine (**S3**) in step 2 to afford 30.0 mg (48% yield) of 10-amino-9-methyl-6H-pyrazolo[1,2-d]pyrido[4,3-b][1,4,5]oxadiazepin-11(7H)-one as a brown solid (**S4**). ^{1}H NMR (400MHz, MeOD-d_4) δ 8.71 (s, 1H), 8.38 (s, 1H), 7.20 (s, 1H), 4.41 (s, 2H), 3.94 (s, 2H), 2.21 (s, 3H) NH protons not observed. LCMS (ESI): m/z = 233.2 $[M+H]^+$.

Preparation of 2-amino-5,6-dihydro-1H-benzo[b]pyrazolo[1,2-d][1,4,5]oxadiazepin-1-one (S5).

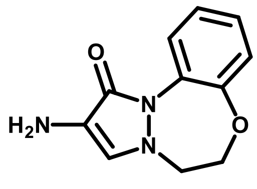

Title compound (**S5**) was prepared using a similar procedure as intermediate (**25**), step 1-8, replacing 5-methylpyrazolidin-3-one with pyrazolidin-3-one in step 3. In step 8, the product was purified by preparatory HPLC (eluent: 0-100% MeCN in H_2O) to afford 15.0 mg (21% yield) of 2-amino-5,6-dihydro-1H-benzo[b]pyrazolo[1,2-d][1,4,5]oxadiazepin-1-one (**S5**) as an off-white solid. ^{1}H NMR (400 MHz, $CDCl_3$) δ 7.68-7.56 (m, 1H), 7.27-7.19 (m, 1H), 7.16-7.09 (m, 2H), 6.89 (s, 1H), 4.43-4.39 (m, 4H), 3.47-3.36 (m, 2H). LCMS (ESI): m/z = 218.2 $[M+H]^+$.

Synthesis of 5,6-dihydrobenzo[f]pyrazolo[1,5-d][1,4]oxazepin-2-amine (S6).

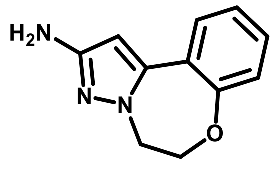

Step 1: Preparation of 1-bromo-2-(2-bromoethoxy) benzene (S7).

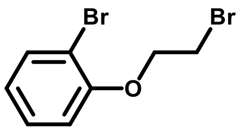

To a mixture of 2-bromophenol (**19**) (5.00 g, 28.9 mmol) in MeCN (100 mL) was added K_2CO_3 (7.99 g, 57.8 mmol) and 1,2-dibromoethane (12.5 mL, 145 mmol) at rt. The reaction mixture was heated to 80 °C and stirred for 16 h. The mixture was cooled to rt and dilute with EtOAc (100 mL) and washed with H_2O (100 mL × 2). The combined organic layers were dried over Na_2SO_4, filtered and concentrated in vacuo. The residue was purified by silica gel column chromatography (eluent: 100% petroleum ether) to afford 3.96 g (49% yield) of 1-bromo-2-(2-bromoethoxy)benzene (**S7**) as a colorless oil.

Step 2: Preparation of 1-(2-(2-bromophenoxy) ethyl)-3-nitro-1H-pyrazole (S8).

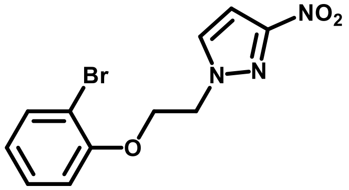

To a mixture of 1-bromo-2-(2-bromoethoxy)benzene (**S7**) (1.00 g, 3.57 mmol) in MeCN (15 mL) were added Cs_2CO_3 (1.75 g, 5.36 mmol) and 3-nitro-1H-pyrazole (0.4 g, 3.54 mmol) at rt. The reaction mixture was heated to 80 °C and stirred for 16 h. Upon completion, the mixture was cooled to rt, dilute with H_2O (50 mL) and extracted with EtOAc (50 mL × 3). The combined organic layers were dried over Na_2SO_4, filtered and concentrated in vacuo. The residue was purified by silica gel column chromatography (eluent: 0-20% EtOAc in petroleum ether) to afford 0.65 g (58% yield) of 1-(2-(2-bromophenoxy) ethyl)-3-nitro-1H-pyrazole (**S8**) as a yellow oil.

Step 3: Preparation of 2-nitro-5,6-dihydrobenzo[f]pyrazole [1,5-d][1,4]oxazepane (S9).

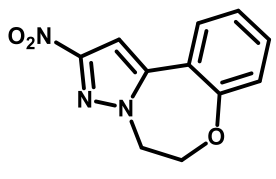

To a mixture of 1-(2-(2-bromophenoxy)ethyl)-3-nitro-1H-pyrazole (**S8**) (1.20 g, 3.84 mmol) in DMF (12 mL) was added KOAc (0.380 g, 3.84 mmol), tricyclohexylphosphine tetrafluoroborate (0.141 g, 0.384 mmol) and

$Pd(OAc)_2$ (0.086 g, 0.384 mmol). The resulting mixture was heated to 140 °C and stirred for 16 h. The mixture was cooled to rt, dilute with H_2O (100 mL) and extracted with EtOAc (100 mL × 3). The combined organic layers were dried over Na_2SO_4, filtered and concentrated in vacuo. The residue was purified by silica gel column chromatography (eluent: 0-100% EtOAc in petroleum ether) to afford 0.20 g (22% yield) of 2-nitro-5,6-dihydrobenzo[f]pyrazole [1,5-d][1,4]oxazepine (**S9**) as an off-white solid.

Step 4: Preparation of 5,6-dihydrobenzo[f]pyrazolo[1,5-d][1,4]oxazepin-2-amine (S6).

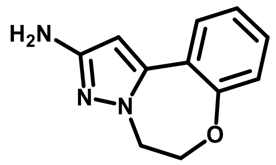

To a mixture of 2-nitro-5,6-dihydrobenzo[f]pyrazolo[1,5-d][1,4]oxazepane (**S9**) (0.500 g, 2.16 mmol) in MeOH (50 mL) was added 10% Pd/C (50% wet, 0.500 g). The reaction mixture was stirred under an atmosphere of H_2 using a balloon at rt for 3 h before filtering through a pad of celite, the filter cake was washed with MeOH (50 mL). The filtrate was concentrated under reduced pressure and triturated with Et_2O to afford 0.344 g (79% yield) of 5,6-dihydrobenzo[f]pyrazolo[1,5-d][1,4]oxazepin-2-amine (**S6**) as an off-white solid. ^1H NMR (400 MHz, DMSO-d_6): δ 7.70 (dd, J = 8.0 Hz, 1.6 Hz, 1H), 7.24-7.20 (m, 1H), 7.08-7.04 (m, 1H), 7.00 (dd, J = 8.4 Hz, 1.2 Hz, 1H), 5.94 (s, 1H), 4.64 (s, 1H), 4.37-4.32 (m, 4H), one NH is not located. LCMS (ESI): m/z = 202.2[M+H]$^+$.

Preparation of 2-amino-3-methyl-5,6-dihydro-1H-benzo[b]pyrazolo[1,2-d][1,4,5]oxadiazepin-1-one (S10).

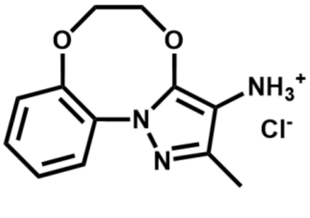

Step-1: Preparation of 2-(2-methoxyphenyl)-5-methyl-1,2-dihydro-3H-pyrazol-3-one (S11).

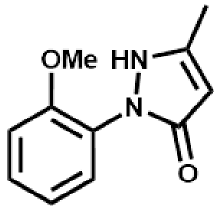

To the mixture of (2-methoxyphenyl) hydrazine hydrogen chloride (25.0 g, 143 mmol) in EtOH (200 mL) and AcOH (50 mL) was added ethyl 3-oxobutanoate (14.7 mL, 115 mmol) at rt, the reaction was then heated to 115 °C and stirred for 2 h. The reaction mixture was cooled to rt and concentrated in vacuo to remove organic solvent. The residue was purified by reverse phase column chromatography (eluent: 0-100% MeCN with 0.1% formic acid in H_2O) to afford 21.5g (91% yield) 2-(2-methoxyphenyl)-5-methyl-1,2-dihydro-3H-pyrazol-3-one (**S11**) as an off white solid. LCMS (ESI): m/z = 205.22 $[M+H]^+$.

Step-2: Preparation of 1-(2-hydroxyethyl)-2-(2-methoxyphenyl)-5-methyl-1,2-dihydro-3H-pyrazol-3-one (S12).

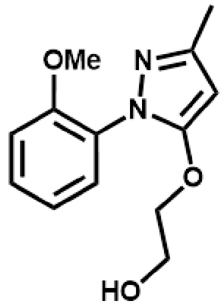

To a solution of 2-(2-methoxyphenyl)-5-methyl-1,2-dihydro-3H-pyrazol-3-one (**S11**) (10.6 g, 51.9 mmol) in DMF (100 mL) was added K_2CO_3 (28.7 g, 208 mmol), KI (0.862 g, 5.19 mmol) and 2-bromoethan-1-ol (5.50 mL, 77.9 mmol) at rt, the mixture was heated to 50 °C and stirred for 4 h. The reaction mixture was cooled to rt, quenched by pouring into ice-cold H_2O (1000 mL) and extracted with EtOAc (300 mL × 3). Combined organic layers were dried over Na_2SO_4 and concentrated in vacuo. The residue was purified by reverse phase column chromatography (eluent: 0-100% MeCN with 0.1% formic acid in H_2O) to afford 11.0 g (85% yield) of 1-(2-hydroxyethyl)-2-(2-methoxyphenyl)-5-methyl-1,2-dihydro-3H-pyrazol-3-one (**S12**) as a brown gum. LCMS (ESI): m/z = 249.14 $[M+H]^+$.

Step-3: Preparation of 1-(2-hydroxyethyl)-2-(2-hydroxyphenyl)-5-methyl-1,2-dihydro-3H-pyrazol-3-one

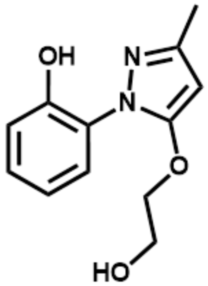

(S13). To a solution of 1-(2-hydroxyethyl)-2-(2-methoxyphenyl)-5-methyl-1,2-dihydro-3H-pyrazol-3-one (**S12**) (11.0 g, 44.3 mmol) in CH_2Cl_2 (110 mL) was added boron tribromide 1M in CH_2Cl_2 (443 mL, 443 mmol) drop wise at 0 °C, the solution was warmed to rt and stirred for 3 h. The reaction mixture was cooled to 0 °C followed by quenching with dropwise addition of MeOH (100 mL), the mixture was warmed to rt and concentrated in vacuo. The residue was dissolved in CH_2Cl_2 (250 mL) and adjusted to pH = 7 with saturated $NaHCO_3$ solution. The organic layer was collected and dried over Na_2SO_4, filtered and concentrate in vacuo to afford 10.0 g (96% yield) of 1-(2-hydroxyethyl)-2-(2-hydroxyphenyl)-5-methyl-1,2-dihydro-3H-pyrazol-3-one (**S13**) as a brown gum. LCMS (ESI): m/z = 235.15 $[M+H]^+$.

Step-4: Preparation of 3-methyl-5,6-dihydro-1H-benzo[b]pyrazolo[1,2-d][1,4,5]oxadiazepin-1-one (S14).

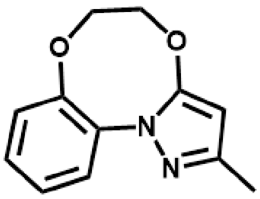

To a solution of 1-(2-hydroxyethyl)-2-(2-hydroxyphenyl)-5-methyl-1,2-dihydro-3H-pyrazol-3-one (**S13**) (5.00 g, 21.3 mmol) in toluene (50 mL) was added CMPB (10.3 g, 42.7 mmol) at rt. The reaction was heated to 110-115 °C and stirred for 6 h. The reaction mixture was then cooled to rt and concentrated in vacuo. The residue was purified by silica gel column chromatography (eluent: 0-20% EtOAc in petroleum ether) to afford 2.0 g (43% yield) of 3-methyl-5,6-dihydro-1H-benzo[b]pyrazolo[1,2-d][1,4,5]oxadiazepin-1-one (**S14**) as a brown gum. LCMS (ESI): m/z = 217.13 $[M+H]^+$.

Step-5: Preparation of 3-methyl-2-nitro-5,6-dihydro-1H-benzo[b]pyrazolo[1,2-d][1,4,5]oxadiazepin-1-one (S15).

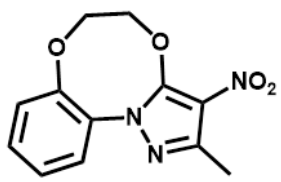

To a solution of 3-methyl-5,6-dihydro-1H-benzo[b]pyrazolo[1,2-d][1,4,5]oxadiazepin-1-one (**S14**) (1.10 g, 5.09 mmol) in MeCN (11 mL) in a sealed tube was added HNO_3 (65%, 11.0 mL, 264 mmol) at rt, the reaction was then warmed to 70 °C and stirred for 16 h. The reaction mixture was quenched by directly pouring on to crushed ice, the resulting mixture was dilute with H_2O (100 mL) and extracted with EtOAc (2 × 100 mL). The combined the organic layers were neutralized with saturated $NaHCO_3$ solution until frothing stop, the organic layer was collected and dried with Na_2SO_4, filtered and concentrated in vacuo. The residue was purified by silica gel column chromatography (eluent: 0-20% EtOAc in petroleum ether) to afford 0.50 g (38% yield) of 3-methyl-2-nitro-5,6-dihydro-1H-benzo[b]pyrazolo[1,2-d][1,4,5]oxadiazepin-1-one (**S15**) as a pale yellow solid. LCMS (ESI): m/z = 262.17 $[M+H]^+$.

Step-6: Preparation of 2-amino-3-methyl-5,6-dihydro-1H-benzo[b]pyrazolo[1,2-d][1,4,5]oxadiazepin-1-one (S10).

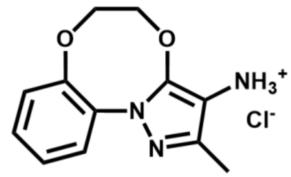

To a solution of 3-methyl-2-nitro-5,6-dihydro-1H-benzo[b]pyrazolo[1,2-d][1,4,5]oxadiazepin-1-one (**S15**) (1.30 g, 4.98 mmol) in MeOH (130 mL) and concd HCl (2.6 mL) was added 10% Pd/C (50% wet, 1.30 g), an atmosphere of H_2 was applied by balloon at rt and stirred for 3 h. The reaction mixture filtered through celite and filter cake washed with MeOH (100 mL). The filtrate was concentrated in vacuo. The residue was purified

by trituration with Et_2O (2 × 50 mL) to afford 1.27 g (95% yield) of 2-amino-3-methyl-5,6-dihydro-1H-benzo[b]pyrazolo[1,2-d][1,4,5]oxadiazepin-1-one (**S10**) as a light brown solid. 1H NMR (400 MHz, DMSO-*d_6*): δ 10.06 (br s, 3H), 7.49-7.45 (m, 2H), 7.36-7.30 (m, 2H), 4.32 (d, J = 4.4 Hz, 4H), 2.68 (s, 3H). LCMS (ESI): m/z = 232.20 $[M+H]^+$.

Preparation of ethyl 4-amino-3,3-dimethylbutanoate (31).

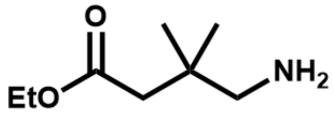

Step 1: Preparation of ethyl 3,3-dimethyl-4-nitrobutanoate (S16).

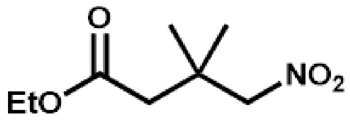

To a mixture of ethyl 3-methylbut-2-enoate (**30**) (100 g, 781 mmol) in MeCN (500 mL) was added nitromethane (209 mL, 3904 mmol) and DBU (1745 mL, 1171 mmol). The resulting solution was heated to 80 °C and stirred for 56 h. The reaction mixture was then cooled to rt and poured into H_2O (300 mL). The mixture was then extracted with EtOAc (200 mL × 3). The combined organic layers were dried over Na_2SO_4, filtered and concentrated in vacuo. The residue was purified by silica gel column chromatography (eluent: 10-20% EtOAc in petroleum ether) to afford 134 g (91% yield) of ethyl 3,3-dimethyl-4-nitro-butanoate (**S16**) as a colorless oil. ^{1}H NMR (400MHz, $CDCl_3$) δ 4.49 (s, 2H), 4.10 (q, *J*=7.2 Hz, 2H), 2.40 (s, 2H), 1.22 (t, *J*=7.2 Hz, 3H), 1.12 (s, 6H). LCMS (ESI): m/z = 190.2 $[M+H]^+$.

Step 2: Preparation of ethyl 4-amino-3,3-dimethylbutanoate (31).

To a mixture of ethyl 3,3-dimethyl-4-nitro-butanoate (**S16**) (30.0 g, 159 mmol) in EtOH (600 mL) was added 10% Pd/C (10 g). The mixture was heated to 50 °C and stirred for 48 h under H_2 (50 psi). The mixture was then filtered, and the filter cake washed with EtOH (200 mL). The filtrate was concentrated to afford 19.9 g (79% yield) of ethyl 4-amino-3,3-dimethylbutanoate (**31**) as a colorless oil. ^{1}H NMR (400MHz, $CDCl_3$) δ 8.15 - 8.05 (m, 2H), 4.12 - 4.01 (q, J=7.0 Hz, 2H), 3.67 (q, J=7.0 Hz, 2H), 2.40 (s, 2H), 1.18 (t, J=7.0 Hz, 3H), 1.10 (s, 6H).

Preparation of 4-(5-chloro-2-ethoxy-4-fluorobenzamido)-3,3-dimethylbutanoic acid (32).

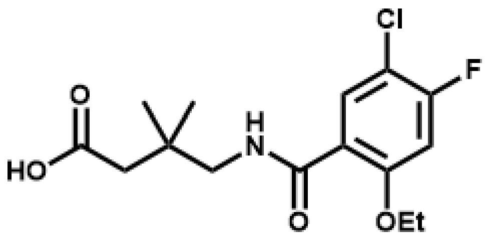

Step 1: Preparation of ethyl 4-(5-chloro-2-ethoxy-4-fluorobenzamido)-3,3-dimethylbutanoate (S17).

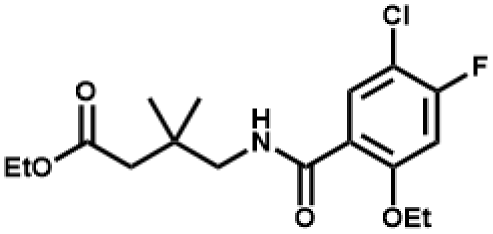

To a mixture of 5-chloro-2-ethoxy-4-fluorobenzoic acid (**27**) (10.0 g, 45.7 mmol) in THF (100 mL) was added HATU (26.1 g, 68.6 mmol). The solution was stirred at rt for 30 m. Followed by the addition of ethyl 4-amino-3,3-dimethyl-butanoate (**31**) (8.01 g, 50.3 mmol) and TEA (19.1 mL, 137 mmol). The reaction mixture was stirred at rt for 16 h. The mixture was poured into H_2O (100 mL) and extracted with EtOAc (80 mL × 3). The combined organic layers were dried over Na_2SO_4, filtered and concentrated in vacuo. The residue was purified by silica gel column chromatography (eluent: 5-10% EtOAc in petroleum ether) to afford 7.88 g (48% yield) of ethyl 4-[(5-chloro-2-ethoxy-4-fluoro-benzoyl)amino]-3,3-dimethyl-butanoate (**S17**) as a colorless oil.
^{1}H NMR (400MHz, $CDCl_3$) δ 8.22 (d, J=8.8 Hz, 1H), 8.04 - 7.95 (t, J=5.6 Hz, 1 H), 6.74 (d, J=10.6 Hz, 1H), 4.12 (m, J=7.2, 18.7 Hz, 4H), 3.38 (d, J=6.4 Hz, 2H), 2.24 (s, 2H), 1.50 (t, J=6.8 Hz, 3H), 1.26 - 1.18 (t, J=7.2, 3H), 1.03 (s, 6H). LCMS (ESI): m/z = 360.1 $[M+H]^+$.

Step 2: Preparation of 4-(5-chloro-2-ethoxy-4-fluorobenzamido)-3,3-dimethylbutanoic acid (32).

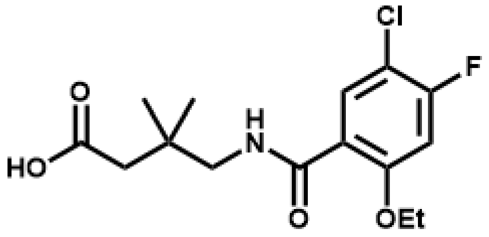

To a mixture of ethyl 4-[(5-chloro-2-ethoxy-4-fluorobenzoyl)amino]-3,3-dimethyl-butanoate (**S17**) (7.88 g, 21.9 mmol) in THF (150 mL) and H_2O (50 mL) was added $LiOH{\cdot}H_2O$ (9.21 g, 219 mmol). The mixture was stirred at rt for 48 h. Upon completion, the mixture was poured into H_2O (100 mL) and adjusted to pH = 2-3 by concd HCl. The mixture was filtered, the residue was washed with H_2O (200 mL) and dried in vacuo to afford 4.87 g (67% yield) of 4-(5-chloro-2-ethoxy-4-fluorobenzamido)-3,3-dimethylbutanoic acid (**32**) as a pink solid. ^{1}H NMR (400MHz, DMSO-d_6) δ 12.85 - 11.68 (s, 1H), 8.21 - 8.07 (t, J=8.9 Hz, 1H), 7.83 (d, J=8.9 Hz, 1H), 7.29 (d, J=11.5 Hz, 1H), 4.17 (d, J=6.9 Hz, 2H), 3.26 (d, J=6.3 Hz, 2H), 2.17 (s, 2H), 1.39 (t, J=7.0 Hz, 3H), 0.98 (s, 6H). LCMS (ESI): m/z = 332.1 $[M+H]^+$.

Preparation of 4-(5-chloro-2-methylbenzofuran-7-carboxamido)-3,3-dimethylbutanoic acid (33).

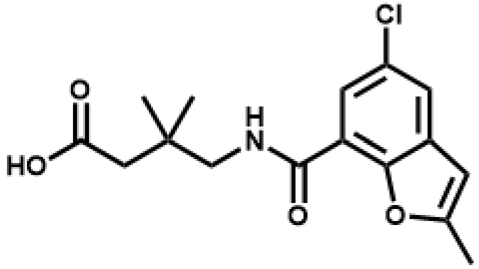

Step 1: Preparation of ethyl 4-(5-chloro-2-methylbenzofuran-7-carboxamido)-3,3-dimethylbutanoate (S18).

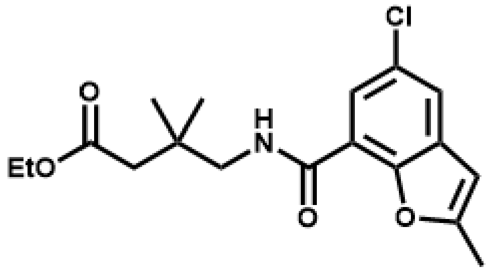

To a mixture of 5-chloro-2-methyl-benzofuran-7-carboxylic acid (**29**) (0.90 g, 4.28 mmol) in THF (10 mL) was added HATU (2.44 g, 6.41 mmol). The mixture was stirred at rt for 30 m. Followed by addition of ethyl 4-amino-3,3-dimethyl-butanoate (**31**) (0.68 g, 4.28 mmol) and TEA (0.94 mL, 12.8 mmol). The mixture was stirred at rt for 16 h. The mixture was concentrated in vacuo and the residue was diluted with H_2O (10 mL) and extracted with EtOAc (10 mL × 3). The combined organic layers were dried over Na_2SO_4, filtered and concentrated in vacuo. The residue was purified by silica gel column chromatography (eluent: 20% EtOAc in petroleum ether) to afford 0.602 g (40% yield) of ethyl 4-[(5-chloro-2-methyl -benzofuran-7-carbonyl) amino]-3,3-dimethylbutanoate (**S18**) as a colorless oil. ^{1}H NMR (400MHz, $CDCl_3$) δ 7.98 (s, 1H), 7.84 (t, J=6.4 Hz, 1H), 7.56 (s, 1H), 6.44 (s, 1H), 4.17 (q, J=7.2 Hz, 2H), 3.51 (d, J=6.4 Hz, 2H), 2.56 (s, 3H), 2.31 (s, 2H), 1.28 (t, J=7.2 Hz, 3H), 1.18 (s, 6H). LCMS (ESI): m/z = 352.1 $[M+H]^+$.

Step 2: Preparation of 4-(5-chloro-2-methylbenzofuran-7-carboxamido)-3,3-dimethylbutanoic acid (33).

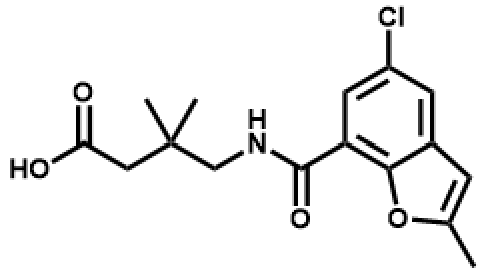

To a mixture of ethyl 4-[(5-chloro-2-methyl-benzofuran-7-carbonyl)amino]-3,3-dimethyl-butanoate (**S18**) (600 mg, 1.71 mmol) in THF (6 mL) and H_2O (2 mL) was added $LiOH{\cdot}H_2O$ (359 mg, 8.55 mmol). The mixture was stirred at rt for 8 h. The mixture was adjusted to pH = 3 with 2 M HCl and extracted with EtOAc (10 mL × 3). The combined organic layers were dried over Na_2SO_4, filtered and concentrated in vacuo to afford 0.271 g (49% yield) of 4-(5-chloro-2-methylbenzofuran-7-carboxamido)-3,3-dimethylbutanoic acid (**33**) as a brown gum. LCMS (ESI): m/z = 324.2 $[M+H]^+$.

Preparation of sodium 4-(5-chloro-2-methylbenzofuran-7-carboxamido)-3-hydroxy-3-methylbutanoate (S19).

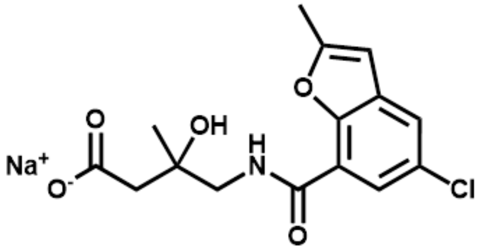

Step 1: Preparation of methyl 4-amino-3-hydroxy-3-methylbutanoate hydrogen chloride (S20).

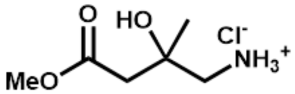

To a mixture of 4-hydroxy-4-methyl-2-pyrrolidinone (250 mg, 2.17 mmol) in MeOH (10.9 mL) was added concd HCl (15.9 µL) at rt. The mixture was heated to 70 °C and stirred for 16 h. The mixture was cooled to rt and concentrated in vacuo to afford 398 mg (99% yield) of methyl 4-amino-3-hydroxy-3-methylbutanoate hydrogen chloride (**S20**) as a colorless oil. This material was used directly in the following step without any further purification. LCMS (ESI): m/z = 148.20 $[M+H]^+$.

Step 2: Preparation of methyl 4-(5-chloro-2-methylbenzofuran-7-carboxamido)-3-hydroxy-3-methylbutanoate (S21).

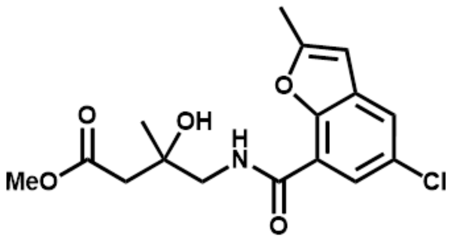

To a mixture of 5-chloro-2-methyl-1-benzofuran-7-carboxylic acid (**25**) (0.010 g, 0.475 mmol) in CH_2Cl_2 (2.4 mL) was added HATU (0.199 g, 0.522 mmol), methyl 4-amino-3-hydroxy-3-methylbutanoate (**S20**) (0.087 g, 0.475 mmol), and DIPEA (0.415 mL, 2.37 mmol). The mixture was stirred at rt for 16 h. The mixture was concentrated in vacuo and the residue purified by silica gel column chromatography (eluent: 0-10% MeOH in

CH_2Cl_2) to afford 0.020 g (12% yield) of methyl 4-(5-chloro-2-methylbenzofuran-7-carboxamido)-3-hydroxy-3-methylbutanoate (**S21**) as a colorless oil. 1H NMR (400 MHz, $CDCl_3$) δ 7.97 (d, J = 2.2 Hz, 1H), 7.91 (s, 1H), 7.58 (d, J = 2.2 Hz, 1H), 6.45 (q, J = 1.2 Hz, 1H), 3.75 – 3.68 (m, 4H), 3.63 (dd, J = 13.8, 5.6 Hz, 1H), 2.72 – 2.56 (m, 2H), 2.54 (d, J = 1.1 Hz, 3H), 1.37 (s, 3H) OH not observed. LCMS (ESI): m/z = 340.10 $[M+H]^+$.

Step 3: Preparation of sodium 4-(5-chloro-2-methylbenzofuran-7-carboxamido)-3-hydroxy-3-methylbutanoate (S19).

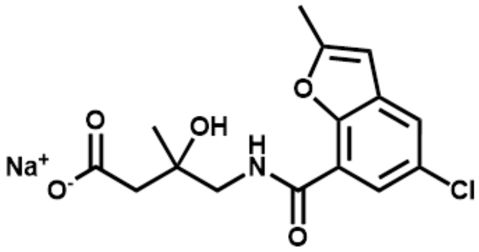

To a mixture of methyl 4-(5-chloro-2-methyl-1-benzofuran-7-ylcarbonylamino)-3-hydroxy-3-methylbutyrate (**S21**) (20.0 mg, 0.059 mmol) in MeOH (0.25 mL) was added 5M NaOH in H_2O (17.0 µL, 0.088 mmol). The mixture was heated to 60 °C and stirred for 1 h. The mixture was concentrated in vacuo to afford sodium 4-(5-chloro-2-methylbenzofuran-7-carboxamido)-3-hydroxy-3-methylbutanoate (**S19**) as a white to clear tacky solid. This material was used directly in the synthesis of **5** without further purification. LCMS (ESI): m/z = 326.50 $[M-Na+2H]^+$.

Preparation of 4-[5-(5-Chloro-2-methyl-1-benzofuran-7-yl)-1, 3,4-oxadiazol-2-yl]-3,3-dimethylbutyric acid (S22).

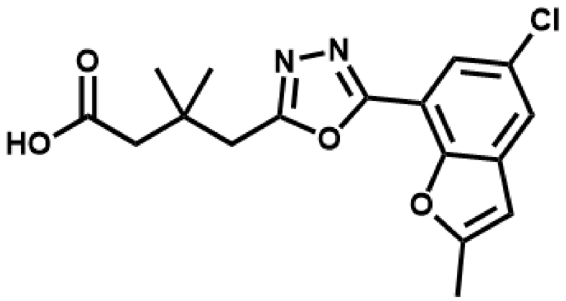

To a mixture of 5-chloro-2-methylbenzofuran-7-carbohydrazide hydrogen chloride (**S40**) (25.0 mg, 0.111 mmol) in phosphoryl trichloride (156 µL, 1.67 mmol), was added 4-methoxycarbonyl-3,3- dimethylbutyric acid (19.4 mg, 0.111 mmol). The reaction mixture was heated to 80 °C and stirred for 1 h. After which the reaction was cooled to rt and the $POCl_3$ was removed in vacuo. The resulting residue was dissolved in CH_2Cl_2 (10 mL) and solvent removed in vacuo to afford 4-[5-(5-Chloro-2-methyl-1-benzofuran-7-yl)-1, 3,4-oxadiazol-2-yl]-3,3-dimethylbutyric acid (**S22**) as a clear tacky oil. This material was used directly in the synthesis of **12** without further purification. LCMS (ESI): m/z = 349.10 $[M+H]^+$.

Preparation of lithium 5-((5-chloro-2-methylbenzofuran-7-yl)amino)-3,3-dimethyl-5-oxopentanoate (S23).

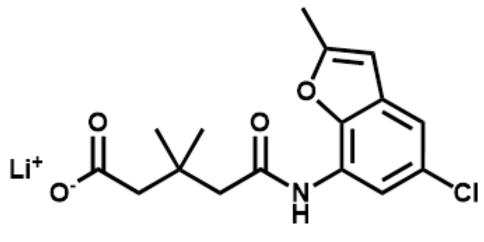

Step 1: Preparation of methyl 5-((5-chloro-2-methylbenzofuran-7-yl)amino)-3,3-dimethyl-5-oxopentanoate (S24).

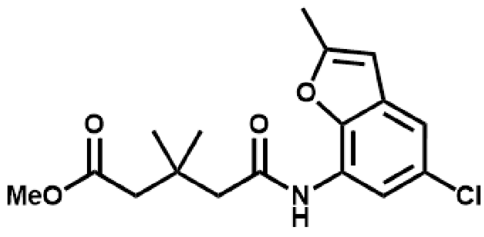

To a mixture of 4-methoxycarbonyl-3,3-dimethylbutyric acid (24.0 mg, 0.138 mmol) in DMF (0.8 mL) was added HATU (57.6 mg, 0.151 mmol) and DIPEA (0.120 mL, 0.688 mmol), the resulting solution was allowed to stir at rt for 15 m. After which 5-chloro-2-methyl-1-benzofuran-7-ylamine (**S42**) (25.0 mg, 0.138 mmol) was added and the reaction mixture was stirred at rt for 16 h. The mixture was concentrated in vacuo, and residue purified by silica gel column chromatography (eluent: 0-10% MeOH in CH_2Cl_2) to afford 30.0 mg (65% yield)

of methyl 5-((5-chloro-2-methylbenzofuran-7-yl)amino)-3,3-dimethyl-5-oxopentanoate (**S24**) as a colorless oil. ^{1}H NMR (400 MHz, CDCl3) δ 8.83 (s, 1H), 8.18 (d, J = 2.0 Hz, 1H), 7.18 (d, J = 2.0 Hz, 1H), 6.34 (t, J = 1.1 Hz, 1H), 3.79 (s, 3H), 2.57 (s, 2H), 2.49 (s, 2H), 2.47 (d, J = 1.1 Hz, 3H), 1.19 (s, 6H). LCMS (ESI): m/z = 338.15 $[M+H]^+$.

Step 2: Preparation of lithium 5-((5-chloro-2-methylbenzofuran-7-yl)amino)-3,3-dimethyl-5-oxopentanoate (S23).

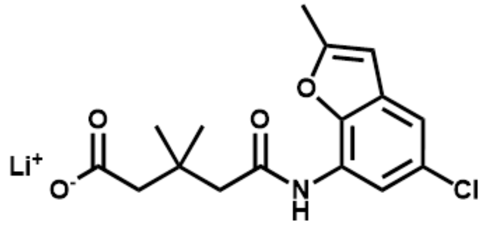

To a mixture of methyl 4-(N-5-chloro-2-methyl-1-benzofuran-7-ylcarbamoyl)-3,3-dimethylbutyrate (**S24**) (30.0 mg, 0.089 mmol) in THF (0.25 mL) was added LiOH•H_2O (7.50 mg, 0.178 mmol) at rt. The mixture was heated to 60 °C and stirred for 2 h. The mixture was concentrated in vacuo and further dried under high vacuum for 30 min to afford lithium 5-((5-chloro-2-methylbenzofuran-7-yl)amino)-3,3-dimethyl-5-oxopentanoate (**S23**) as a tacky white solid. This material was used directly in the synthesis of **13** without further purification. LCMS (ESI): m/z = 324.56 $[M-Li+2H]^+$.

Prearation of 6-amino-N-(1,5-dimethyl-3-oxo-2-phenyl-2,3-dihydro-1H-pyrazol-4-yl)bicyclo[4.1.1]octane-1-carboxamide hydrochloride (**35**).

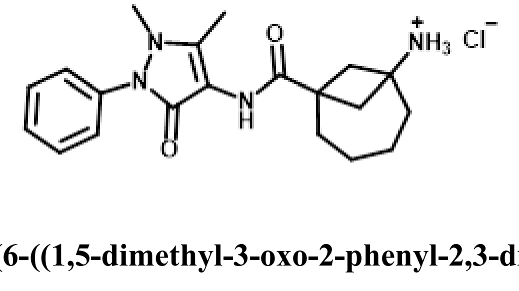

Step 1: Preparation of tert-butyl (6-((1,5-dimethyl-3-oxo-2-phenyl-2,3-dihydro-1H-pyrazol-4-yl)carbamoyl)bicyclo[4.1.1]octan-1-yl)carbamate (S25).

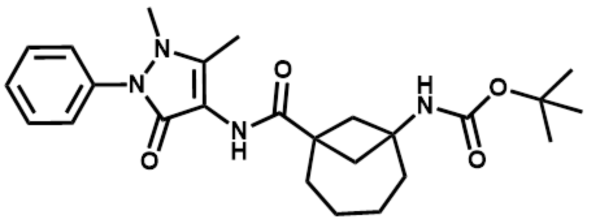

To a mixture of 6-((tert-butoxycarbonyl)amino)bicyclo[4.1.1]octane-1-carboxylic acid (**34**) (100 mg, 0.371 mmol) in CH_2Cl_2 (2.0 mL) was added DIPEA (0.194 mL, 1.11 mmol) and HATU (156 mg, 0.411 mmol) at rt. The reaction mixture was stirred at rt for 15 min before the addition of 24-amino-1,5-dimethyl-2-phenyl-1,2-dihydro-3H-pyrazol-3-one (76 mg, 0.374 mmol). The reaction was stirred at rt for 16 h. The mixture was concentrated in vacuo and the residue purified by silica gel column chromatography (eluent: 0-10% MeOH in CH_2Cl_2) to afford 169 mg (99% yield) of tert-butyl (6-((1,5-dimethyl-3-oxo-2-phenyl-2,3-dihydro-1H-pyrazol-4-yl)carbamoyl)bicyclo[4.1.1]octan-1-yl)carbamate (**S25**) as a yellow solid. ^{1}H NMR (400 MHz, $CDCl_3$) δ 7.71 (s, 1H), 7.48 (t, J = 7.7 Hz, 2H), 7.40 (d, J = 7.8 Hz, 2H), 7.33 (t, J = 7.3 Hz, 1H), 4.81 (s, 1H), 3.11 (s, 3H), 2.41 – 2.31 (m, 2H), 2.27 (s, 3H), 1.80 (td, J = 14.4, 6.0 Hz, 10H), 1.44 (s, 9H). LC/MS (ESI): m/z = 455.75 $[M+H]^+$.

Step 2: Prearation of 6-amino-N-(1,5-dimethyl-3-oxo-2-phenyl-2,3-dihydro-1H-pyrazol-4-yl)bicyclo[4.1.1]octane-1-carboxamide hydrochloride (**35**).

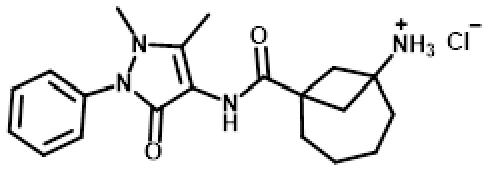

To a mixture of tert-butyl (6-((1,5-dimethyl-3-oxo-2-phenyl-2,3-dihydro-1H-pyrazol-4-yl)carbamoyl)bicyclo[4.1.1]octan-1-yl)carbamate (169 mg, 0.371 mmol) in CH_2Cl_2 (2.0 mL) was added 4 M HCl in 1,4-dioxane (0.371 mL, 1.48 mmol) over 5 min at rt. The mixture was stirred at rt for 16 h. Upon completion, the reaction mixture was concentrated in vacuo to afford 145 mg (99% yield) 6-amino-N-(1,5-dimethyl-3-oxo-2-phenyl-2,3-dihydro-1H-pyrazol-4-yl)bicyclo[4.1.1]octane-1-carboxamide hydrochloride **(35)**

as an orange solid. This material was used directly in the synthesis of **17** and **18** without further purification. LC/MS (ESI): m/z =355.25 $[M+H]^+$.

Preparation of 5-amino-N-(3-methyl-1-oxo-5,6-dihydro-1H-benzo[b]pyrazolo[1,2-d][1,4,5]oxadiazepin-2-yl)bicyclo[3.1.1]heptane-1-carboxamide hydrochloride (S26).

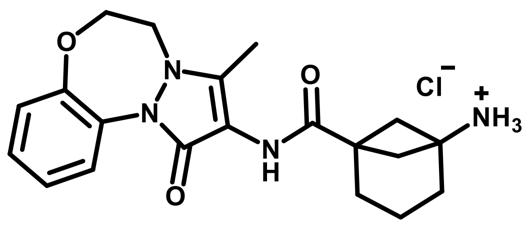

The title compound (**S26**) was prepared using the same procedure as **35**, replacing both carboxylic acid, 6-((tert-butoxycarbonyl)amino)bicyclo[4.1.1]octane-1-carboxylic acid (**34**) and amine, 24-amino-1,5-dimethyl-2-phenyl-1,2-dihydro-3H-pyrazol-3-one with 5-((tert-butoxycarbonyl)amino)bicyclo[3.1.1]heptane-1-carboxylic acid and 2-amino-3-methyl-5,6-dihydro-1H-benzo[b]pyrazolo[1,2-d][1,4,5] oxadiazepin-1-one (**25**) respectively in step 1 to afford 142 mg (94% yield, over two steps) of 5-amino-N-(3-methyl-1-oxo-5,6-dihydro-1H-benzo[b]pyrazolo[1,2-d][1,4,5]oxadiazepin-2-yl)bicyclo[3.1.1]heptane-1-carboxamide hydrochloride (**S26**) as an orange solid. This material was used directly in the synthesis of **15** without further purification.

Preparation of 4-amino-3,3-dimethyl-N-(3-methyl-1-oxo-5,6-dihydro-1H-benzo[b]pyrazolo[1,2-d][1,4,5]oxadiazepin-2-yl)butanamide hydrochloride (S27).

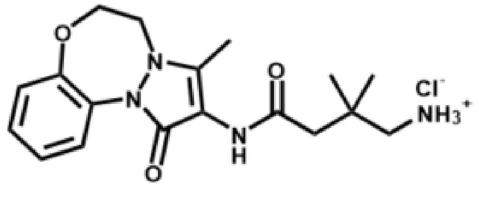

The title compound (**S27**) was prepared using the same procedure as **35**, replacing both carboxylic acid, 6-((tert-butoxycarbonyl)amino)bicyclo[4.1.1]octane-1-carboxylic acid (**34**) and amine, 24-amino-1,5-dimethyl-2-phenyl-1,2-dihydro-3H-pyrazol-3-one with 4-((tert-butoxycarbonyl)amino)-3,3-dimethylbutanoic acid and 2-

amino-3-methyl-5,6-dihydro-1H-benzo[b]pyrazolo[1,2-d][1,4,5] oxadiazepin-1-one (**25**) respectively in step 1 to afford 390 mg (54% yield, over two steps) of 4-amino-3,3-dimethyl-N-(3-methyl-1-oxo-5,6-dihydro-1H-benzo[b]pyrazolo[1,2-d][1,4,5]oxadiazepin-2-yl)butanamide hydrochloride (**S27**) This material was used directly in the synthesis of **4, 9, 10, 11**. ^{1}H NMR (400 MHz, DMSO-d_6) δ 9.14 (s, 1H), 7.65-7.63 (m, 1H), 7.32-7.28 (m, 1H), 7.22-7.18 (m, 2H), 4.65 (s, 2H), 4.27 (t, J=5.2 Hz, 2H), 3.98 (t, J=5.2 Hz, 2H), 2.39 (s, 2H), 2.11 (s, 3H), 1.13 (s, 6H) NH_3 protons not observed. LCMS (ESI): m/z = 345.2 $[M+H]^+$.

Preparation of 4-amino-N-(3-methyl-1-oxo-5,6-dihydro-1H-benzo[b]pyrazolo[1,2-d][1,4,5]oxadiazepin-2-yl)bicyclo[2.1.1]hexane-1-carboxamide hydrochloride (S28).

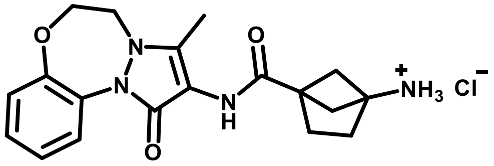

The title compound (**S28**) was prepared using the same procedure as **35**, replacing both carboxylic acid, 6-((tert-butoxycarbonyl)amino)bicyclo[4.1.1]octane-1-carboxylic acid (**34**) and amine, 24-amino-1,5-dimethyl-2-phenyl-1,2-dihydro-3H-pyrazol-3-one with 4-((tert-butoxycarbonyl)amino)bicyclo[2.1.1]hexane-1-carboxylic acid and 2-amino-3-methyl-5,6-dihydro-1H-benzo[b]pyrazolo[1,2-d][1,4,5] oxadiazepin-1-one (**25**) respectively in step 1 to afford 29.6 mg (40% yield, over two steps) of 4-amino-N-(3-methyl-1-oxo-5,6-dihydro-1H-benzo[b]pyrazolo[1,2-d][1,4,5]oxadiazepin-2-yl)bicyclo[2.1.1]hexane-1-carboxamide hydrochloride (**S28**) as an orange solid. This material was used directly in the synthesis of **14** without further purification. LCMS (ESI): m/z = 355.20 $[M+H]^+$.

Preparation of 6-amino-N-(3-methyl-1-oxo-5,6-dihydro-1H-benzo[b]pyrazolo[1,2-d][1,4,5]oxadiazepin-2-yl)bicyclo[4.1.1]octane-1-carboxamide hydrochloride (S29).

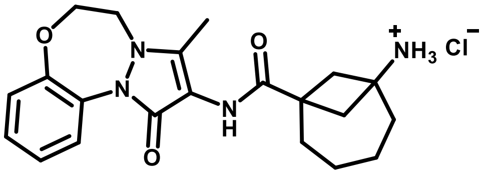

The title compound (**S29**) was prepared using the same procedure as **35**, replacing amine, 24-amino-1,5-dimethyl-2-phenyl-1,2-dihydro-3H-pyrazol-3-one with 2-amino-3-methyl-5,6-dihydro-1H-benzo[b]pyrazolo[1,2-d][1,4,5] oxadiazepin-1-one (**25**) in step 1 to afford 335.5 mg (94% yield, over two steps) of 6-amino-N-(3-methyl-1-oxo-5,6-dihydro-1H-benzo[b]pyrazolo[1,2-d][1,4,5]oxadiazepin-2-yl)bicyclo[4.1.1]octane-1-carboxamide hydrochloride (**S29**) as an orange solid. This material was used directly in the synthesis of **16** without further purification. LCMS (ESI): m/z = 383.20 $[M+H]^+$.

Preparation of 5-chloro-2-ethoxy-4-fluorobenzoic acid (27).

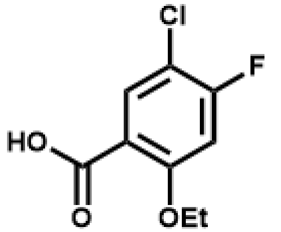

Step 1: Preparation of 5-chloro-4-fluoro-2-hydroxybenzoic acid (S30).

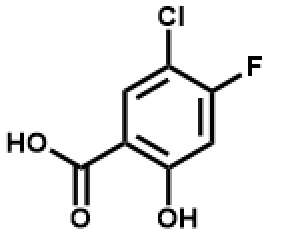

To a mixture of 4-fluoro-2-hydroxybenzoic acid (**26**) (50.0 g, 320 mmol) in CH_2Cl_2 (500 mL) was added a solution of sulfuryl chloride (129 mL, 1601 mmol) in CH_2Cl_2 (300 mL) dropwise at rt, the mixture was stirred at rt for 24 h before the reaction was quenched by addition of H_2O (400 mL). The mixture was extracted with CH_2Cl_2 (400 mL × 3). The combined organic layers were dried over Na_2SO_4, filtered and concentrated in vacuo to afford 50.1 g (82% yield) of 5-chloro-4-fluoro-2-hydroxy-benzoic acid (**S30**) as an off white solid. LCMS (ESI): m/z = 189.0 $[M+H]^+$.

Step 2: Preparation of ethyl 5-chloro-2-ethoxy-4-fluorobenzoate (S31).

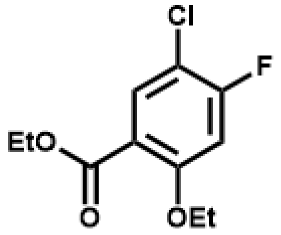

To a mixture of 5-chloro-4-fluoro-2-hydroxybenzoic acid (**S30**) (50.1 g, 263 mmol) and K_2CO_3 (109 g, 789 mmol) in DMF (500 mL) was added iodoethane (63.4 mL, 789 mmol) at rt. The mixture was heated to 70 °C and stirred for 12 h. The mixture was then cooled to rt and filtered. The filtrate was diluted with EtOAc (800 mL) and washed with H_2O (400 mL × 3). The organic layer was dried over Na_2SO_4, filtered and concentrated in vacuo. The residue was purified by silica gel column chromatography (eluent: 5-20% EtOAc in petroleum ether) to afford 36.9 g (57% yield) of ethyl 5-chloro-2-ethoxy-4-fluorobenzoate (**S31**) as a white solid.

Step 3: Preparation of 5-chloro-2-ethoxy-4-fluorobenzoic acid (27).

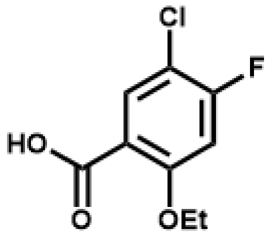

A mixture of ethyl 5-chloro-2-ethoxy-4-fluorobenzoate (**S31**) (36.9 g, 150 mmol) and $LiOH·H_2O$ (18.0 g, 750 mmol) in THF (600 mL) and H_2O (200 mL) was stirred at rt for 12 h. The mixture was concentrated in vacuo to remove THF, the aqueous layer was then adjusted to pH = 3 with 2 M HCl and extracted with EtOAc (300 mL × 3). The combined organic layers were dried over Na_2SO_4, filtered and concentrated in vacuo to afford 29.2 g (89% yield) of 5-chloro-2-ethoxy-4-fluorobenzoic acid (**27**) as a white solid. ^1H NMR (400 MHz, DMSO-d_6): δ 12.90 (s, 1H), 7.79 (d, J = 8.8 Hz, 1H), 7.28 (d, J = 11.6 Hz, 1H) 4.13 (q, J = 7.2 Hz, 2H), 1.33 (t, J = 7.2 Hz, 3H). LCMS (ESI): m/z = 219.2 $[M+H]^+$.

Preparation of 5-chloro-2-methylbenzofuran-7-carboxylic acid (29).

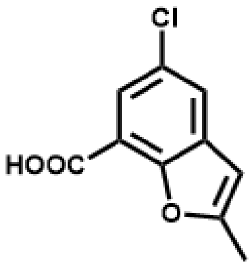

Step 1: Preparation of methyl 5-chloro-2-(prop-2-yn-1-yloxy)benzoate (S32).

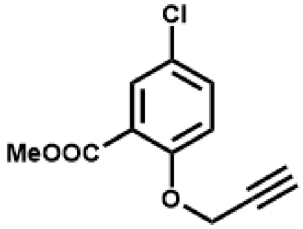

To a mixture of methyl 5-chloro-2-hydroxybenzoate (**28**) (20.0 g, 107 mmol) in MeCN (200 mL) was added 3-bromoprop-1-yne (10.6 mL, 139 mmol) and K_2CO_3 (29.6 g, 214 mmol). The mixture was heated to 80 °C and stirred for 16 h. The mixture was then cooled to rt and concentrated in vacuo. The residue was dissolved in H_2O (100 mL) and extracted with EtOAc (100 mL × 3). The combined organic layers were dried over Na_2SO_4, filtered and concentrated in vacuo. The residue was purified by silica gel column chromatography (eluent: 5-10% EtOAc in petroleum ether) to afford 15.6 g (65% yield) of methyl 5-chloro-2-prop-2-ynoxy-benzoate (**S32**) as a bright yellow solid. ^{1}H NMR (400MHz, $CDCl_3$) δ 7.80 (s, 1H), 7.43 (d, J=8.8 Hz, 1H), 7.09 (d, J=8.8 Hz, 1H), 4.79 (s, 2H), 3.90 (s, 3H), 2.55 (s, 1H).

Step 2: Preparation of methyl 5-chloro-2-methylbenzofuran-7-carboxylate (S33).

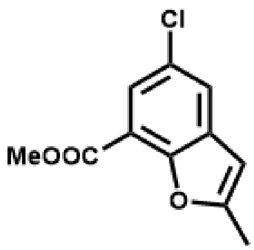

To a mixture of methyl 5-chloro-2 -prop-2-ynoxybenzoate (**S32**) (5.00 g, 22.3 mmol) in N,N-diethylaniline (50 mL) was added CsF (4.10 g, 26.7 mmol). The mixture was heated to 200 °C and stirred for 36 h. The reaction was cooled to rt and diluted with H_2O (100 mL) and the mixture was extracted with EtOAc (50 mL × 3). The combined organic layers were washed with 2 M HCl (100 mL × 5). The organic layer was dried over Na_2SO_4, filtered and concentrated in vacuo. The residue was purified by silica gel column chromatography (eluent: 20% EtOAc in petroleum ether) to afford 2.25 g (45% yield) of methyl-5-chloro-2-methyl-benzofuran-7-carboxylate (**S33**) as a brown solid. ^{1}H NMR (400MHz, $CDCl_3$) δ 7.80 (d, J=6.0 Hz, 1H), 7.60 (s, 1H), 6.39 (s, 1H), 4.00 (s, 3H), 2.53 (s, 3H). LC/MS (ESI): m/z = 225.1 $[M+H]^+$.

Step 3: Preparation of 5-chloro-2-methylbenzofuran-7-carboxylic acid (29).

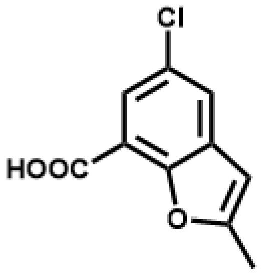

To a mixture of methyl 5-chloro-2-methyl-benzofuran-7-carboxylate (**S33**) (2.25 g, 9.13 mmol) in EtOH (30 mL) and H_2O (10 mL) was added $LiOH{\cdot}H_2O$ (2.30 g, 54.8 mmol). The mixture was stirred at rt for 16 h. The mixture was adjusted to pH = 3 with 2 M HCl and extracted with EtOAc (10 mL × 3). The combined organic layers were dried over Na_2SO_4, filtered and concentrated in vacuo to afford 1.23 g (64% yield) of 5-chloro-2-methyl-benzofuran-7-carboxylic acid (**29**) as a white solid. ^{1}H NMR (400MHz, DMSO-d_6) δ 7.86 (s, 1H), 7.66 (s, 1H), 6.67 (s, 1H), 2.50 (s, 3H) OH not observed. LC/MS (ESI): m/z = 211.0 $[M+H]^+$.

Preparation of 5-chloro-2-(difluoromethyl)benzofuran-7-carboxylic acid (S34).

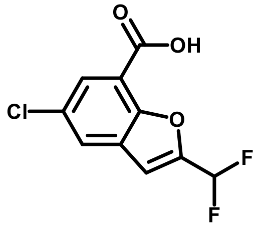

Step 1: Preparation of methyl 5-chloro-2-formylbenzofuran-7-carboxylate (S35).

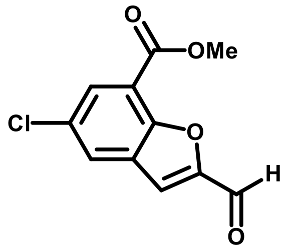

To a solution of methyl 3-allyl-5-chloro-2-hydroxybenzoate (5.50 g, 24.3 mmol) in DMSO (55.0 mL), were added $Cu(OAc)_2$ (0.881 g, 4.85 mmol) and $Pd(OAc)_2$ (0.545 g, 2.43 mmol) at rt. The reaction mixture was purged with an oxygen balloon for 15 m, then pressurized with 50 psi of oxygen, heated to 100 °C and stirred for 18 h in a 250 mL steel bomb flask. The reaction mixture was filtered, the filtrate was poured into ice-cold H_2O (250 mL), and extracted with EtOAc (250 mL × 2). The combined organic layers were dried over Na_2SO_4, filtered and concentrated in vacuo. The residue was purified by silica gel column chromatography (eluent: 10-15% EtOAc in petroleum ether) to afford 0.55 g (9.5% yield) of methyl 5-chloro-2-formylbenzofuran-7-carboxylate (**S35**) as a yellow solid. LCMS (ESI): m/z = 238.8 $[M+H]^+$.

Step 2: Preparation of methyl 5-chloro-2-(difluoromethyl)benzofuran-7-carboxylate (S36).

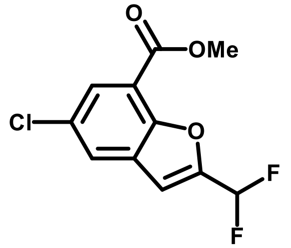

To a stirring solution of methyl 5-chloro-2-formylbenzofuran-7-carboxylate (**S35**) (0.450 g, 1.89 mmol) in DCE (9.0 mL) was added DAST (6.08 g, 37.7 mmol) at 0 °C. The reaction mixture was then allowed to warm to rt and stirred for 1 h. The reaction was cooled to 0 °C and saturated $NaHCO_3$ solution (50 mL) was slowly added, the mixture was then extracted with EtOAc (25 mL × 2). The combined organic layers were dried over Na_2SO_4, filtered and concentrated in vacuo. The residue was purified by silica gel column chromatography (eluent: 10-

20% EtOAc in petroleum ether) to afford 0.25 g (50% yield) of methyl 5-chloro-2-(difluoromethyl)benzofuran-7-carboxylate (**S36**) as an off-white solid. LCMS (ESI): m/z = 261.0 $[M+H]^+$.

Step 3: Preparation of 5-chloro-2-(difluoromethyl)benzofuran-7-carboxylic acid (S34).

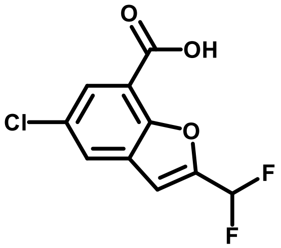

To a solution of methyl 5-chloro-2-(difluoromethyl)benzofuran-7-carboxylate (**S36**) (0.250 g, 0.960 mmol) in a 1:1 mixture of THF and H_2O (7.5 mL) was added $LiOH{\cdot}H_2O$ (0.120 g, 2.88 mmol) at rt. The reaction mixture was stirred at rt for 4 h. The reaction mixture was then concentrated in vacuo and acidified with saturated aqueous $KHSO_4$ solution to pH = 4. A precipitate was formed, the solid was filtered, washed with H_2O (25 mL), and dried under vacuum to afford 0.190 g (80% yield) of 5-chloro-2-(difluoromethyl)benzofuran-7-carboxylic acid **(S34)** as an off-white solid. ^{1}H NMR (400MHz, DMSO-d_6) δ 13.69 (s, 1H), 8.14 (d, J = 2.4 Hz, 1H), 7.91 (d, J = 2 Hz, 1H), 7.48 (d, J = 2 Hz, 1H), 7.35 (t, 1H). LCMS (ESI): m/z = 247.1 $[M+H]^+$.

Preparation of sodium 5-chloro-2-methylbenzo[d]oxazole-7-carboxylate (S37).

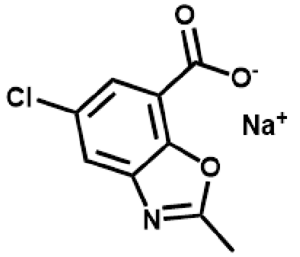

Step 1: Preparation of methyl 5-chloro-2-methylbenzo[d]oxazole-7-carboxylate (S38).

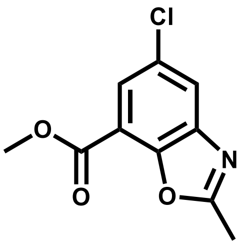

A solution of methyl 3-amino-5-chloro-2-hydroxybenzoate (250 mg, 1.24 mmol) in 1,1,1-triethoxyethane (4.58 mL, 25 mmol) was heated to 120 °C and stirred for 16 h. The mixture was cooled to rt then concentrated in vacuo. The residue was recrystallized from boiling hexanes to afford 145 mg (52% yield) of methyl 5-chloro-2-methylbenzo[d]oxazole-7-carboxylate **(S38)** as a white solid. ^{1}H NMR (400 MHz, $CDCl_3$) δ 7.94 (d, J = 2.1 Hz, 1H), 7.83 (d, J = 2.1 Hz, 1H), 4.03 (s, 3H), 2.74 (s, 3H). LCMS (ESI): m/z = 226.1[M+H]$^+$.

Step 2: Preparation of sodium 5-chloro-2-methylbenzo[d]oxazole-7-carboxylate (S37).

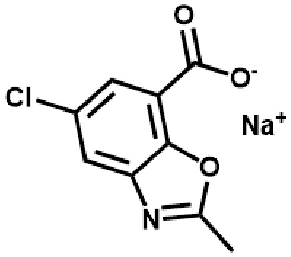

To a mixture of methyl 5-chloro-2-methylbenzo[d]oxazole-7-carboxylate (**S38**) (17.8 mg, 0.079 mmol) in MeOH (0.2 mL) was added a 1 M solution of NaOH in H_2O (0.160 mL, 0.160 mmol) at rt. The mixture was heated to 60 °C and stirred for 1 h. The reaction was cooled to rt and concentrated in vacuo to afford sodium 5-chloro-2-methylbenzo[d]oxazole-7-carboxylate (**S37**) as a white tacky solid. The material was used directly in the synthesis of **9** without any further purification. LCMS (ESI): m/z = 212.50 [M-Na+2H]$^+$.

Preparation of 5-chloro-2-methylfuro[3,2-b]pyridine-7-carboxylic acid (S39).

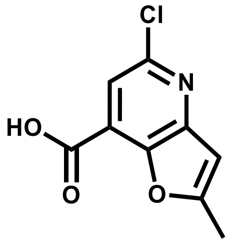

The title compound was prepared using the same procedure as **29**, replacing methyl 5-chloro-2-hydroxybenzoate (**28**) with methyl 2-chloro-5-hydroxyisonicotinate in Step 1. This material was used directly in the synthesis of **4** without any further purification. LCMS (ESI): m/z = 212.05 [M+H]$^+$.

Preparation of 5-chloro-2-methylbenzofuran-7-carbohydrazide hydrochloride (S40).

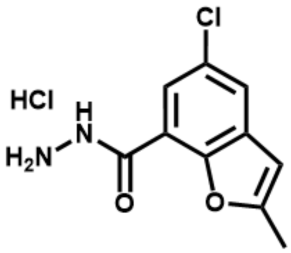

Step 1: Preparation of tert-butyl 2-(5-chloro-2-methylbenzofuran-7-carbonyl) hydrazine-1-carboxylate (S41)

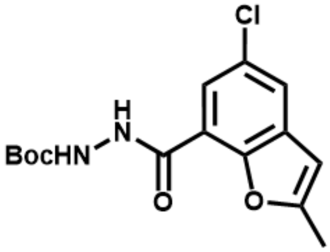

To a solution of 5-chloro-2-methylbenzofuran-7-carboxylic acid **(29)** (0.500 g, 2.37 mmol) in THF (5 mL) was added CDI (0.770 g, 4.74 mmol) at rt and stirred for 2 h. After which tert-Butyl carbazate (0.345 g, 2.61 mmol) was added at rt and allowed to stir for 16 h. The reaction mixture quenched with ice-cold H_2O (25 mL) and extracted with EtOAc (25 mL × 3). The combined organic layers were washed with brine, dried over Na_2SO_4, filtered and concentrated in vacuo. The residue was purified by silica gel column chromatography (eluent: 0-100% EtOAc in petroleum ether) to afford 0.450 g (58% yield) of tert-butyl 2-(5-chloro-2-methylbenzofuran-7-carbonyl) hydrazine-1-carboxylate (**S41**) as an off white solid. LCMS (ESI): m/z = 323.28 $[M-H]^-$.

Step 2: Synthesis of 5-chloro-2-methylbenzofuran-7-carbohydrazide hydrochloride (S40).

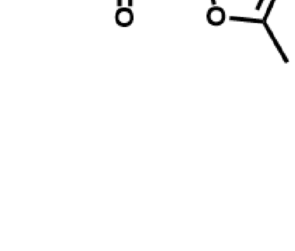

To a stirring solution of tert-butyl 2-(5-chloro-2-methylbenzofuran-7-carbonyl) hydrazine-1-carboxylate (**S41**) (0.450 g, 1.39 mmol) in CH_2Cl_2 (4.5 mL) was added 4 M HCl in 1,4-dioxane (4.50 mL, 4.50 mmol) drop wise at 0 °C, the reaction was warmed to rt and stirred for 2 h. Reaction was concentrated in vacuo and residue purified by trituration with Et_2O to afford 0.330 g (92% yield) of 5-chloro-2-ethoxy-4-fluorobenzohydrazide hydrochloride **(S40)** as an off white solid. ^{1}H NMR (400 MHz, DMSO-d_6): δ 11.09 (s, 1H), 10.55 (br s, 2H), 7.88 (dd, J = 10.8 Hz, 2.0 Hz, 1H), 7.61 (dd, J = 9.6 Hz, 2.4 Hz, 1H), 6.73 (d, J = 1.2 Hz, 1H), 2.50 (s, 3H). LCMS (ESI): m/z = 225.11 $[M+H]^+$.

Preparation of 5-chloro-2-methylbenzofuran-7-amine (S42).

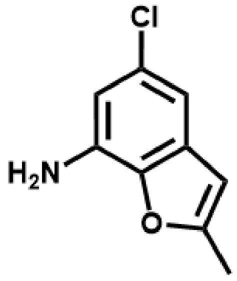

Step 1: Preparation of 4-chloro-2-nitro-1-(prop-2-yn-1-yloxy)benzene (S43).

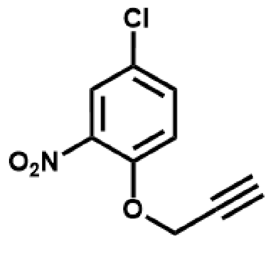

To a stirring solution of 4-chloro-2-nitrophenol (20.0 g, 115 mmol) in MeCN (400 mL) was added K_2CO_3 (31.9 g, 231 mmol) at 0 °C and stirred for 15 m. After which 3-bromoprop-1-yne (11.4 mL, 150 mmol) was added at 0 °C, the reaction was heated to 80 °C and stirred for 4 h. The reaction was cooled to rt and the mixture was poured into ice-cold H_2O (200 mL) and extracted with EtOAc (500 mL × 2). The combined organic layers were washed with brine (200 mL), dried over Na_2SO_4, filtered and concentrated in vacuo. The residue was purified

via trituration with pentane (2 × 50 mL) to afford 20.0 g (82% yield) of 4-chloro-2-nitro-1-(prop-2-yn-1-yloxy)benzene (**S43**) as a brown solid. ^{1}H NMR (400 MHz, $CDCl_3$): δ 7.86 (d, J = 2.4 Hz, 1H), δ 7.52 (dd, J = 9.2 Hz, 1H), 7.23 (t, J = 7.2 Hz, 1H), 4.84 (d, J = 2.4 Hz, 2H), 2.59 (t, J = 7.2 Hz, 1H).

Step 2: Preparation of 5-chloro-2-methyl-7-nitrobenzofuran (S44).

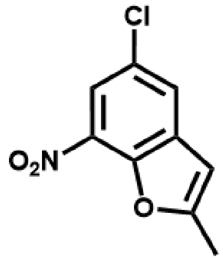

To a solution of 4-chloro-2-nitro-1-(prop-2-yn-1-yloxy)benzene **(S43)** (7.00 g, 33.1 mmol) in *N,N*-Diethylaniline (70 mL) under an N_2 atmosphere was added CsF (6.0 g, 39.7 mmol) at rt. The reaction mass was heated to 200 °C and stirred for 16 h. The reaction was cooled to rt, diluted with H_2O (100 mL) and extracted with EtOAc (2 × 100 mL). The combined organic layers were washed with 1N HCl solution (2 × 100 mL). The organic layer was dried with Na_2SO_4, filtered and concentrated in vacuo. The residue was purified by silica gel column chromatography (eluent: 10% EtOAc in petroleum ether) to afford 1.00 g (14% yield) of 5-chloro-2-methyl-7-nitrobenzofuran **(S44)** as a yellow solid. ^{1}H NMR (400 MHz, $CDCl_3$): δ 8.03 (d, J = 2.0 Hz, 1H), 7.73 (d, J = 2.0 Hz, 1H), 6.49 (d, J = 0.8 Hz, 1H), 2.57 (s, 3H).

Step 3: Preparation of 5-chloro-2-methylbenzofuran-7-amine (S42).

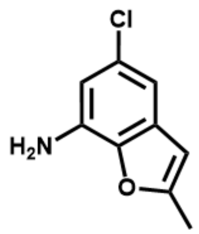

To a solution of 5-chloro-2-methyl-7-nitrobenzofuran (**S44**) (2.0 g, 9.45 mmol) in THF: H_2O (1:1) (50 mL) was added NH_4Cl (2.5 g, 47.3 mmol) and Fe powder (2.6 g, 47.3 mmol) at rt. The reaction mixture was heated to 70 °C and stirred for 4 h. The reaction mixture was cooled to rt and diluted with EtOAc (100 mL), filtered through celite, and filter cake washed with EtOAc (100 mL) and H_2O (100 mL). The organic layer was separated and washed with brine (50 mL), dried over Na_2SO_4, filtered and concentrated in vacuo. The residue was purified by silica gel column chromatography (eluent: 10% EtOAc in petroleum ether), the resulting material was further purified by reverse phase column chromatography (eluent: 30% MeCN in 0.05 M NH_4HCO_3 solution) to afford 0.91 g (53% yield) of 5-chloro-2-methylbenzofuran-7-amine (**S42**) as a white solid.^{1}H NMR (400 MHz, DMSO-d_6): δ 6.69 (d, J = 2.0 Hz, 1H), 6.46 (d, J = 2.0 Hz, 1H), 6.42 (d, J = 0.8 Hz, 1H), 5.51 (s, 2H), 2.4 (s, 3H). LCMS (ESI): m/z = 182.10 $[M+H]^+$.

5-chloro-N-(2,2-dimethyl-4-((3-methyl-1-oxo-5,6-dihydro-1H-benzo[b]pyrazolo[1,2-d][1,4,5]oxadiazepin-2-yl)amino)-4-oxobutyl)-2-ethoxy-4-fluorobenzamide (1).

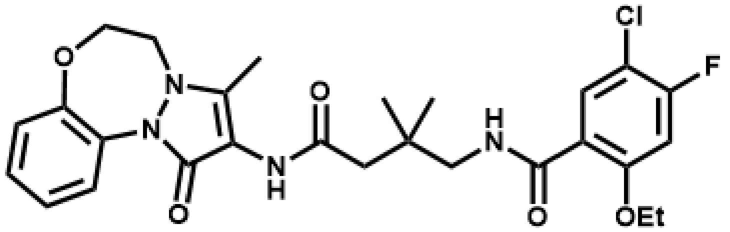

To a mixture of 4-(5-chloro-2-ethoxy-4-fluorobenzamido)-3,3-dimethylbutanoic acid (**33**) (43 mg, 0.13 mmol) and DIPEA (0.066 mL, 0.38 mmol) in DMSO (2 mL) was added HATU (58 mg, 0.15 mmol) at rt, the mixture

was stirred at rt for 15 min after which 2-amino-3-methyl-5,6-dihydro-1H-benzo[b]pyrazolo[1,2-d][1,4,5]oxadiazepin-1-one (**25**) (30 mg, 0.13 mmol) was added. The mixture was allowed to stir at rt for 16 h. The crude reaction mixture was purified by preparatory HPLC (eluent: 0-100% MeCN in H_2O) to afford 13.4 mg (19% yield) 5-chloro-N-(2,2-dimethyl-4-((3-methyl-1-oxo-5,6-dihydro-1H-benzo[b]pyrazolo[1,2-d][1,4,5]oxadiazepin-2-yl)amino)-4-oxobutyl)-2-ethoxy-4-fluorobenzamide (**1**) as a white solid. ^{1}H NMR (400 MHz, $CDCl_3$) δ 8.94 (s, 1H), 8.21 (d, *J* = 8.8 Hz, 1H), 8.15 (t, *J* = 6.6 Hz, 1H), 7.77 – 7.70 (m, 1H), 7.21 – 7.13 (m, 1H), 7.13 – 7.02 (m, 2H), 6.70 (d, *J* = 10.5 Hz, 1H), 4.24 (t, *J* = 4.9 Hz, 2H), 4.09 (q, *J* = 7.0 Hz, 2H), 3.85 (t, *J* = 4.9 Hz, 2H), 3.46 (d, *J* = 6.5 Hz, 2H), 2.22 (s, 2H), 2.17 (s, 3H), 1.45 (t, *J* = 6.9 Hz, 3H), 1.02 (s, 6H). LCMS (ESI): m/z = 545.10 $[M+H]^+$.

5-chloro-N-(2,2-dimethyl-4-((3-methyl-1-oxo-5,6-dihydro-1H-benzo[b]pyrazolo[1,2-d][1,4,5]oxadiazepin-2-yl)amino)-4-oxobutyl)-2-methylbenzofuran-7-carboxamide (**2**).

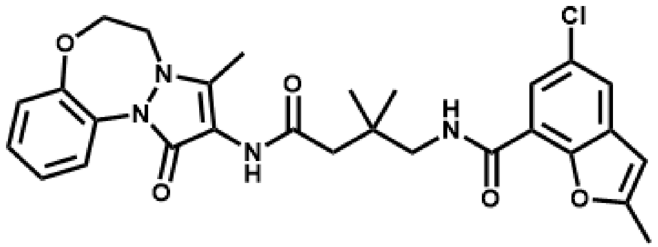

The title compound (**2**) was prepared using the same procedure as **1,** replacing 4-(5-chloro-2-ethoxy-4-fluorobenzamido)-3,3-dimethylbutanoic acid (**32**) with 4-(5-chloro-2-methylbenzofuran-7-carboxamido)-3,3-dimethylbutanoic acid (**33**). The crude reaction mixture was purified by preparatory HPLC (eluent: 0-100% MeCN in H_2O) to afford 11.1 mg (16% yield) of 5-chloro-N-(2,2-dimethyl-4-((3-methyl-1-oxo-5,6-dihydro-1H-benzo[b]pyrazolo[1,2-d][1,4,5]oxadiazepin-2-yl)amino)-4-oxobutyl)-2-methylbenzofuran-7-carboxamide **(2)** as a yellow solid. ^{1}H NMR (400 MHz, DMSO-d_6) δ 9.17 (s, 1H), 8.44 (t, J = 6.3 Hz, 1H), 7.77 (d, J = 2.1 Hz, 1H), 7.63 (d, J = 8.0 Hz, 1H), 7.57 (d, J = 2.2 Hz, 1H), 7.30 (t, J = 7.7 Hz, 1H), 7.21 (t, J = 7.6 Hz, 2H), 6.69 (s, 1H), 4.28 (t, J = 5.0 Hz, 2H), 3.98 (t, J = 5.1 Hz, 2H), 3.38 (d, J = 6.3 Hz, 2H), 2.48 (s, 3H), 2.31 (s, 2H), 2.11 (s, 3H), 1.08 (s, 6H). LCMS (ESI): m/z = 537.2 $[M+H]^+$.

5-chloro-N-(2,2-dimethyl-4-((9-methyl-11-oxo-6,7-dihydro-11H-pyrazolo[1,2-d]pyrido[4,3-b][1,4,5]oxadiazepin-10-yl)amino)-4-oxobutyl)-2-methylbenzofuran-7-carboxamide (3).

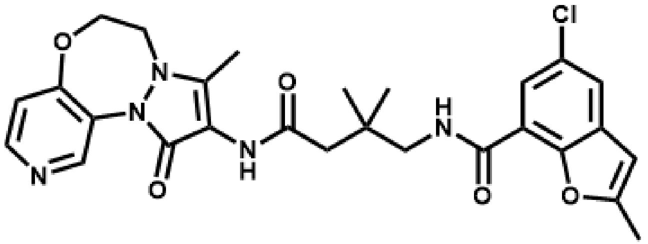

The title compound (**3**) was prepared using the same procedure as **2,** replacing 2-amino-3-methyl-5,6-dihydro-1H-benzo[b]pyrazolo[1,2-d][1,4,5]oxadiazepin-1-one (**25**) with 10-amino-9-methyl-6H-pyrazolo[1,2-d]pyrido[4,3-b][1,4,5]oxadiazepin-11(7H)-one (**S4**). The crude reaction mixture was purified by preparatory HPLC (eluent: 0-100% MeCN in H_2O) to afford 28.1 mg (24% yield) of 5-chloro-N-(2,2-dimethyl-4-((9-methyl-11-oxo-7,11-dihydro-6H-pyrazolo[1,2-d]pyrido[4,3-b][1,4,5]oxadiazepin-10-yl)amino)-4-oxobutyl)-2-methylbenzofuran-7-carboxamide **(3)** as a white solid. ^{1}H NMR (400MHz, DMSO-d_6) δ 9.23 (s, 1H), 8.97 (s, 1H), 8.58 (d, J=6.4 Hz, 1H), 8.40 (t, J=6.1 Hz, 1H), 7.76 (d, J=2.2 Hz, 1H), 7.56 (d, J=2.0 Hz, 1H), 7.51 (d, J=6.0 Hz, 1H), 6.69 (s, 1H), 4.63-4.57 (m, 2H), 4.35-4.31 (m, 2H), 3.36 (d, J=6.0 Hz, 2H), 2.48 (s, 3H), 2.32 (s, 2H), 2.13 (s, 3H), 1.07(s, 6H). LCMS (EIS): m/z = 538.3 $[M+H]^+$.

5-chloro-N-(2,2-dimethyl-4-((3-methyl-1-oxo-5,6-dihydro-1H-benzo[b]pyrazolo[1,2-d][1,4,5]oxadiazepin-2-yl)amino)-4-oxobutyl)-2-methylfuro[3,2-b]pyridine-7-carboxamide (4).

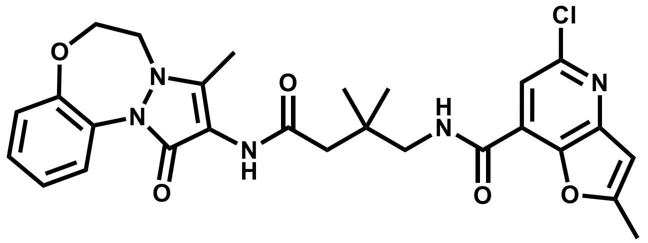

To a mixture of 5-chloro-2-methylfuro[3,2-b]pyridine-7-carboxylic acid (**S39**) (17.8 mg, 0.084 mmol) and DIPEA (0.043 mL, 0.25 mmol) in DMSO (0.6 mL) was added HATU (34.2 mg, 0.090 mmol) at rt, the mixture

was allowed to stir at rt for 15 m, after which 4-amino-3,3-dimethyl-N-(3-methyl-1-oxo-5,6-dihydro-1H-benzo[b] pyrazolo[1,2-d][1,4,5]oxadiazepin-2-yl)butanamide hydrochloride (**S27**) (32.0 mg, 0.084 mmol) was added and the mixture was allowed to stir at rt for 16 h. The crude reaction mixture was purified by preparatory HPLC (eluent: 0-100% MeCN in H_2O) to afford 16.7 mg (37% yield, over two steps) of 5-chloro-N-(2,2-dimethyl-4-((3-methyl-1-oxo-5,6-dihydro-1H-benzo[b]pyrazolo[1,2-d][1,4,5]oxadiazepin-2-yl)amino)-4-oxobutyl)-2-methylfuro[3,2-b]pyridine-7-carboxamide **(4)** as a brown solid. ^{1}H NMR (400 MHz, MeOD-*d*$_4$) δ 8.78 (t, J = 6.5 Hz, 1H), 8.15 (s, 1H), 7.59 (s, 1H), 7.65 (d, J = 7.8 Hz, 1H), 7.41 – 7.35 (m, 1H), 7.29 – 7.20 (m, 2H), 6.72 (s, 1H), 4.36 (t, J = 5.2 Hz, 2H), 4.20 (t, J = 5.2 Hz, 2H), 3.57 (m, 2H), 2.60 (s, 3H), 2.47 (s, 2H), 2.25 (s, 3H), 1.22 (s, 6H). LCMS (EIS): m/z = 538.2 $[M+H]^+$.

5-chloro-N-(2-hydroxy-2-methyl-4-((3-methyl-1-oxo-5,6-dihydro-1H-benzo[b]pyrazolo[1,2-d][1,4,5]oxadiazepin-2-yl)amino)-4-oxobutyl)-2-methylbenzofuran-7-carboxamide (5).

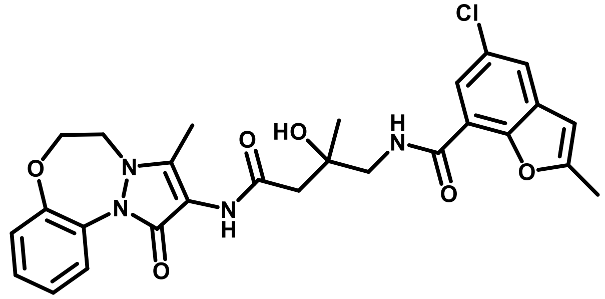

The title compound (**5**) was prepared using the same procedure as **1,** replacing 4-(5-chloro-2-ethoxy-4-fluorobenzamido)-3,3-dimethylbutanoic acid **(32)** with sodium 4-(5-chloro-2-methylbenzofuran-7-carboxamido)-3-hydroxy-3-methylbutanoate (**S19**). The crude reaction mixture was purified by preparatory HPLC (eluent: 0-100% MeCN in H_2O) to afford 10 mg (33% yield, over two steps) of 5-chloro-N-(2-hydroxy-2-methyl-4-((3-methyl-1-oxo-5,6-dihydro-1H-benzo[b]pyrazolo[1,2-d][1,4,5]oxadiazepin-2-yl)amino)-4-oxobutyl)-2-methylbenzofuran-7-carboxamide **(5)** as a brown oil. ^{1}H NMR (400 MHz, CD_3CN) δ 8.26 (s, 1H), 8.06 (s, 1H), 7.97 (t, J = 6.1 Hz, 1H), 7.77 (d, J = 2.3 Hz, 1H), 7.70 – 7.63 (m, 2H), 7.32 (td, J = 7.7, 1.7 Hz, 1H), 7.24 – 7.17 (m, 2H), 6.55 (t, J = 1.2 Hz, 1H), 4.30 (t, J = 5.1 Hz, 2H), 4.00 (t, J = 5.2 Hz, 2H), 3.67 – 3.56

(m, 2H), 2.65 (d, J = 14.4 Hz, 1H), 2.55 (d, J = 14.4 Hz, 1H), 2.50 (d, J = 1.1 Hz, 3H), 2.17 (s, 3H), 1.35 (s, 3H). LCMS (EIS): m/z = 539.2 $[M+H]^+$.

5-chloro-N-(2,2-dimethyl-4-((2-methyl-5,6-dihydrobenzo[d]pyrazolo[5,1-b][1,6,3]dioxazocin-3-yl)amino)-4-oxobutyl)-2-methylbenzofuran-7-carboxamide (6)

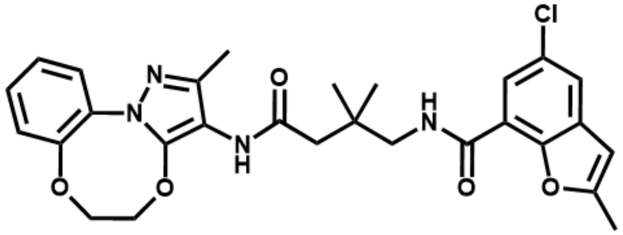

The title compound (**6**) was prepared using the same procedure as **2,** replacing 2-amino-3-methyl-5,6-dihydro-1H-benzo[b]pyrazolo[1,2-d][1,4,5]oxadiazepin-1-one (**25**) with 2-amino-3-methyl-5,6-dihydro-1H-benzo[b]pyrazolo[1,2-d][1,4,5]oxadiazepin-1-one **(S10).** The crude reaction mixture was purified by preparatory HPLC (eluent: 0-100% MeCN in H_2O) to afford 17.5 mg (48% yield) of 5-chloro-N-(2,2-dimethyl-4-((2-methyl-5,6-dihydrobenzo[d]pyrazolo[5,1-b][1,6,3]dioxazocin-3-yl)amino)-4-oxobutyl)-2-methylbenzofuran-7-carboxamide as a white solid. ^1H NMR (400 MHz, DMSO-d_6) δ 9.19 (s, 1H), 8.44 (t, *J* = 6.4 Hz, 1H), 7.78 (d, *J* = 2.2 Hz, 1H), 7.58 (d, *J* = 2.2 Hz, 1H), 7.45 (dd, *J* = 7.9, 1.7 Hz, 1H), 7.42 (dd, *J* = 7.1, 1.6 Hz, 1H), 7.35 – 7.27 (m, 2H), 6.69 (d, *J* = 1.5 Hz, 1H), 4.35 – 4.17 (m, 4H), 3.40 (d, *J* = 6.3 Hz, 2H), 2.46 (s, 3H), 2.32 (s, 2H), 2.08 (s, 3H), 1.11 (s, 6H). LCMS (EIS): m/z = 537.2 $[M+H]^+$.

5-chloro-N-(4-((5,6-dihydrobenzo[f]pyrazolo[1,5-d][1,4]oxazepin-2-yl)amino)-2,2-dimethyl-4-oxobutyl)-2-methylbenzofuran-7-carboxamide (7).

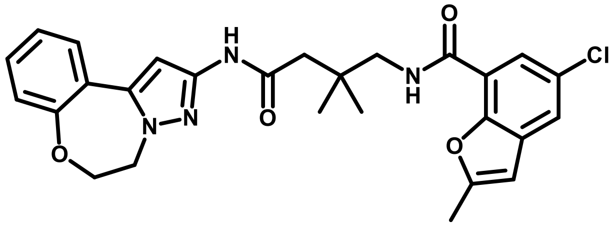

The title compound (**7**) was prepared using the same procedure as **2,** replacing 2-amino-3-methyl-5,6-dihydro-1H-benzo[b]pyrazolo[1,2-d][1,4,5]oxadiazepin-1-one (**25**) with 5,6-dihydrobenzo[f]pyrazolo[1,5-d][1,4]oxazepin-2-amine (**S6**). The crude reaction mixture was purified by preparatory HPLC (eluent: 0-100% MeCN in H_2O) to afford 38 mg (49% yield) of 5-chloro-N-(4-((5,6-dihydrobenzo[f]pyrazolo[1,5-d][1,4]oxazepin-2-yl)amino)-2,2-dimethyl-4-oxobutyl)-2-methylbenzofuran-7-carboxamide (**7**) as an off-white solid. ^{1}H NMR (400 MHz, DMSO-d_6) δ 10.54 (s, 1H), 8.42 (t, J = 6.3 Hz, 1H), 7.75 (dd, J = 8.7, 1.9 Hz, 2H), 7.58 (d, J = 2.2 Hz, 1H), 7.28 (ddd, J = 8.5, 7.1, 1.6 Hz, 1H), 7.10 (ddd, J = 8.3, 7.1, 1.4 Hz, 1H), 7.04 (d, J = 6.9 Hz, 2H), 6.68 (d, J = 1.2 Hz, 1H), 4.53 – 4.48 (m, 2H), 4.44 (dt, J = 6.7, 2.4 Hz, 2H), 3.38 (s, 2H), 2.49 (s, 3H), 2.36 (s, 2H), 1.07 (s, 6H). LCMS (EIS): m/z = 507.2 $[M+H]^+$.

5-chloro-N-(2,2-dimethyl-4-oxo-4-((1-oxo-5,6-dihydro-1H-benzo[b]pyrazolo[1,2-d][1,4,5]oxadiazepin-2-yl)amino)butyl)-2-methylbenzofuran-7-carboxamide (8).

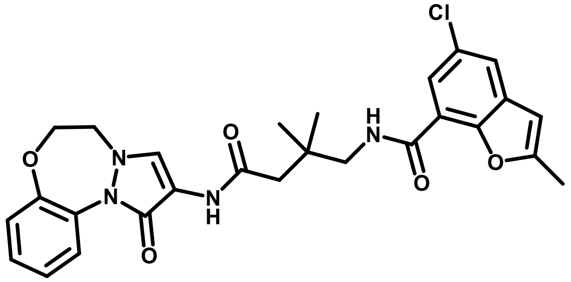

The title compound (**8**) was prepared using the same procedure as **2,** replacing 2-amino-3-methyl-5,6-dihydro-1H-benzo[b]pyrazolo[1,2-d][1,4,5]oxadiazepin-1-one (**25**) with 2-amino-5,6-dihydro-1H-benzo[b]pyrazolo[1,2-d][1,4,5]oxadiazepin-1-one (**S5**). The crude reaction mixture was purified by preparatory HPLC (eluent: 0-100% MeCN in H_2O) to afford 7.0 mg (49% yield) 5-chloro-N-(2,2-dimethyl-4-oxo-4-((1-oxo-5,6-dihydro-1H-benzo[b]pyrazolo[1,2-d][1,4,5]oxadiazepin-2-yl)amino)butyl)-2-methylbenzofuran-7-carboxamide **(8)** as a white solid. ^{1}H NMR (400 MHz, DMSO-d_6) δ 9.84 (s, 1H), 8.36 (t, J = 6.3 Hz, 1H), 8.28 (s, 1H), 7.78 (d, J = 2.2 Hz, 1H), 7.68 (dd, J = 8.4, 1.7 Hz, 1H), 7.56 (d, J = 2.2 Hz, 1H), 7.32 (td, J = 7.6, 1.7 Hz, 1H), 7.20 (t, J = 7.2 Hz, 2H), 6.70 (t, J = 1.0 Hz, 1H), 4.25 (dd, J = 5.5, 3.6 Hz, 2H), 3.90 – 3.82 (m, 2H), 3.36 (d, J = 6.3 Hz, 2H), 2.50 (s, 3H), 2.45 (s, 2H), 1.05 (s, 6H). LCMS (EIS): m/z = 523.2 $[M+H]^+$.

5-chloro-N-(2,2-dimethyl-4-((3-methyl-1-oxo-5,6-dihydro-1H-benzo[b]pyrazolo[1,2-d][1,4,5]oxadiazepin-2-yl)amino)-4-oxobutyl)-2-methylbenzo[d]oxazole-7-carboxamide (9).

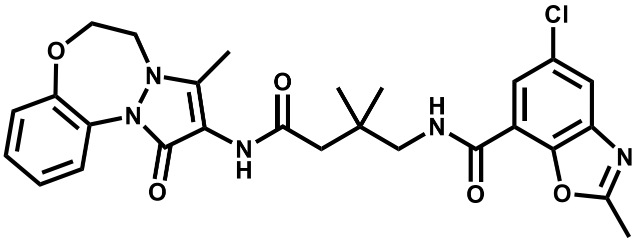

The title compound (**9**) was prepared using the same procedure as **4**, replacing 5-chloro-2-methylfuro[3,2-b]pyridine-7-carboxylic acid (**S39**) with sodium 5-chloro-2-methylbenzo[d]oxazole-7-carboxylate (**S37**). The crude reaction mixture was purified by preparatory HPLC (eluent: 0-100% MeCN in H_2O) to afford 7.4 mg (17% yield, over two steps) of 5-chloro-N-(2,2-dimethyl-4-((3-methyl-1-oxo-5,6-dihydro-1H-benzo[b]pyrazolo[1,2-d][1,4,5]oxadiazepin-2-yl)amino)-4-oxobutyl)-2-methylbenzo[d]oxazole-7-carboxamide **(9)** as a brown solid. ^{1}H NMR (400 MHz, MeOD-d_4) δ 7.81 (m, 2H), 7.66 (dd, J = 8.0, 1.7 Hz, 1H), 7.39 (t, J = 7.7 Hz, 1H), 7.30 – 7.21 (m, 2H), 4.37 (t, J = 5.3 Hz, 2H), 4.20 (t, J = 5.2 Hz, 2H), 3.56 (s, 2H), 2.70 (s, 3H), 2.47 (m, 2H), 2.25 (s, 3H), 1.21 (s, 6H) two NH protons are not located. LRMS: m/z = 538.2 $[M+H]^+$.

5-chloro-N-(2,2-dimethyl-4-((3-methyl-1-oxo-5,6-dihydro-1H-benzo[b]pyrazolo[1,2-d][1,4,5]oxadiazepin-2-yl)amino)-4-oxobutyl)benzofuran-7-carboxamide (10).

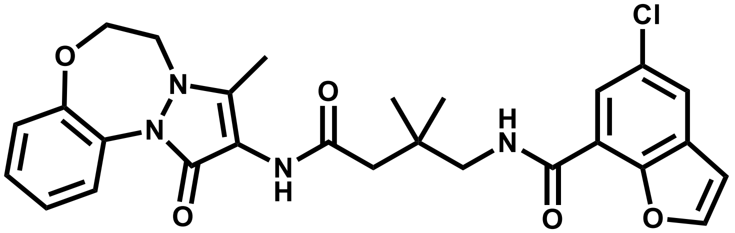

The title compound (**10**) was prepared using the same procedure as **4**, replacing 5-chloro-2-methylfuro[3,2-b]pyridine-7-carboxylic acid (**S39**) with 5-chlorobenzofuran-7-carboxylic acid. The crude reaction mixture was purified by preparatory HPLC (eluent: 0-100% MeCN in H_2O) to afford 16.1 mg (45% yield) of 5-chloro-N-(2,2-dimethyl-4-((3-methyl-1-oxo-5,6-dihydro-1H-benzo[b]pyrazolo[1,2-d][1,4,5]oxadiazepin-2-yl)amino)-4-

oxobutyl)benzofuran-7-carboxamide (**10**) as a yellow solid. ^{1}H NMR (400MHz, MeOD-d_4) δ 8.62 (t, J = 6.6 Hz, 1H), 8.18 (s, 1H), 8.00 (t, J = 1.7 Hz, 1H), 7.84 (m, 2H), 7.72 – 7.65 (m, 1H), 7.39 (t, J = 7.8 Hz, 1H), 7.30 – 7.21 (m, 2H), 6.99 (t, J = 1.7 Hz, 1H), 4.37 (t, J = 5.2 Hz, 2H), 4.20 (t, J = 5.2 Hz, 2H), 3.59 (d, J = 6.3 Hz, 2H), 2.46 (s, 2H), 2.26 (s, 3H), 1.22 (s, 6H). LCMS (EIS): m/z = 523.2 $[M+H]^+$.

5-chloro-2-(difluoromethyl)-N-(2,2-dimethyl-4-((3-methyl-1-oxo-5,6-dihydro-1H-benzo[b]pyrazolo[1,2-d][1,4,5]oxadiazepin-2-yl)amino)-4-oxobutyl)benzofuran-7-carboxamide (11).

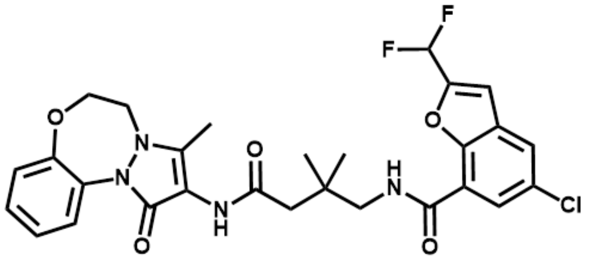

The title compound (**11**) was prepared using the same procedure as **4**, replacing 5-chloro-2-methylfuro[3,2-b]pyridine-7-carboxylic acid (**S39**) with 5-chloro-2-(difluoromethyl)-1-benzofuran-7-carboxylic acid (**S34**). The crude reaction mixture was purified by preparatory HPLC (eluent: 0-100% MeCN in H_2O) to afford 18.7 mg (52% yield) 5-chloro-2-(difluoromethyl)-N-(2,2-dimethyl-4-((3-methyl-1-oxo-5,6-dihydro-1H-benzo[b]pyrazolo[1,2-d][1,4,5]oxadiazepin-2-yl)amino)-4-oxobutyl)benzofuran-7-carboxamide **(11)** as a white solid. ^{1}H NMR (400 MHz, DMSO-d_6) δ 9.13 (s, 1H), 8.52 (t, J = 6.3 Hz, 1H), 8.02 (d, J = 2.2 Hz, 1H), 7.76 (d, J = 2.2 Hz, 1H), 7.63 (dd, J = 8.3, 1.7 Hz, 1H), 7.47 (t, J = 2.1 Hz, 1H), 7.34 (d, J = 12.6 Hz, 1H), 7.29 (dd, J = 7.3, 1.9 Hz, 1H), 7.23 – 7.19 (m, 2H), 4.28 (t, J = 5.0 Hz, 2H), 3.99 (t, J = 5.1 Hz, 2H), 3.37 (d, J = 6.3 Hz, 2H), 2.31 (s, 2H), 2.13 (s, 3H), 1.09 (s, 6H). LCMS (EIS): m/z = 573.2 $[M+H]^+$.

4-(5-(5-chloro-2-methylbenzofuran-7-yl)-1,3,4-oxadiazol-2-yl)-3,3-dimethyl-N-(3-methyl-1-oxo-5,6-dihydro-1H-benzo[b]pyrazolo[1,2-d][1,4,5]oxadiazepin-2-yl)butanamide (12).

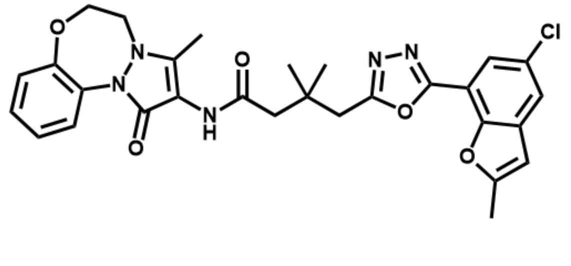

The title compound (**12**) was prepared using the same procedure as **1**, replacing 4-(5-chloro-2-ethoxy-4-fluorobenzamido)-3,3-dimethylbutanoic acid (**32**) with 4-[5-(5-Chloro-2-methyl-1-benzofuran-7-yl)-1, 3,4-oxadiazol-2-yl]-3,3-dimethylbutyric acid (**S22**). The crude reaction mixture was purified by preparatory HPLC (eluent: 0-100% MeCN in H_2O) to afford 6.9 mg (11% yield, over two steps) N-{5-methyl-3-oxo-9-oxa-2,6-diazatricyclo [8.4.0.0^{2,6}]tetradeca-1(14),4,10,12-tetraen-4 -yl}4-[5-(5-chloro-2-methyl-1-benzofuran-7- yl)-1,3,4-oxadiazol-2-yl]-3,3-dimethylbutyramide **(12)** as a white solid. ^{1}H NMR (400 MHz, DMSO-d_6) δ 9.08 (s, 1H), 7.90 (d, J = 2.1 Hz, 1H), 7.78 (d, J = 2.1 Hz, 1H), 7.65 (dd, J = 8.1, 1.8 Hz, 1H), 7.33 – 7.27 (m, 1H), 7.23 – 7.18 (m, 2H), 6.77 (d, J = 1.3 Hz, 1H), 4.28 (t, J = 4.9 Hz, 2H), 3.98 (t, J = 4.9 Hz, 2H), 3.15 (s, 2H), 2.54 (d, J = 1.0 Hz, 3H), 2.41 (s, 2H), 2.14 (s, 3H), 1.19 (s, 6H). LCMS (ESI): m/z = 562.20 [M+H]$^+$.

N^1-(5-chloro-2-methylbenzofuran-7-yl)-3,3-dimethyl-N^5-(3-methyl-1-oxo-5,6-dihydro-1H-benzo[b]pyrazolo[1,2-d][1,4,5]oxadiazepin-2-yl)pentanediamide (13).

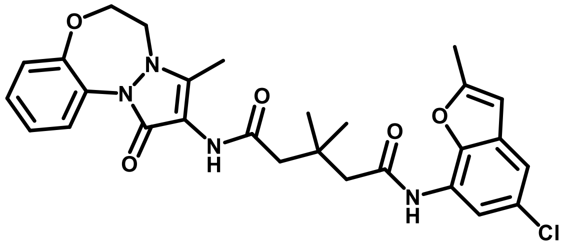

The title compound (**13**) was prepared using the same procedure as **1**, replacing 4-(5-chloro-2-ethoxy-4-fluorobenzamido)-3,3-dimethylbutanoic acid (**32**) with lithium 5-((5-chloro-2-methylbenzofuran-7-yl)amino)-3,3-dimethyl-5-oxopentanoate (**S23**). The crude reaction mixture was purified by preparatory HPLC (0-100% MeCN in H_2O) to afford 2.5 mg (3% yield, over two steps) of N^1-(5-chloro-2-methylbenzofuran-7-yl)-3,3-dimethyl-N^5-(3-methyl-1-oxo-5,6-dihydro-1H-benzo[b]pyrazolo[1,2-d][1,4,5]oxadiazepin-2-yl)pentanediamide (**13**) as a white solid. ^{1}H NMR (400 MHz, DMSO-d_6) δ 10.38 (s, 1H), 9.19 (s, 1H), 7.90 (d, J = 2.1 Hz, 1H), 7.66 (dd, J = 8.2, 1.6 Hz, 1H), 7.35 – 7.27 (m, 2H), 7.21 (t, J = 7.5 Hz, 2H), 6.60 (d, J = 1.2 Hz, 1H), 4.28 (t, J = 4.9 Hz, 2H), 4.00 (t, J = 5.0 Hz, 2H), 2.55 (s, 2H), 2.45 (d, J = 1.1 Hz, 3H), 2.41 (s, 2H), 2.16 (s, 3H), 1.16 (s, 6H). LCMS (EIS): m/z = 537.4 [M+H]$^+$.

5-chloro-2-methyl-N-(4-((3-methyl-1-oxo-5,6-dihydro-1H-benzo[b]pyrazolo[1,2-d][1,4,5]oxadiazepin-2-yl)carbamoyl)bicyclo[2.1.1]hexan-1-yl)benzofuran-7-carboxamide (14).

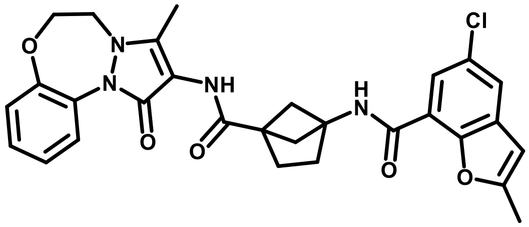

The title compound (**14**) was prepared using the same procedure as **15**, replacing 5-amino-N-(3-methyl-1-oxo-5,6-dihydro-1H-benzo[b]pyrazolo[1,2-d][1,4,5]oxadiazepin-2-yl)bicyclo[3.1.1]heptane-1-carboxamide hydrochloride (**S26**) with 4-amino-N-(3-methyl-1-oxo-5,6-dihydro-1H-benzo[b]pyrazolo[1,2-d][1,4,5]oxadiazepin-2-yl)bicyclo[2.1.1]hexane-1-carboxamide hydrochloride (**S28**). The crude reaction mixture was purified by preparatory HPLC (eluent: 0-100% MeCN in H_2O) to afford 2.5 mg (5% yield) 5-chloro-2-methyl-N-(4-((3-methyl-1-oxo-5,6-dihydro-1H-benzo[b]pyrazolo[1,2-d][1,4,5]oxadiazepin-2-yl)carbamoyl)bicyclo[2.1.1]hexan-1-yl)benzofuran-7-carboxamide (**14**) as a white solid. ^{1}H NMR (400 MHz, DMSO-d_6) δ 8.81 (s, 1H), 8.78 (s, 1H), 7.76 (d, J = 2.2 Hz, 1H), 7.66 (dd, J = 7.8, 1.7 Hz, 1H), 7.51 (d, J = 2.2 Hz, 1H), 7.31 (td, J = 7.7, 1.7 Hz, 1H), 7.22 (td, J = 9.2, 1.7 Hz, 2H), 6.72 – 6.65 (m, 1H), 4.29 (t, J = 5.0 Hz, 2H), 3.99 (t, J = 5.0 Hz, 2H), 2.50 (s, 3H), 2.23 (d, J = 3.5 Hz, 2H), 2.10 (s, 3H), 2.01 (d, J = 7.5 Hz, 2H), 1.95 (dd, J = 9.2, 4.2 Hz, 2H), 1.88 (dd, J = 3.8, 1.9 Hz, 2H). LCMS (EIS): m/z = 547.2 $[M+H]^+$.

5-chloro-2-methyl-N-(5-((3-methyl-1-oxo-5,6-dihydro-1H-benzo[b]pyrazolo[1,2-d][1,4,5]oxadiazepin-2-yl)carbamoyl)bicyclo[3.1.1]heptan-1-yl)benzofuran-7-carboxamide (15).

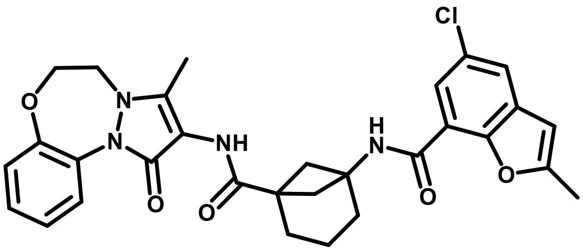

To a mixture of 5-chloro-2-methylbenzofuran-7-carboxylic acid (**29**) (52.0 mg, 0.247 mmol) in DMSO (1.1 mL) was added DIPEA (0.129 mL, 0.741 mmol) and HATU (113.0 mg, 0.296 mmol). The reaction mixture was stirred at rt for 15 min before the addition of 5-amino-N-(3-methyl-1-oxo-5,6-dihydro-1H-benzo[b]pyrazolo[1,2-d][1,4,5]oxadiazepin-2-yl)bicyclo[3.1.1]heptane-1-carboxamide hydrochloride (**S26**) (100 mg, 0.247 mmol). The reaction was stirred at rt for 16 h. The crude reaction mixture was purified by preparatory HPLC (eluent: 0-100% MeCN in H_2O) to afford 47.0 mg (35% yield) 5-chloro-2-methyl-N-(5-((3-methyl-1-oxo-5,6-dihydro-1H-benzo[b]pyrazolo[1,2-d][1,4,5]oxadiazepin-2-yl)carbamoyl)bicyclo[3.1.1]heptan-1-yl)benzofuran-7-carboxamide **(15)** as a white solid. ^{1}H NMR (400 MHz, DMSO-d_6) δ 8.70 (s, 1H), 8.45 (s, 1H), 7.74 (d, J = 2.2 Hz, 1H), 7.65 (dd, J = 7.9, 1.8 Hz, 1H), 7.49 (d, J = 2.2 Hz, 1H), 7.30 (td, J = 7.7, 1.7 Hz, 1H), 7.24 – 7.17 (m, 2H), 6.67 (s, 1H), 4.28 (t, J = 4.9 Hz, 2H), 3.98 (t, J = 4.9 Hz, 2H), 2.50 (s, 3H), 2.35 (d, J = 7.3 Hz, 2H), 2.17 (dt, J = 6.8, 3.7 Hz, 2H), 2.07 (d, J = 4.9 Hz, 5H), 1.93 (d, J = 3.5 Hz, 4H). LCMS (EIS): m/z = 561.3 $[M+H]^+$.

5-chloro-2-methyl-N-(6-((3-methyl-1-oxo-5,6-dihydro-1H-benzo[b]pyrazolo[1,2-d][1,4,5]oxadiazepin-2-yl)carbamoyl)bicyclo[4.1.1]octan-1-yl)benzofuran-7-carboxamide (16).

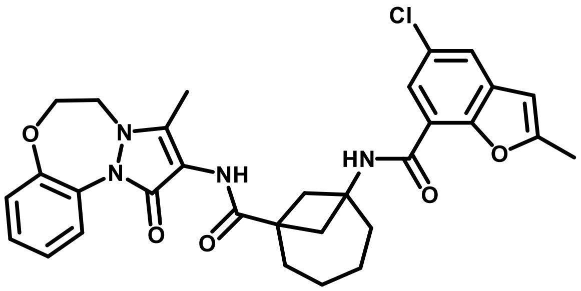

The title compound (**16**) was prepared using the same procedure as **15**, replacing 5-amino-N-(3-methyl-1-oxo-5,6-dihydro-1H-benzo[b]pyrazolo[1,2-d][1,4,5]oxadiazepin-2-yl)bicyclo[3.1.1]heptane-1-carboxamide hydrochloride (**S26**) with 6-amino-N-(3-methyl-1-oxo-5,6-dihydro-1H-benzo[b]pyrazolo[1,2-d][1,4,5]oxadiazepin-2-yl)bicyclo[4.1.1]octane-1-carboxamide hydrochloride (**S29**). The crude reaction mixture was purified by preparatory HPLC (eluent: 0-100% MeCN in H_2O) to afford 14.6 mg (17% yield) of 5-chloro-2-methyl-N-(6-((3-methyl-1-oxo-5,6-dihydro-1H-benzo[b]pyrazolo[1,2-d][1,4,5]oxadiazepin-2-

yl)carbamoyl)bicyclo[4.1.1]octan-1-yl)benzofuran-7-carboxamide **(16)** as a white solid. ^{1}H NMR (400 MHz, DMSO-d_6) δ 8.68 (s, 1H), 8.49 (s, 1H), 7.73 (d, J = 2.2 Hz, 1H), 7.64 (dd, J = 8.4, 1.6 Hz, 1H), 7.48 (d, J = 2.2 Hz, 1H), 7.30 (td, J = 7.4, 1.7 Hz, 1H), 7.20 (td, J = 7.8, 1.4 Hz, 2H), 6.66 (d, J = 1.3 Hz, 1H), 4.27 (t, J = 5.0 Hz, 2H), 3.96 (t, J = 4.9 Hz, 2H), 2.49 – 2.56 (m, 7H), 2.07 (s, 3H), 1.91 (d, J = 5.8 Hz, 2H), 1.78 (m, 6H). LCMS (EIS): m/z = 575.3 $[M+H]^+$.

5-chloro-N-(6-((1,5-dimethyl-3-oxo-2-phenyl-2,3-dihydro-1H-pyrazol-4-yl)carbamoyl)bicyclo[4.1.1]octan-1-yl)-2-methylbenzofuran-7-carboxamide (17).

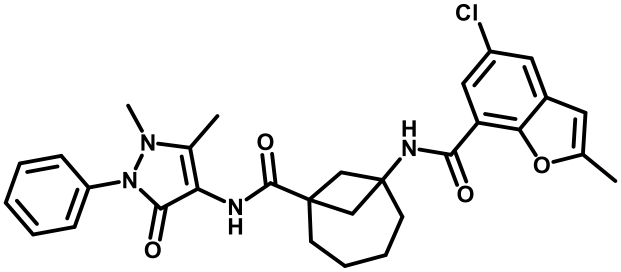

The title compound (**17**) was prepared using the same procedure as **15**, replacing 5-amino-N-(3-methyl-1-oxo-5,6-dihydro-1H-benzo[b]pyrazolo[1,2-d][1,4,5]oxadiazepin-2-yl)bicyclo[3.1.1]heptane-1-carboxamide hydrochloride (**S26**) with 6-amino-N-(1,5-dimethyl-3-oxo-2-phenyl-2,3-dihydro-1H-pyrazol-4-yl)bicyclo[4.1.1]octane-1-carboxamide hydrochloride (**35**). The crude reaction mixture was purified by preparatory HPLC (eluent: 0-100% MeCN in H_2O) to afford 12.9 mg (31% yield) of 5-chloro-N-(6-((1,5-dimethyl-3-oxo-2-phenyl-2,3-dihydro-1H-pyrazol-4-yl)carbamoyl)bicyclo[4.1.1]octan-1-yl)-2-methylbenzofuran-7-carboxamide (**17**) as a white solid. ^{1}H NMR (400 MHz, DMSO-d_6) δ 8.67 (s, 1H), 8.49 (s, 1H), 7.72 (d, J = 2.2 Hz, 1H), 7.53 – 7.44 (m, 3H), 7.37 – 7.28 (m, 3H), 6.65 (d, J = 1.3 Hz, 1H), 3.04 (s, 3H), 2.54 (d, J = 2.2 Hz, 2H), 2.48 (d, J = 8.0 Hz, 5H), 2.09 (s, 3H), 1.91 (t, J = 5.9 Hz, 2H), 1.76 (m, 6H). LCMS (ESI): m/z = 547.3 $[M+H]^+$.

6-chloro-N-(6-((1,5-dimethyl-3-oxo-2-phenyl-2,3-dihydro-1H-pyrazol-4-yl)carbamoyl)bicyclo[4.1.1]octan-1-yl)-2-methylimidazo[1,2-b]pyridazine-8-carboxamide (18).

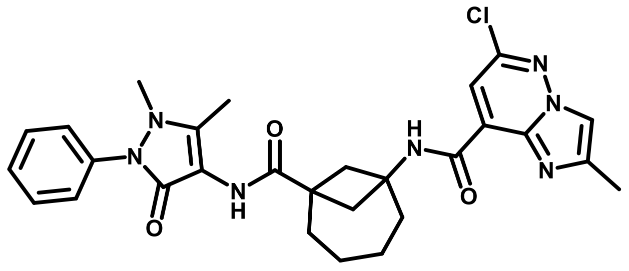

To a mixture of 6-chloro-2-methylimidazo[1,2-b]pyridazine-8-carboxylic acid (28.4 mg, 0.134 mmol) in DMSO (0.45 mL) was added DIPEA (0.093 mL, 0.536 mmol) and HATU (61.1 mg, 0.161 mmol). The reaction mixture was stirred at rt for 15 min before the addition of 6-amino-N-(1,5-dimethyl-3-oxo-2-phenyl-2,3-dihydro-1H-pyrazol-4-yl)bicyclo[4.1.1]octane-1-carboxamide hydrochloride **(35)** (55.0 mg, 0.141 mmol). The reaction was stirred at rt for 16 h. The crude reaction mixture was purified by preparatory HPLC (eluent: 20-100% MeCN in H_2O) to afford 29.6 mg (40% yield) of 6-chloro-N-(6-((1,5-dimethyl-3-oxo-2-phenyl-2,3-dihydro-1H-pyrazol-4-yl)carbamoyl)bicyclo[4.1.1]octan-1-yl)-2-methylimidazo[1,2-b]pyridazine-8-carboxamide **(18)** as a white solid. ^{1}H NMR (400 MHz, DMSO-d_6) δ 9.90 (s, 1H), 8.69 (s, 1H), 8.27 (s, 1H), 7.61 (s, 1H), 7.49 (t, J = 7.8 Hz, 2H), 7.31 (dd, J = 15.7, 7.8 Hz, 3H), 3.02 (d, J = 2.6 Hz, 3H), 2.53 (s, 3H), 2.46 (s, 4H), 2.07 (s, 3H), 1.93 (s, 2H), 1.80 (s, 6H). LCMS (ESI): m/z = 548.3 $[M+H]^+$.

NMR spectra and HPLC traces for key compounds

Compound 1

NMR:

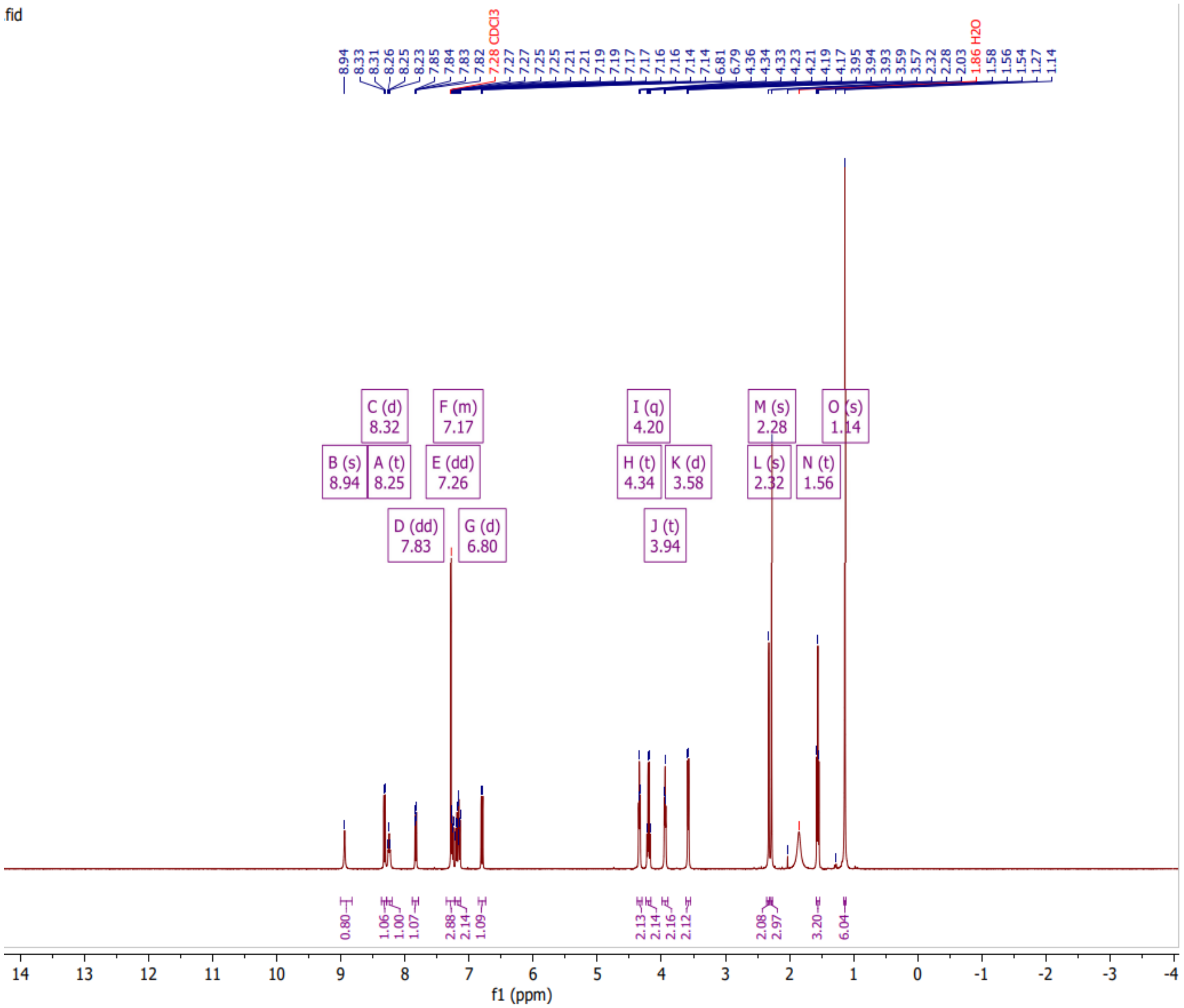

LCMS

HPLC REPORT

Compound ID	: Target A
Sample ID	: EW47902-109-P1X2
Vial#	: 41
Injection Volumn	: 1
Filename	: D:\DATA\2023\2308\230831\EW47902-109-P1X2.lcd
Method Name	: D:\method\10-80AB_3min.lcm
Instrument	: HPLC-098
Run time	: 8/31/2023 20:40:31

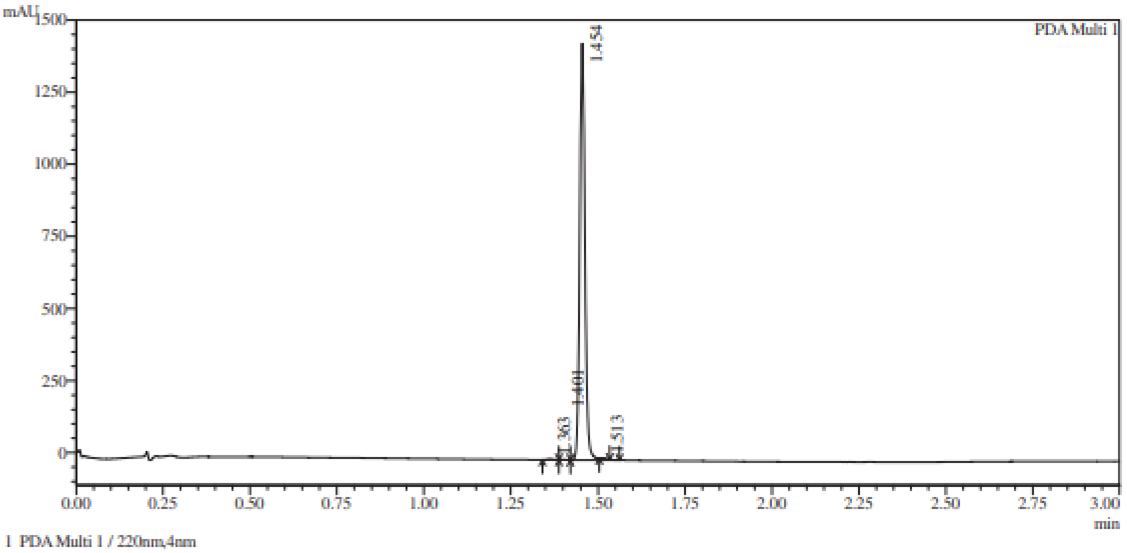

1 PDA Multi 1 / 220nm,4nm

Integration result

PeakTable

PDA Ch1 220nm

Peak#	Ret. Time	USP Width	Resolution	Height	Area	Area %
1	1.363	0.027	0.000	2991	3076	0.202
2	1.401	0.024	1.497	1269	1084	0.071
3	1.454	0.027	2.087	1440563	1516706	99.571
4	1.513	0.022	2.402	2973	2375	0.156
Total				1447796	1523241	100.000

Mass Spectrum
RetTime: 0.502   Datafile: D:\DATA\2023\2308\230831\EW47902-109-P1Y4.lcd

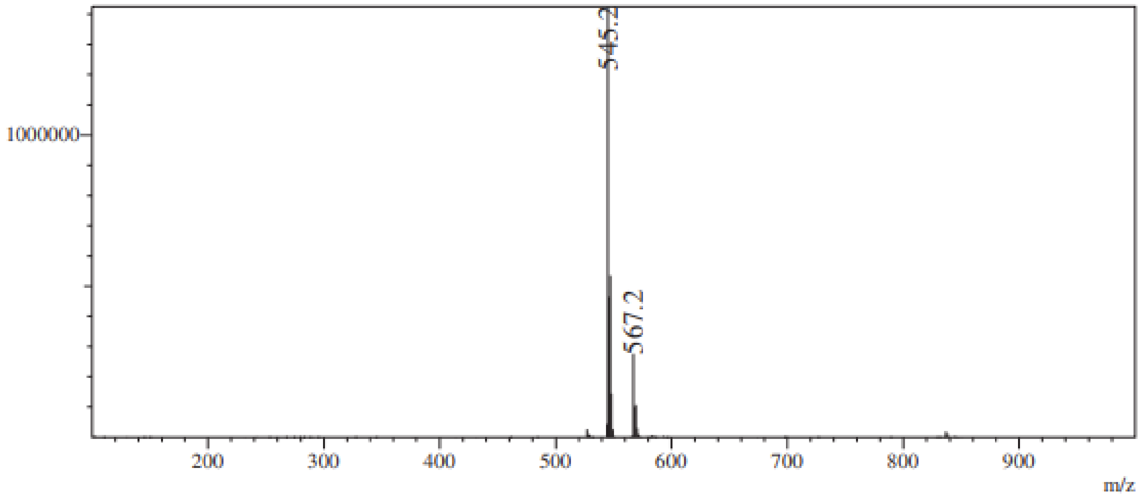

Compound 2

^{1}H NMR:

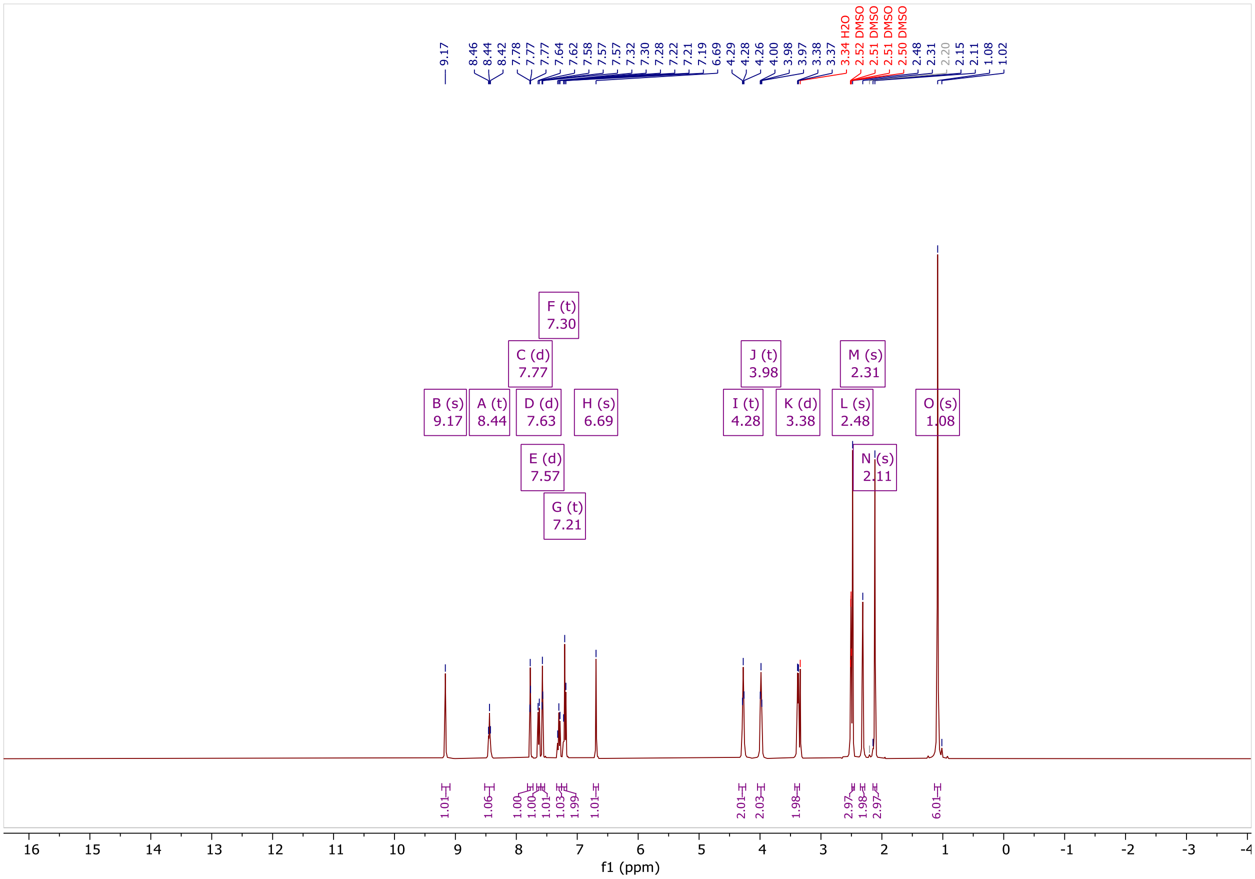

LCMS Trace:

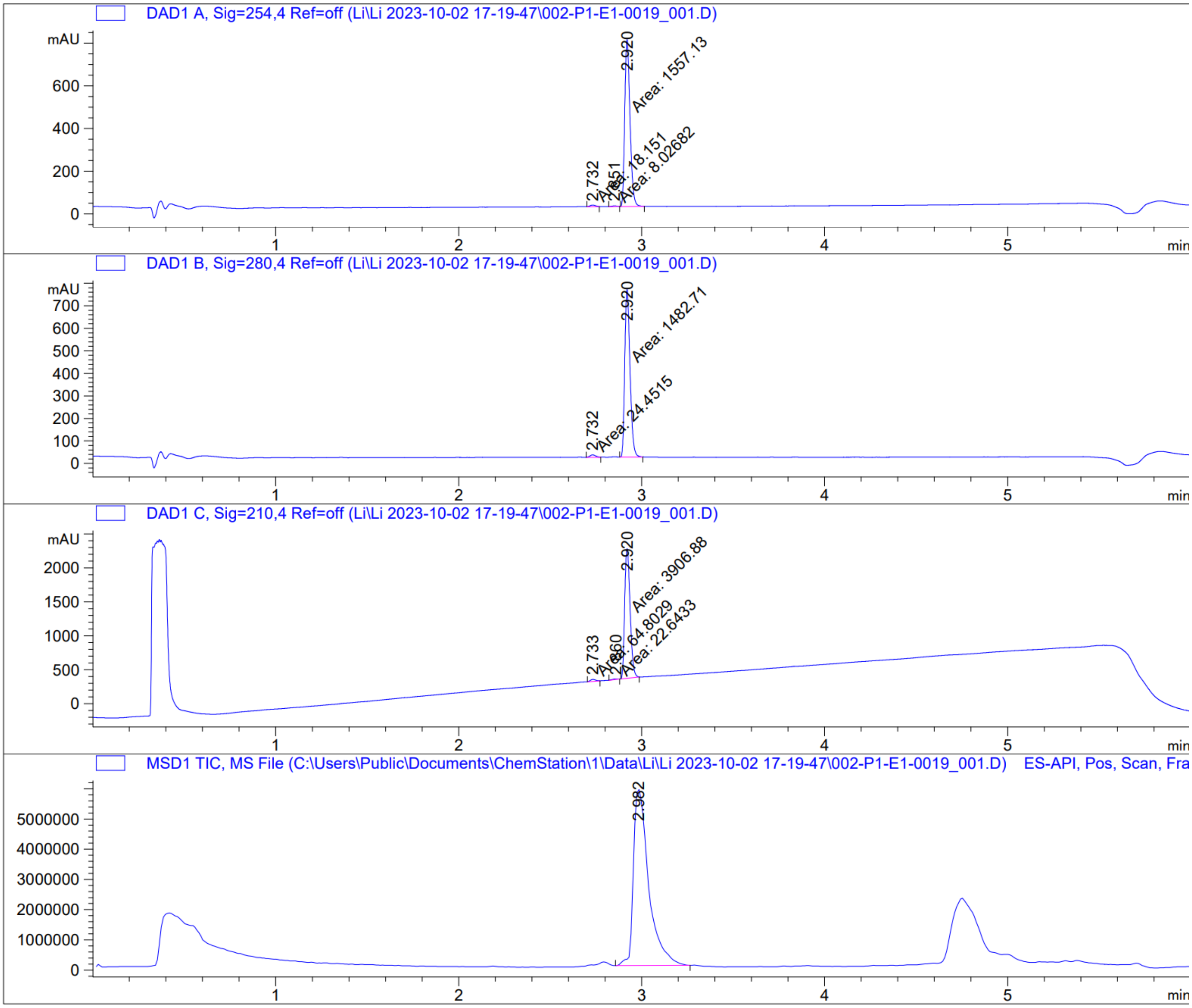
DAD1 A, Sig=254,4 Ref=off (Li\Li 2023-10-02 17-19-47\002-P1-E1-0019_001.D)
mAU
2.920
Area: 1557.13
2.732
Area: 18.151
Area: 8.02682
DAD1 B, Sig=280,4 Ref=off (Li\Li 2023-10-02 17-19-47\002-P1-E1-0019_001.D)
2.920
Area: 1482.71
2.732
Area: 24.4515
DAD1 C, Sig=210,4 Ref=off (Li\Li 2023-10-02 17-19-47\002-P1-E1-0019_001.D)
2.920
Area: 3906.88
2.733
Area: 64.8029
Area: 22.6433
MSD1 TIC, MS File (C:\Users\Public\Documents\ChemStation\1\Data\Li\Li 2023-10-02 17-19-47\002-P1-E1-0019_001.D) ES-API, Pos, Scan, Fra
2.982
min

Area Percent Report

Sorted By : Signal
Multiplier : 1.0000
Dilution : 1.0000
Do not use Multiplier & Dilution Factor with ISTDs

Signal 1: DAD1 A, Sig=254,4 Ref=off

Peak #	RetTime [min]	Type	Width [min]	Area [mAU*s]	Height [mAU]	Area %
1	2.732	MM	0.0369	18.15103	8.20454	1.1464
2	2.851	MM	0.0378	8.02682	3.53811	0.5070
3	2.920	MM	0.0332	1557.13330	782.72418	98.3466
Totals :				1583.31115	794.46683	

Signal 2: DAD1 B, Sig=280,4 Ref=off

Peak #	RetTime [min]	Type	Width [min]	Area [mAU*s]	Height [mAU]	Area %
1	2.732	MM	0.0368	24.45153	11.05912	1.6224
2	2.920	MM	0.0332	1482.71179	744.32349	98.3776
Totals :				1507.16332	755.38261	

Signal 3: DAD1 C, Sig=210,4 Ref=off

Peak #	RetTime [min]	Type	Width [min]	Area [mAU*s]	Height [mAU]	Area %
1	2.733	MM	0.0351	64.80293	30.78189	1.6224
2	2.860	MM	0.0383	22.64332	9.85924	0.5669
3	2.920	MM	0.0341	3906.87646	1907.27905	97.8107
Totals :				3994.32272	1947.92019	

Retention Time (MS)	MS Area	Mol. Weight or Ion
2.982	33829764	540.20 I
		539.20 I
		538.20 I
		537.25 I

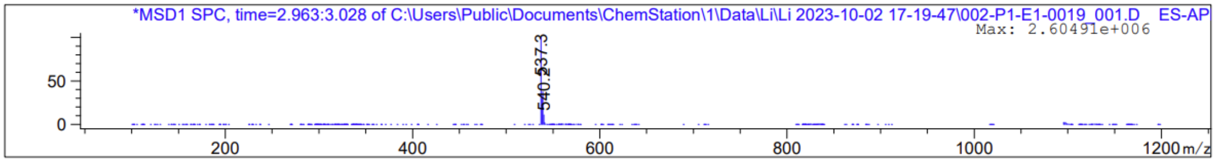

*** End of Report ***

Compound 18

^{1}H NMR:

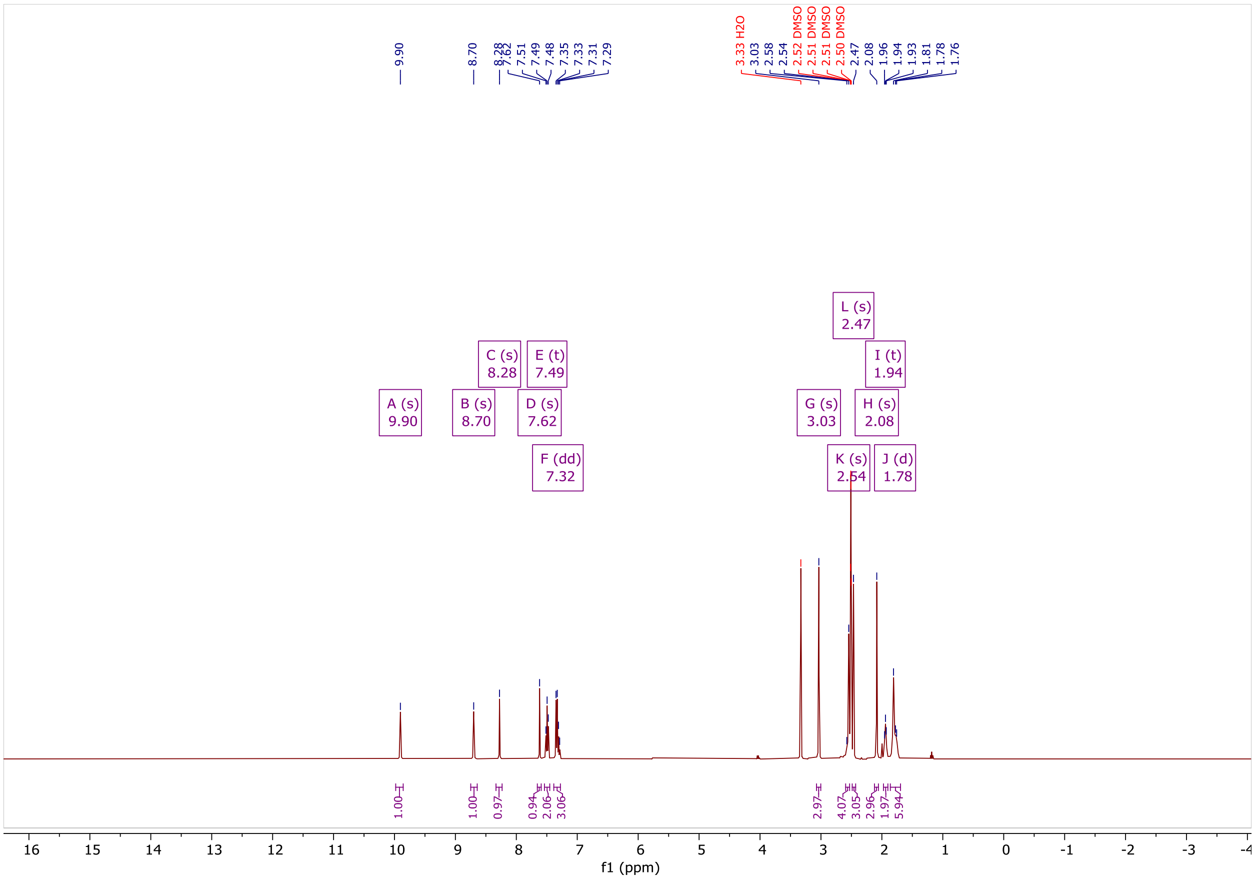

LCMS Trace:

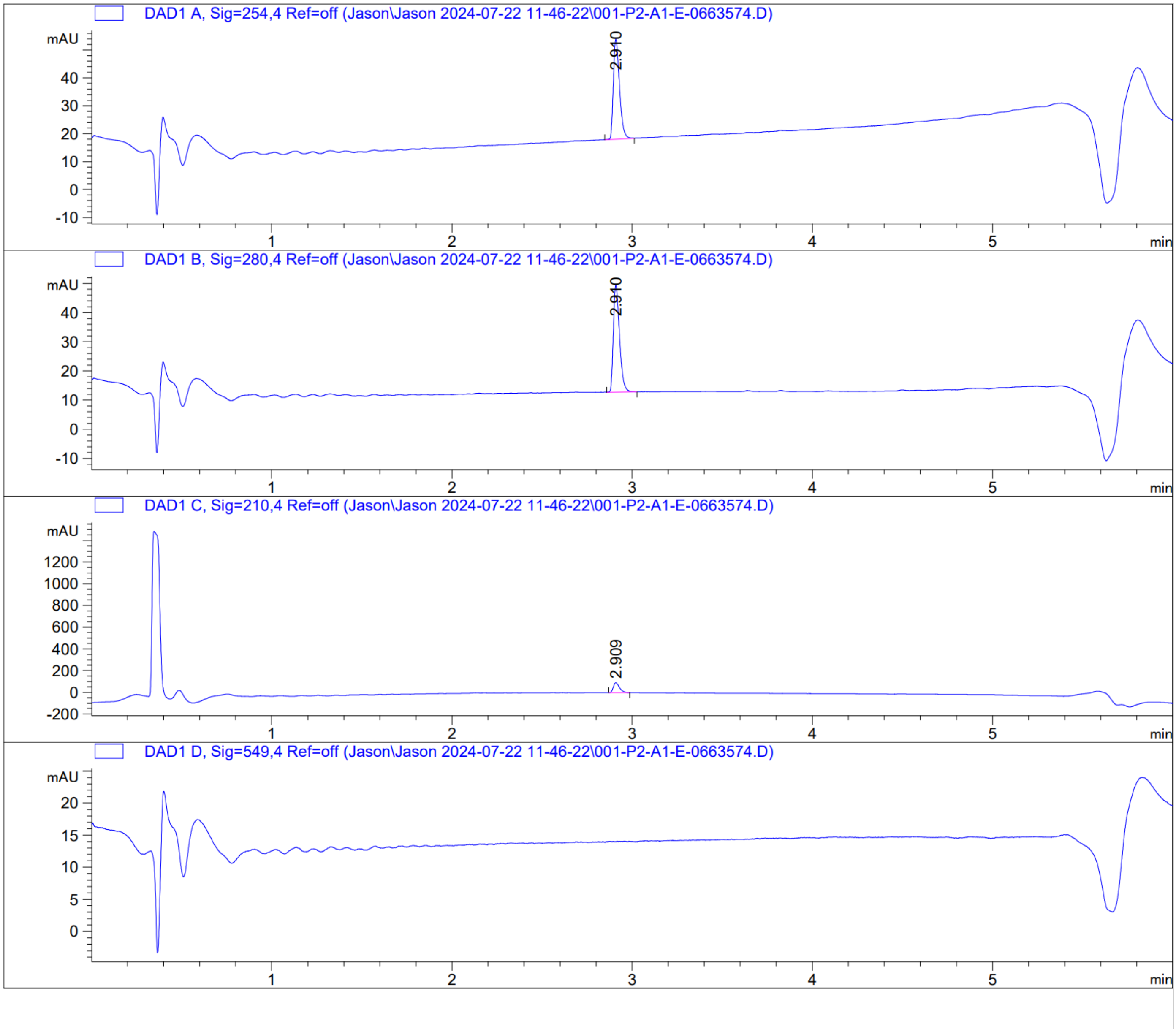
DAD1 A, Sig=254,4 Ref=off (Jason\Jason 2024-07-22 11-46-22\001-P2-A1-E-0663574.D)
mAU
40
30
20
10
0
-10
2.910
1
2
3
4
5
min
DAD1 B, Sig=280,4 Ref=off (Jason\Jason 2024-07-22 11-46-22\001-P2-A1-E-0663574.D)
mAU
40
30
20
10
0
-10
2.910
1
2
3
4
5
min
DAD1 C, Sig=210,4 Ref=off (Jason\Jason 2024-07-22 11-46-22\001-P2-A1-E-0663574.D)
mAU
1200
1000
800
600
400
200
0
-200
2.909
1
2
3
4
5
min
DAD1 D, Sig=549,4 Ref=off (Jason\Jason 2024-07-22 11-46-22\001-P2-A1-E-0663574.D)
mAU
20
15
10
5
0
1
2
3
4
5
min

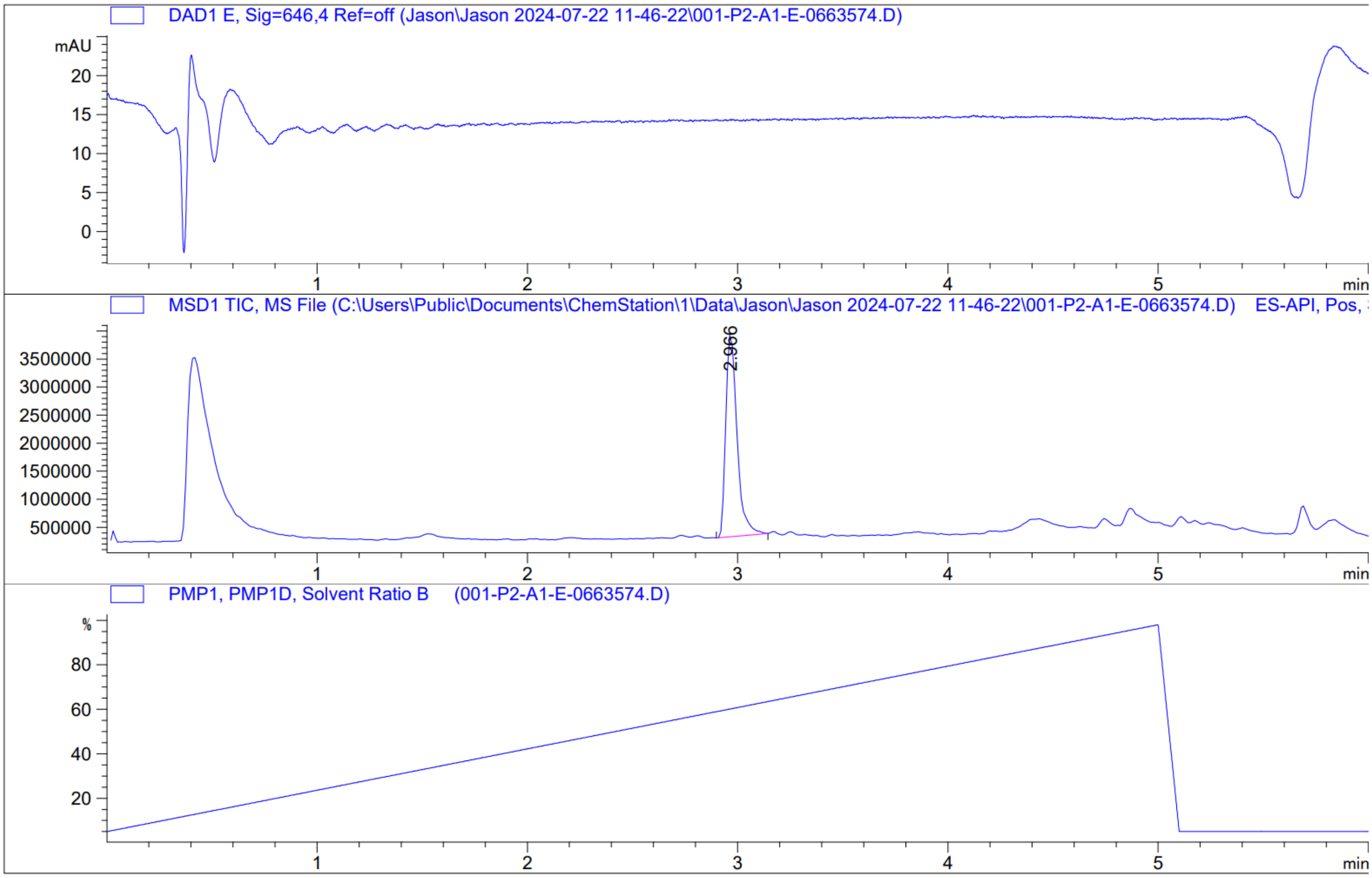
DAD1 E, Sig=646,4 Ref=off (Jason\Jason 2024-07-22 11-46-22\001-P2-A1-E-0663574.D)
mAU
min
MSD1 TIC, MS File (C:\Users\Public\Documents\ChemStation\1\Data\Jason\Jason 2024-07-22 11-46-22\001-P2-A1-E-0663574.D) ES-API, Pos,
2.966
min
PMP1, PMP1D, Solvent Ratio B (001-P2-A1-E-0663574.D)
%
min

Signal 1: DAD1 A, Sig=254,4 Ref=off

Peak #	RetTime [min]	Type	Width [min]	Area [mAU*s]	Height [mAU]	Area %
1	2.910	BB	0.0367	84.89297	35.81059	100.0000

Totals : 84.89297 35.81059

Signal 2: DAD1 B, Sig=280,4 Ref=off

Peak #	RetTime [min]	Type	Width [min]	Area [mAU*s]	Height [mAU]	Area %
1	2.910	BB	0.0368	87.45566	36.68166	100.0000

Totals : 87.45566 36.68166

Signal 3: DAD1 C, Sig=210,4 Ref=off

Peak #	RetTime [min]	Type	Width [min]	Area [mAU*s]	Height [mAU]	Area %
1	2.909	BB	0.0364	213.16652	90.73003	100.0000

Totals : 213.16652 90.73003

Signal 4: DAD1 D, Sig=549,4 Ref=off

Signal 5: DAD1 E, Sig=646,4 Ref=off

Signal 6: MSD1 TIC, MS File

Peak #	RetTime [min]	Type	Width [min]	Area	Height	Area %
1	2.966	BB	0.0562	1.34304e7	3.61871e6	100.0000

Totals : 1.34304e7 3.61871e6

Retention Time (MS)	MS Area	Mol. Weight or Ion
2.966	13430442	1117.40 I
		551.25 I
		550.25 I
		549.25 I
		548.25 I
		275.40 I
		274.70 I

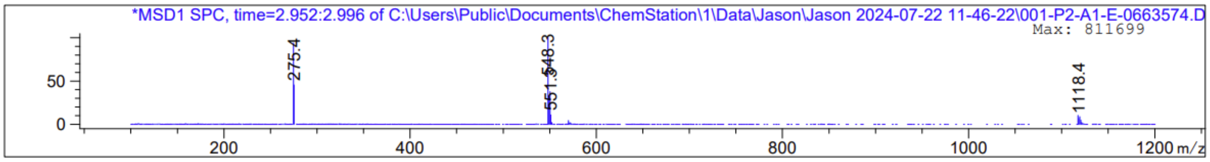

*** End of Report ***
